# Integrative structure of norovirus NS3 suggests a role in RNA transport

**DOI:** 10.1038/s41467-026-77946-7

**Published:** 2026-09-21

**Authors:** Meryl Haas, Tino Hoeksma, Jake T. Mills, Cynthia Kelley, Arnab Das, Kankipati Teja Shyam, Robin Veenstra, Owen Byford, Henrietta Hóf, Coco Niemel, Tim Donselaar, Ieva Drulyte, Frank J. M. van Kuppeveld, David Dulin, Joost Snijder, Morgan R. Herod, Daniel L. Hurdiss

**Affiliations:** 1 Section of Virology, Division of Infectious Diseases and Immunology, Department of Biomolecular Health Sciences, Faculty of Veterinary Medicine, Utrecht University, Utrecht, The Netherlands; 2 Astbury Centre for Structural Molecular Biology, Faculty of Biological Sciences, University of Leeds, Leeds, UK; 3 Biomolecular Mass Spectrometry and Proteomics, Bijvoet Center for Biomolecular Research, Department of Chemistry, Faculty of Science, Utrecht University, Utrecht, The Netherlands; 4 Department of Physics and Astronomy, and LaserLaB Amsterdam, Vrije Universiteit Amsterdam, Amsterdam, The Netherlands; 5 Materials and Structural Analysis, Thermo Fisher Scientific, Eindhoven, The Netherlands

**Keywords:** Virus structures, Cryoelectron microscopy, Molecular biology

## Abstract

Human noroviruses (HuNoVs) are the leading global cause of acute gastroenteritis, yet no vaccines or antiviral therapies are currently approved. The non-structural protein NS3 is a membrane-bound AAA+ ATPase of superfamily 3 (SF3) with multiple proposed roles in the norovirus replication cycle. However, the structure of NS3, and the mechanisms by which it contributes to genome replication and membrane remodeling, have remained unknown. We engineered a soluble, hexameric, and catalytically active form of NS3 and determined its cryo-EM structure in the presence of a nucleotide analogue at 2.9 Å resolution. The structure adopts a split lock-washer architecture characteristic of AAA+ motors that operate via a hand-over-hand translocation mechanism. Complementary biochemical, single-molecule, and virological assays support oligomerization-dependent ATPase activity, ssRNA engagement, and the functional importance of conserved structural elements. Using integrative modeling with AlphaFold3, supported by targeted mutagenesis, we generated a full-length, membrane-associated model in which NS3 forms a continuous conduit across the membrane. This model supports a role for NS3 as a candidate membrane-spanning RNA translocase that may couple ATP hydrolysis to genome movement. This structural and functional framework helps address long-standing gaps in our understanding of norovirus replication and establishes a basis for mechanistic studies and structure-guided antiviral design.

## Abstract (web summary)

Norovirus is the leading global cause of acute gastroenteritis. Here, the authors elucidate norovirus replication and show that the viral NS3 protein forms an RNA-engaging molecular motor that may transport viral RNA during infection.

## Introduction

HuNoVs pose a substantial health burden to society, contributing to ~700 million infections and over 200,000 annual deaths. These viruses are the leading cause of viral gastroenteritis, often referred to as “stomach flu” or “stomach bug,” marked by symptoms, such as sudden onset diarrhea and vomiting^1–3^. Compounding this impact, the global economy suffers an approximate cost of €60 billion annually, encompassing expenses related to lost workdays and healthcare expenditures linked to these infections^4^. Transmission of the virus occurs primarily through the fecal-oral route, facilitated by contact with infected individuals or exposure to contaminated food, water, or infectious aerosols generated by vomiting^2^. This high infectivity and efficient transmission give rise to the potential for newly emerging norovirus strains to spark worldwide epidemics^5^. Notably, despite these risks, there are currently no approved vaccines or antiviral treatments targeting noroviruses^3,6^.

Noroviruses are non-enveloped, positive-sense single-stranded (ss) RNA viruses classified within the genus *Norovirus* and the family *Caliciviridae*. Together with enteroviruses, another notable group of human pathogens, noroviruses belong to the order *Picornavirales*, a diverse assemblage of RNA viruses that infect animals, protists, and plants. Within the genomes of these viruses, there is a characteristic three-domain replication block (Hel-Pro-Pol), which comprises a putative helicase belonging to the SF3 clade of AAA+ ATPases, a chymotrypsin-like proteinase and a superfamily I RNA-dependent RNA polymerase^7,8^. The norovirus protease and polymerase, NS6 and NS7, have been studied by X-ray crystallography, and the molecular basis for their enzymatic activity is understood^9,10^. However, no such information is available for the norovirus NS3, which has been implicated as having multiple functions within the norovirus replication cycle, including: (i) ATP-dependent helicase activity^11^, (ii) ATP-independent RNA chaperoning^12^, (iii) rearrangement of intracellular membrane alterations during replication organelle formation^13,14^, and (iv) the induction of apoptosis^15^.

SF3 helicases typically assemble into hexameric rings and couple ATP hydrolysis to conformational changes that generate mechanical force on nucleic acid substrates^16^. These enzymes possess three conserved motifs essential for nucleotide binding and hydrolysis, Walker A, Walker B, and motif C, all of which are present in norovirus NS3^17,18^. In DNA viruses, SF3 proteins function as replicative helicases, and several oligomeric structures have been resolved^16,19–21^. However, no functional oligomeric assembly of an SF3 protein from an RNA virus has been structurally characterized to date, although crystal structures of picornaviral 2C AAA+ domains have been reported^22–26^. This has left a gap in our mechanistic understanding of the replication machinery employed by positive-strand RNA viruses.

In this work, we present a structure of the norovirus NS3 protein in a nucleotide analogue-bound state. Through integrative modeling, we propose that NS3 functions as a transmembrane RNA translocase, coupling membrane-associated genome replication to RNA transport. Our findings provide a structural framework for understanding SF3 protein function in positive-strand RNA viruses and for exploring how noroviruses replicate and package their genomes. They also provide a basis for future mechanistic studies of NS3 and structure-guided antiviral strategies.

## Results

### Engineering a soluble and enzymatically active NS3 hexamer

Sequence homology with known SF3 proteins and predictive modeling indicate that NS3 comprises an N-terminal membrane-binding domain (NTD), a helical bundle domain (HBD), and a C-terminal AAA+ ATPase domain containing the signature Walker A, Walker B, and motif C required for nucleotide binding and hydrolysis^8^. Previous studies on homologous picornavirus 2C proteins have shown that their N-terminal amphipathic helix renders recombinant proteins poorly soluble but is necessary for efficient formation of enzymatically active hexamers; this requirement has thus far precluded structural studies of SF3 protein quaternary structures from RNA viruses^27,28^. Previously, we demonstrated that enzymatic ATPase activity can be restored in picornaviral 2C protein when arranged in a hexameric complex by replacing the NTD with a synthetic hexamerization domain^24^.

**Fig. 1:**
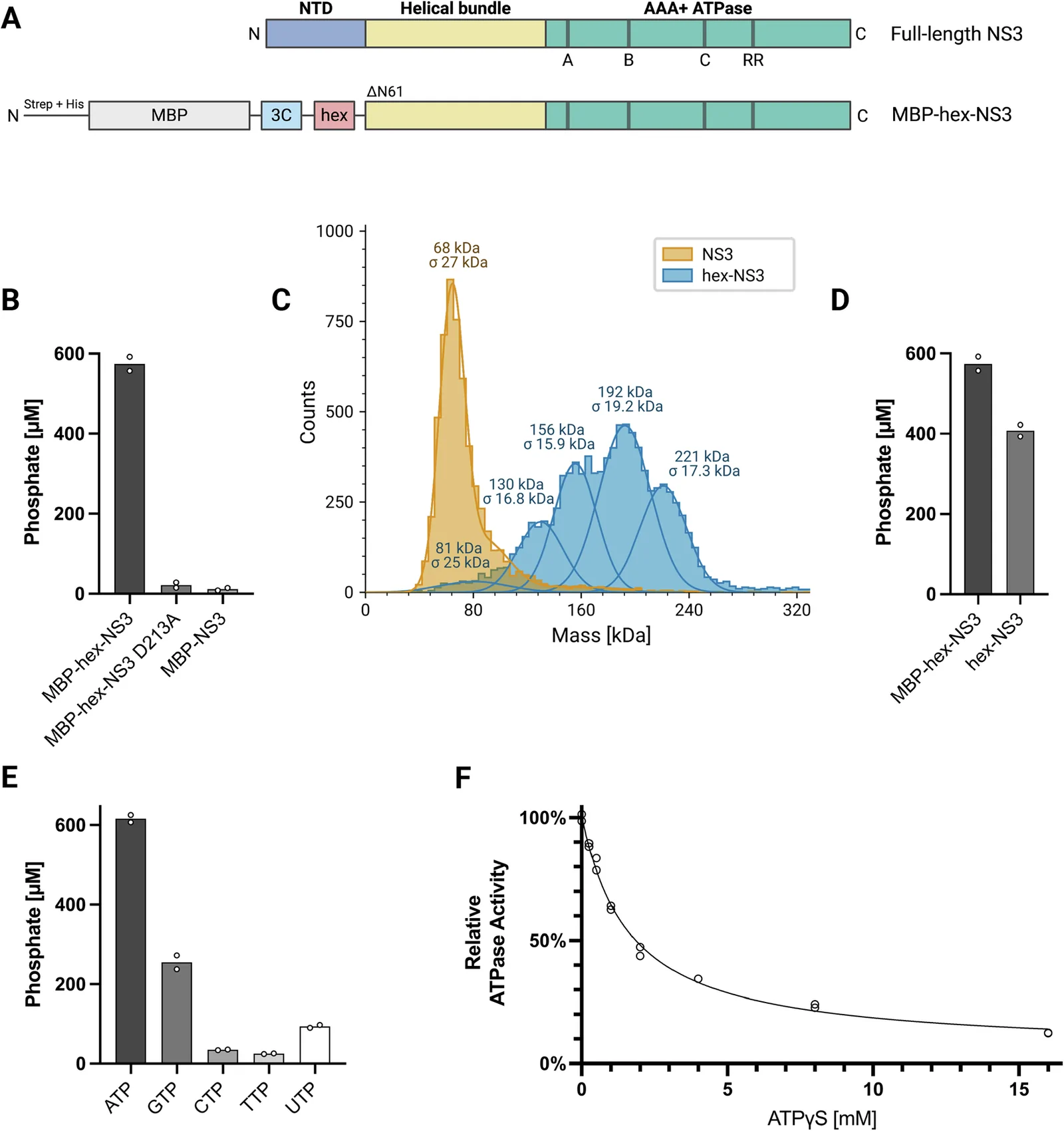
Engineering a soluble, enzymatically active HuNoV NS3 hexamer. **A** Linear representation of the full-length NS3 protein (top) and the engineered hex-NS3 construct (bottom). The full-length NS3 protein comprises a membrane-binding N-terminal domain (NTD), a helical bundle domain (HBD), and an AAA+ ATPase domain comprising Walker A, B, motif C, and arginine fingers (RR). In hex-NS3, the NTD (residues 1–61) is replaced by the 32-residue cc-hex coiled-coil sequence. Created in BioRender. Hurdiss, D. (2026) https://BioRender.com/8xkrtnp. **B** ATPase activity for the WT MBP-hex-NS3, the D213A mutant, and WT MBP-ΔN61-NS3. **C** Mass photometry analysis of ΔN61-NS3 and hex-ΔN61-NS3. For **C**, a single representative measurement (*n* = 1) is shown for each construct. **D** ATPase activity for the MBP-hex-NS3 and following removal of the MBP. **E** NTPase activity of hex-NS3 with different nucleotides. **F** ATPase activity of hex-NS3 in the presence of increasing concentrations of ATPγS. For **B**, **D**–**F**, representative data from one of two independent experiments, each performed in technical duplicate, are shown. Points show technical replicate measurements; bars show the mean. Source data are provided as a Source Data file.

To rationally engineer a soluble recombinant HuNoV NS3 construct that retains the ability to form functional oligomers, we first generated AlphaFold2 predictions of the hexameric complex for HuNoV. These models indicated a suitable truncation site between the NTD and HBD, resulting in a construct lacking the first 61 amino acids of NS3, annotated as ΔN61 (Supplementary Fig. 1A). We then inserted a soluble hexamerization domain, cc-hex^29^, and a linker region before our ΔN61-NS3 construct. To facilitate expression and purification, we added an N-terminal hexa-histidine tag (His6) and an MBP-tag to the N-terminus of our hex-ΔN61-NS3 construct, separated by a 3C protease cleavage site (MBP-hex-NS3, Fig. 1A and Supplementary Fig. 1B). For comparison, we also generated the equivalent ΔN61-NS3 construct without the hexamerization domain (MBP-NS3).

Following expression in *E. coli* and purification (Supplementary Fig. 2), we tested the ATPase activity of our MBP-tagged NS3 constructs using the malachite green assay, which detects free phosphate produced by nucleoside triphosphate hydrolysis. To confirm NS3-specific ATP hydrolysis, we substituted the critical Walker B residue D213 with alanine, which abolished ATPase activity (Fig. 1B). Similarly, no detectable enzymatic activity was observed for the monomeric construct, highlighting the importance of oligomerization for nucleotide triphosphatase activity.

Subsequent removal of the MBP-tag demonstrated that both the monomeric and hexameric constructs remained soluble. To assess the oligomeric states of our NS3 constructs, we employed mass photometry and native mass spectrometry analyses. While NS3 in the absence of the cc-hex domain (33.6 kDa) was found to form primarily monomers and dimers, hex-NS3 (38.2 kDa) formed higher oligomers ranging from trimers to hexamers, highlighting the importance of the N-terminal region for oligomerization in NS3 (Fig. 1C and Supplementary Fig. 3). We then confirmed that the untagged hex-NS3 retains ATPase activity (Fig. 1D). Due to its slightly higher activity and less involved purification protocol, the MBP-tagged construct was used for future NTPase assays described in this study.

The nucleotide specificity of NS3 was tested by providing different nucleoside triphosphates (ATP, GTP, CTP, TTP, and UTP) as substrates. NS3 exhibited a preference for ATP, followed by GTP, with ~3-fold lower activity (Fig. 1E). Notably, this contrasts with earlier studies that reported minimal discrimination between nucleotides by NS3^11^. As a prelude to structural analysis, we assessed whether adenosine 5′-O-(3-thiotriphosphate) (ATPγS) could compete for binding to the orthosteric site and thus serve as a suitable nucleotide analogue to capture the *holo* complex by cryogenic electron microscopy (cryo-EM). Our results demonstrated a dose-dependent decrease in ATP hydrolysis in the presence of increasing concentrations of ATPγS (Fig. 1F), underscoring its utility for structural studies.

### Cryo-EM analysis of the NS3 hexamer

To assess the suitability of our hex-NS3 construct for structural studies, we first evaluated sample quality using negative stain electron microscopy (EM). Prior to grid preparation, hex-NS3 was incubated with ATPγS to stabilize the nucleotide-bound state. Micrographs revealed monodisperse, ring-shaped particles consistent with a hexameric assembly. In addition, we infrequently observed filamentous structures (Supplementary Fig. 4A). Subsequent 2D classification of the ATPγS-bound sample yielded class averages consistent with the expected oligomeric architecture (Supplementary Fig. 4B).

**Fig. 2:**
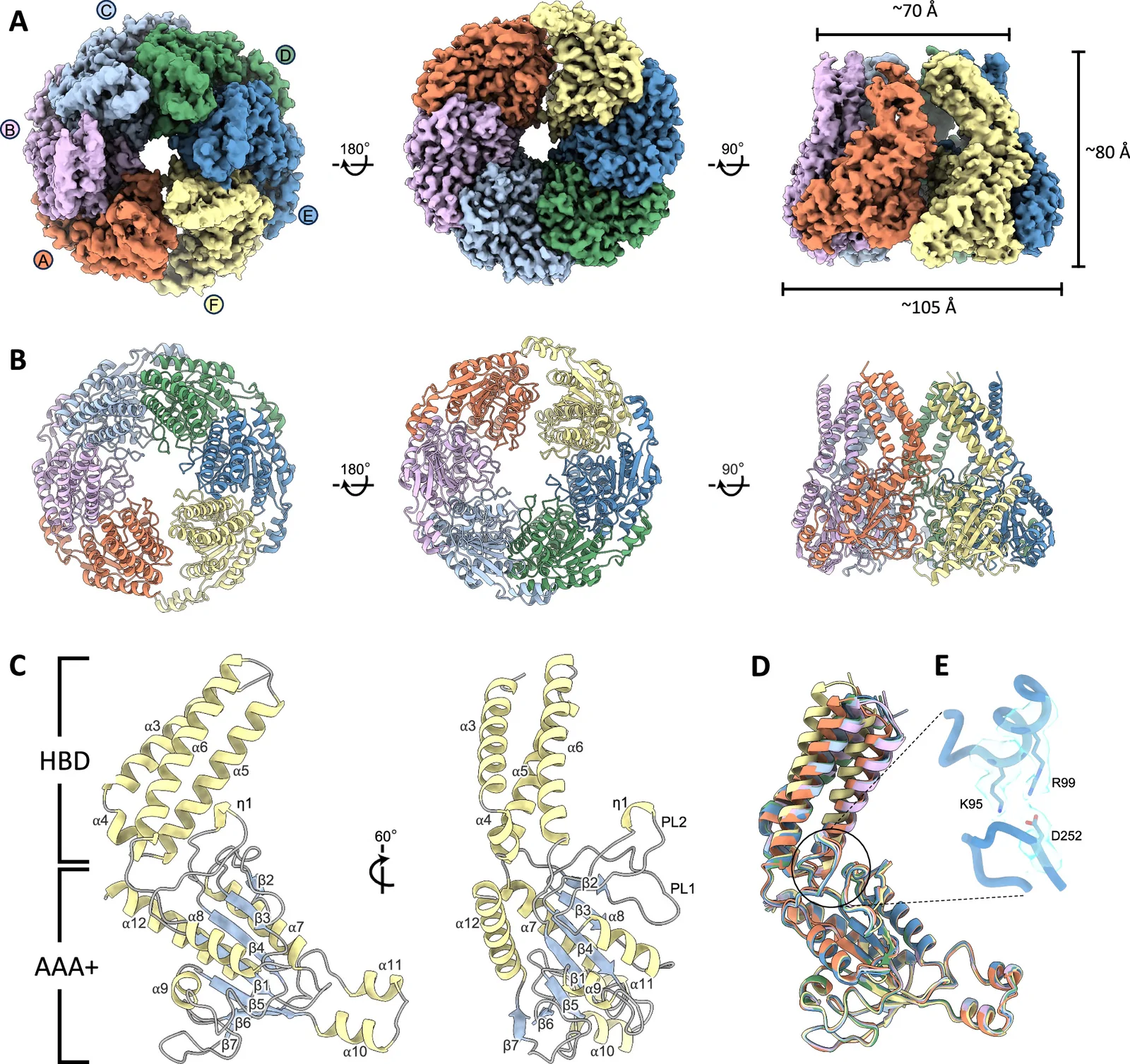
The architecture of the ATPγS-bound NS3 hexamer in a split lock-washer conformation. **A** Three orthogonal views of the EM map of the NS3 hexamer with individual protomers shown in different colors. **B** As shown in (**A**) for the atomic model. The ~6.5 Å gap between protomers A and F is apparent in the first top view. **C** Cartoon representation of subunit C with helices in yellow, strands in blue, and loops in gray. The HBD and AAA+ domains have been labeled. **D** Superposition of the six NS3 protomers. **E** Zoomed-in view of the interface between the HBD and AAA+ domains in subunit E, with residues involved in salt bridge formation shown as sticks and the corresponding EM density shown in blue.

We next prepared cryo-EM grids and determined a three-dimensional reconstruction of the ATPγS-bound hex-NS3 complex at a global resolution of 2.9 Å (Fig. 2A and Supplementary Figs. 5, 6). The resulting structure shows that six copies of NS3 assemble into a funnel-shaped hexamer approximately 80 Å in height. The HBD and AAA+ ATPase domain form upper and lower tiers measuring ~70 and ~105 Å in diameter, respectively. Unlike the C6-symmetric model predicted by AlphaFold2 (Supplementary Fig. 1A), our cryo-EM reconstruction adopts a split lock-washer conformation, previously described in other AAA+ ATPases^16^. The six subunits form a right-handed spiral with a rise of ~1.5 Å per protomer and a gap of ~6.5 Å between the topmost and bottommost subunits. Notably, the HBDs make minimal, if any, lateral contacts with each other.

Next, we generated an atomic model encompassing residues 64–363 across all six subunits (Fig. 2B and Supplementary Fig. 7), as well as 204 putative water molecules. At the tertiary structure level, our cryo-EM model agrees closely with AlphaFold2 predictions. The RMSD across all aligned atoms ranged from 3.2 to 5.4 Å, with even better agreement for the AAA+ domain alone (RMSD 0.9–1.2 Å). Structurally, the HBD adopts a three-helix bundle (residues 64–148), connected to the AAA+ domain by a five-residue linker (Fig. 2C). As expected, residues 154–363 form a canonical Rossmann fold, with all conserved SF3 motifs well-resolved. We also modeled two pore loops: residues 192–207 (pore loop 1) and residues 230–252 (pore loop 2).

Structural homology analysis using Foldseek and DALI confirmed strong similarity between the AAA+ domain and picornaviral 2C proteins, papillomaviral E1 helicases, and Rep helicases from single-stranded DNA viruses. For the NS3 HBD, AtBAG1, a BAG-family co-chaperone from *Arabidopsis thaliana*, was identified as the closest experimentally determined structural homolog^30^. To further explore the evolutionary relationship between NS3 and other viral SF3 helicases, we conducted structure-based phylogenetic analysis using available high-resolution structures of AAA+ domains from SF3 helicases. This analysis showed that NS3 clusters closely with 2C proteins from members of the family *Picornaviridae* (Supplementary Fig. 8), reinforcing their shared ancestry and structural conservation within the order *Picornavirales*.

Finally, RMSD calculations across all six protomers revealed only minor angular deviations in the HBD–AAA+ orientation for subunits A and F at the seam (0.4–0.8 Å), indicating minimal conformational variability within the hexamer (Fig. 2D). The orientation of the HBD relative to the AAA+ domain appears to be stabilized by intramolecular salt bridges between D252 and two lysine residues, K95 and K99 (Fig. 2E).

### Structural basis for ATP binding and hydrolysis

**Fig. 3:**
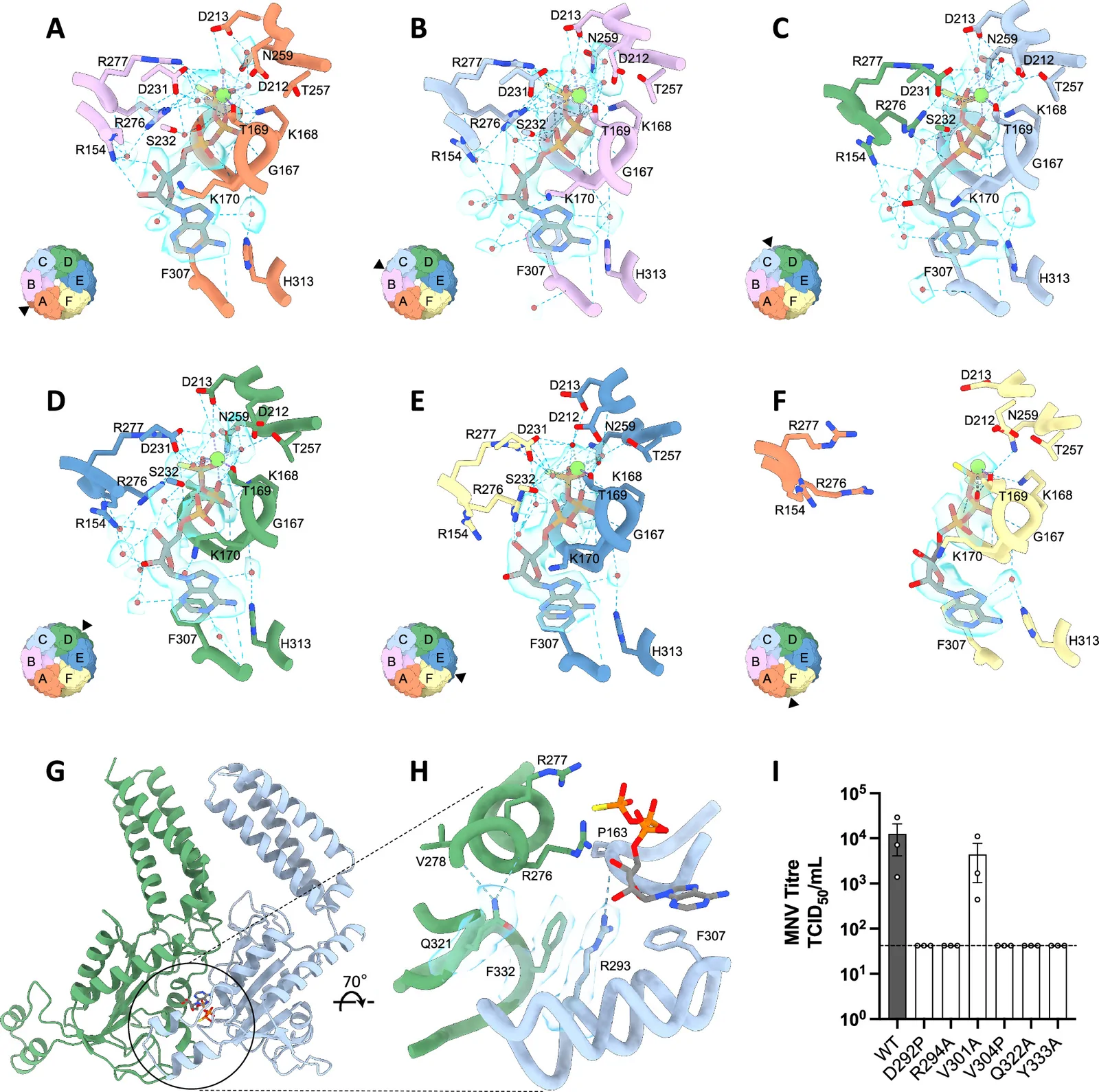
Molecular basis for ATP binding and hydrolysis. **A**–**F** Zoomed-in view of each of the six ATP-binding sites in the NS3 hexamer. The inset panel shows the location relative to the overall structure. **G** Cartoon representation of subunits C and D. **H** Zoomed-in view of the ATP binding site with key residues shown as sticks and their EM density colored blue. **I** Infectious titers of WT and mutant MNV were determined by TCID_50_ assay on BV-2 cells. Bars show the mean ± SEM, with individual points representing independent biological replicates, *n* = 3. Source data are provided as a Source Data file. The dashed line denotes the limit of detection for TCID_50_ assays. Equivalent HuNoV GI and MNV GV NS3 residues are defined by the sequence alignment in Supplementary Fig. 9 and may differ by one position in numbering.

The catalytic site of SF3 ATPases is formed at the interface between two adjacent subunits. One subunit provides the cis-acting Walker A, Walker B, and motif C residues, while the neighboring subunit contributes the trans-acting arginine finger. In our NS3 cryo-EM reconstruction, clear density corresponding to ATPγS is visible in all six nucleotide-binding sites, although it is less well resolved in protomer F, which lies at the seam of the hexamer (Fig. 3A–F). At each site, the triphosphate moiety of ATPγS is coordinated by the P-loop, with the β-phosphate forming interactions with the backbone amides of G165, G167, and K168 (Walker A). The lysine side chain is positioned to interact with both the β- and γ-phosphates. In five of the six sites, resolved to ~2 Å, we observed a Mg²⁺ ion coordinated by the β- and γ-phosphates, the side chain of T169, and three water molecules. Solvent molecules in this primary hydration shell form hydrogen bonds with D213 (Walker B) and D231 (part of pore loop 2). A putative water molecule located between N259 and D213 in these five sites is likely positioned for nucleophilic attack on the γ-phosphate (Fig. 3A–D).

Subunits A–E show the γ-phosphate interacting with R276 and R277 (arginine finger), as well as with N259 (motif C), supporting their classification as ATP-bound. In contrast, subunit F adopts an apo-like conformation, as the arginine finger is displaced across the seam and does not engage the bound nucleotide (Fig. 3F). Additional stabilizing interactions in the ATP-bound subunits include a hydrogen bond between the ribose 3′ hydroxyl of ATPγS and R154. At the base recognition level, the adenine ring is sandwiched between K170 and F307, stabilized through cation–π and π–π stacking interactions. A putative water molecule is also observed forming hydrogen bonds with the ATPγS imidazole ring, the backbone carbonyl of G167, and the side chain of H313. The nucleotide-binding pocket is further reinforced by a cation–π interaction between R293, located in a helix–turn–helix motif, and F332 in the neighboring protomer. Upstream, Q321 engages in hydrogen bonds with the backbone atoms of V278 and R276, helping to stabilize the helix that presents the arginine finger (Fig. 3G, H).

Due to the unavailability of infectious clones for HuNoV, to validate the functional relevance of these residues, we introduced corresponding mutations into the NS3 protein of murine norovirus (MNV) and assessed viral infectivity (Supplementary Fig. 9). Mutations designed to disrupt the cation–π interaction or destabilize the helix-turn-helix motif resulted in complete loss of infectivity and reduced viral RNA accumulation by qRT-PCR, consistent with an impairment in genome replication and with the structural role these residues play in catalysis (Fig. 3I and Supplementary Fig. 10A). In line with this, alanine substitutions at these residues strongly reduced or abolished ATPase activity in the HuNoV hex-NS3 construct (Supplementary Fig. 10B).

### Architecture of the NS3 pore loops

**Fig. 4:**
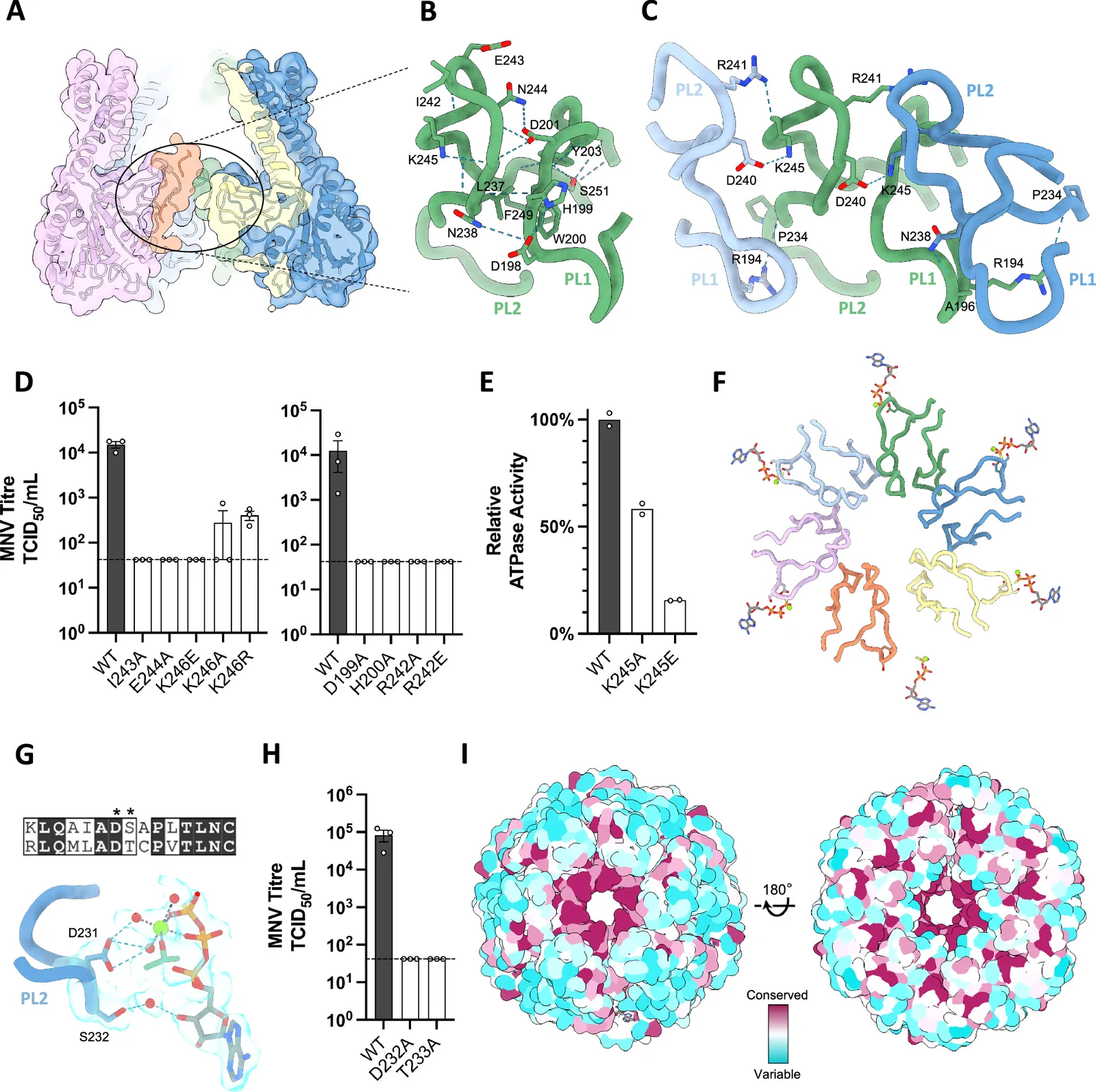
The architecture of the NS3 pore loops. **A** Central slice through the surface representation of the NS3 hexamer. **B** Zoomed-in view of pore loops (PL) 1 and 2 from subunit D, with residues involved in intramolecular interactions shown as sticks. **C** As shown in (**B**) for subunits C–E, with residues involved in inter-protomer interactions shown as sticks. **D** Infectivity of WT MNV and pore loop mutants. **E** ATPase activity of the WT, K245A, and K245E NS3 mutants. **F** Cartoon representation of the NS3 pore loops, with ATPγS shown as sticks. **G** Zoomed-in view of residues 227–234 of protomer E and the ATPγS molecule located in the DE active site. **H** Infectivity of WT MNV compared to the D232A and T233A NS3 mutants. **I** Two orthogonal views of the NS3 hexamer are shown as a surface representation and colored according to conservation across 46 NS3 sequences. For **D**, **H**, bars show the mean ± SEM, with individual points representing independent biological replicates, *n* = 3. For **E**, representative data from one of two independent experiments, each performed in technical duplicate, are shown. Points show technical replicate measurements; bars show the mean. Source data are provided as a Source Data file.

Within our NS3 structure, we identified two pore loops, both of which were resolved at approximately 2–3 Å resolution in our cryo-EM reconstruction. These loops are located beneath a solvent-accessible chamber formed by the HBDs, with an estimated volume of 19,400 Å³ (Fig. 4A). Pore loop 2 defines a central channel approximately 15 Å in diameter, sufficient to accommodate single-stranded RNA, into which the hydrophobic side chain of I242 projects. The two pore loops are stabilized by inter-protomer hydrogen bonding and packing of side chains W200, L237, and F249. At the subunit level, pore loops 1 and 2 are closely associated, forming an average interface area of approximately 420 Å² (Fig. 4B). With the exception of subunits A and F, located at the seam of the hexamer, pore loops from adjacent subunits form inter-subunit contacts, averaging ~360 Å^2^ in interface area. These contacts are mediated by electrostatic interactions between D240 and K245, as well as intermolecular hydrogen bonds involving the side chains of R194 and R241 with the backbone carbonyls of P234 and K245, which collectively help stabilize the central pore architecture (Fig. 4C).

To functionally validate the importance of these structural features, we introduced targeted mutations into the MNV NS3 pore loops. In line with our structural observations, most mutations completely abolished viral infectivity, whereas only K246A and K246R yielded low levels of infectious virus (Fig. 4D). Analysis of viral RNA accumulation by qRT-PCR showed that these pore-loop mutations also reduced genome amplification (Supplementary Fig. 10A), indicating that the observed defects were not restricted to virus release or spread.

To further assess the biochemical impact of these residues, we introduced the corresponding mutations into the recombinant HuNoV hex-NS3 construct and measured ATPase activity. Alanine substitutions at key pore-loop residues strongly reduced or abolished ATP hydrolysis (Supplementary Fig. 10B). In the case of K245, both K245A and K245E impaired ATPase activity, with the charge-reversing K245E mutation producing the stronger defect (Fig. 4E). Together, these virological and biochemical data support the functional importance of the predicted pore-loop architecture.

In five of the six ATP-binding sites, pore loop 2 residues D231 and S232 form solvent-mediated hydrogen bonds with the bound ATPγS molecule (Fig. 4F, G). In contrast, in subunit A, positioned at the top of the spiral near the seam, pore loop 2 is disengaged. This suggests it may be poised to sense the incoming ATP molecule in the adjacent subunit. Together, these observations indicate that coordination between the pore loops and the nucleotide-binding sites plays a central role in orchestrating sequential ATP hydrolysis across the hexamer (Supplementary Fig. 11A). Consistent with this model, alanine substitutions at these residues abolished infectivity in the MNV system and eliminated ATPase activity in the HuNoV hex-NS3 construct (Fig. 4H and Supplementary Fig. 10B). Sequence analysis using ConSurf, based on 46 calicivirus NS3 sequences, revealed strong conservation of residues in the pore loops and catalytic site. Together with our mutational data, this supports their functional importance and highlights their potential as targets for pan-genotypic antiviral intervention (Fig. 4I).

### NS3 engages ssRNA in an oligomerization-dependent manner

**Fig. 5:**
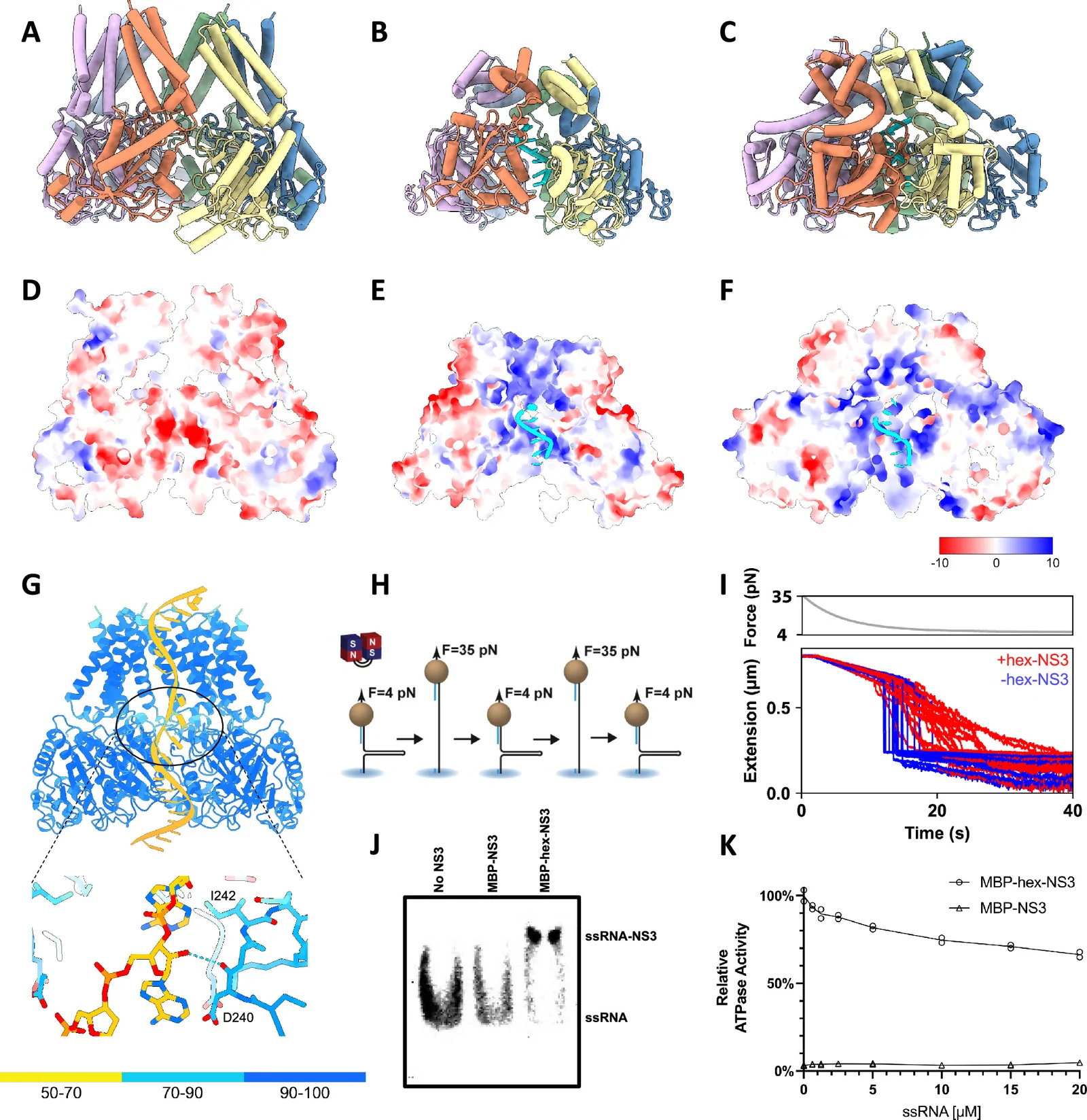
NS3 engages ssRNA in an oligomerization-dependent manner. Cartoon representation of the **A** NS3 hexamer, **B** porcine circovirus 2 rep protein bound to single-stranded DNA (PDB ID: 7LAR)^20^, and **C** papillomavirus E1 hexameric helicase with ssDNA (PDB ID: 2GXA)^19^. **D** Central slice through the NS3 hexamer shown as a surface representation and colored according to electrostatic surface potential. **E** As shown in (**D**) for porcine circovirus 2. **F** As shown in (**D**) for papillomavirus E1. **G** AlphaFold3 prediction of the ΔN61-NS3 hexamer in complex with a 50-nt poly(A) ssRNA substrate, colored by pLDDT confidence. The lower panels show close-up views of recurrently observed features in the RNA-bound models. **H** Schematic of the magnetic tweezers hairpin refolding assay used to monitor ssRNA engagement by NS3 constructs. **I** Representative ssRNA hairpin refolding traces in the absence of ATP, measured without protein (blue, *n* = 17) or with hex-NS3 (red, *n* = 17). The upper panel shows the applied force protocol. **J** EMSA analysis of ssRNA binding by NS3 constructs. The EMSA shown in (**J**) is representative of two independent experiments with similar results. **K** ATPase activity of hex-NS3 and NS3 lacking the hexamerization domain in the presence of increasing ssRNA concentrations, normalized to the no-RNA condition. For **K**, representative data from one of two independent experiments, each performed in technical duplicate, are shown. Points show technical replicate measurements; bars show the mean. Source data are provided as a Source Data file.

Structural comparison of the NS3 complex with SF3 helicases from single- and double-stranded DNA viruses revealed several notable differences. In NS3, HBDs, which form the walls of the chamber above the AAA+ pore loops, exhibit minimal or no interactions with one another (Fig. 5A). In contrast, the corresponding regions in previously characterized structures of the Rep and E1 SF3 helicases form a tightly packed collar that remains planar during the translocation of single-stranded DNA (Fig. 5B, C)^19,20^. In these DNA helicases, the collar not only stabilizes oligomeric assembly but also serves as a mechanical anchor point for the conformational changes of the AAA+ domains^19,20^. By contrast, in NS3, the HBDs and AAA+ domains adopt a shared right-handed helical configuration, suggesting they function synchronously as a unified motor.

Another distinguishing feature of NS3 is the pronounced negative electrostatic potential of its central pore (Fig. 5D). This contrasts with the positively charged central channels of Rep and E1 (Fig. 5E, F)^19,20^, which are electrostatically well-suited to engage the phosphate backbone of DNA. While the negative charge would be incompatible with stable nucleic acid binding in classical helicase function, it is reminiscent of the electrostatic environment observed in other viral nucleic acid translocation systems, where transient interactions may facilitate efficient translocation without stalling^31–33^. Moreover, in contrast to SF3 helicases from DNA viruses, NS3 lacks a well-ordered canonical PS1βH β-hairpin. Instead, pore loop 2 occupies a putative substrate-facing position within the central channel (Supplementary Fig. 11B–D).

To explore how this pore-loop architecture might support nucleic acid engagement, we used AlphaFold3^34^, which became available during preparation of this manuscript, to model the hexameric ΔN61-NS3 construct in the presence of ssRNA or ssDNA. Predictions with a 50-mer poly(A) ssRNA repeatedly placed RNA within the central pore (Fig. 5G and Supplementary Fig. 12A, C), whereas equivalent ssDNA substrates were not threaded (Supplementary Fig. 12B, D). Although AlphaFold3 did not reproduce the asymmetric NS3 hexamer observed by cryo-EM, and the RNA itself was predicted with low local confidence, two features were recurrently observed in the RNA-bound models. First, the hydrophobic pore loop 2 residue I242 was positioned close to the RNA path within the central pore, consistent with a possible role as a substrate-contacting residue. Second, the ribose 2′-OH was positioned near the backbone carbonyl of pore-loop residue D240 (Fig. 5G), suggesting that NS3 may preferentially engage RNA over DNA.

We tested this experimentally using magnetic tweezers hairpin refolding assays (Fig. 5H). The hex-NS3 construct caused a pronounced delay in ssRNA hairpin refolding (Fig. 5I and Supplementary Fig. 13A, B), consistent with engagement of the exposed ssRNA segment. A similar, but weaker, effect was observed with ssDNA hairpins (Supplementary Fig. 13D, E, G). In contrast, the isolated synthetic hexamerization peptide did not delay ssRNA or ssDNA refolding, indicating that the effect is not caused by the hex peptide alone (Supplementary Fig. 13C, F).

We next examined whether the NS3 construct lacking the hexamerization domain could engage ssRNA under nucleotide-containing conditions. In magnetic tweezers assays performed in the presence of ATP, ΔN61-NS3 showed only weak and inconsistent effects on ssRNA hairpin refolding (Supplementary Fig. 13H, I), in contrast to the reproducible delay observed with hex-NS3 (Fig. 5I). Consistent with this, EMSA experiments showed formation of shifted ssRNA complexes with hex-NS3, but not ΔN61-NS3 (Fig. 5J and Supplementary Fig. 13J).

Finally, we examined how ATP influences RNA engagement and hydrolysis in the context of the hexameric construct. Addition of ATP reduced the hex-NS3-induced delay in ssRNA hairpin refolding (Supplementary Fig. 13K), suggesting that nucleotide binding and/or hydrolysis modulates the RNA-bound state. In bulk ATPase assays, ssRNA did not stimulate ATP hydrolysis by hex-NS3, but instead caused a modest dose-dependent reduction in activity. The ΔN61-NS3 construct showed no detectable ATPase activity even in the presence of ssRNA at the highest concentration tested (Fig. 5K). Together, these data indicate that robust ssRNA engagement and ATPase activity depend on higher-order oligomerization, but that RNA binding is not productively coupled to ATP turnover in our soluble hexameric construct under these conditions.

We next tested whether hex-NS3 exhibits RNA duplex unwinding activity. In magnetic tweezers experiments performed with RNA hairpin substrates in the presence of ATP, we did not detect hairpin unwinding by the hexameric construct, even under assisting force (Supplementary Fig. 14A). Similarly, no unwinding activity was observed for any NS3 construct in a gel-based assay using fluorescently labeled RNA duplex substrates with a 3′ overhang (Supplementary Fig. 14B).

Collectively, these findings indicate that hex-NS3 binds single-stranded RNA in an oligomerization-dependent manner but lacks detectable RNA duplex unwinding activity under the conditions tested.

### An integrative model of the full-length NS3 hexamer

**Fig. 6:**
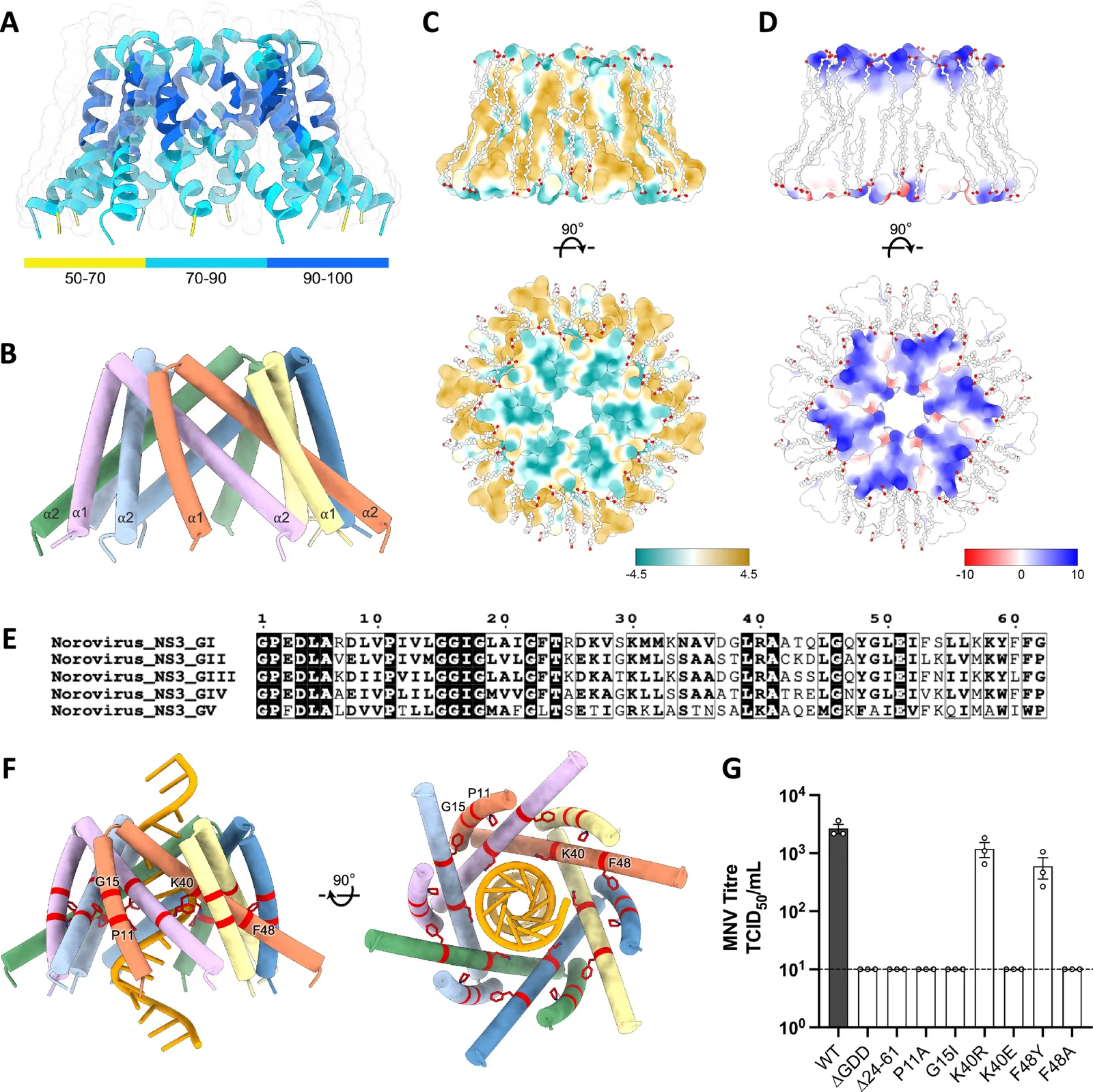
AlphaFold3 predicts a conserved N-terminal transmembrane pore in full-length HuNoV NS3. **A** Cartoon representation of residues 1–61 from the AlphaFold3 prediction of the full-length HuNoV NS3 hexamer in the presence of 50 oleic acid molecules. The model is colored by confidence score (predicted local distance difference test, pLDDT), with blue indicating high-confidence regions and yellow indicating low-confidence regions. **B** Cylindrical representation of the model shown in (**A**), with the two transmembrane helices labeled and colored by chain. **C** Surface representation of the NS3 transmembrane pore, colored according to hydrophobicity using the Kyte-Doolittle scale, shown in two orthogonal views. **D** As in (**C**) but colored by electrostatic surface potential. **E** Sequence alignment of NS3 residues 1–62 from representative norovirus genogroups I–V, highlighting conservation within the NTD. **F** As shown in (**B**), with residues selected for mutational analysis colored red. Side and top views are shown, with the pore-traversing RNA segment displayed as a cartoon to indicate the proposed path through the pore. **G** Recovery of infectious MNV from genomes carrying the indicated NS3 mutations, measured by TCID_50_/mL. Bars show the mean ± SEM, with individual open circles representing independent biological replicates, *n* = 3. The dashed line indicates the assay detection limit. Source data are provided as a Source Data file.

As our hex-NS3 construct lacks the authentic NTD, the architecture of the full-length, membrane-associated complex remained unresolved. We therefore used AlphaFold3 to model the full-length NS3 hexamer. As noted above, AlphaFold3 did not recapitulate the asymmetric lock-washer arrangement observed by cryo-EM, instead predicting a more symmetric assembly. The NTD was initially predicted with low confidence, with most residues assigned pLDDT scores below 50 (Supplementary Fig. 15A, C). Given that the NS3 N-terminus is reported to interact with cellular membranes^14^, we hypothesized that including fatty acid molecules in the input could improve the modeling by mimicking a lipid bilayer environment. Indeed, supplementing the prediction with 50 oleic acid molecules resulted in markedly improved confidence for the NTD, with pLDDT values ranging from 70 to 100 for both HuNoV and MNV (Fig. 6A and Supplementary Fig. 15B, D).

This model suggests that the NS3 NTD comprises two transmembrane helices (α1 and α2). Within the hexameric context, these helices intercalate to form a funnel-shaped transmembrane channel (Fig. 6B). The height of this region is ~40 Å, consistent with the thickness of a typical lipid bilayer. The outer surface of the NTD is hydrophobic, while both the cytosolic and luminal surfaces show positive electrostatic potential, features consistent with membrane integration and lipid headgroup interaction. The channel’s interior, with a diameter of ~15 Å, is positively charged (Fig. 6D), potentially favoring interactions with the negatively charged backbone of RNA. Subsequent predictions across norovirus genogroups I–V revealed a conserved NTD architecture (Fig. 6E and Supplementary Fig. 15E). Predictions with increasing NS3 copy number further showed that interface confidence peaked at the hexameric state across all five genogroups, supporting a conserved preference for hexameric assembly (Supplementary Fig. 15F). In contrast, attempts to reproduce the previously proposed MLKL-like fold of the NS3 NTD^35^, using analogous truncation-based predictions, yielded low-confidence and inconsistent models across the same sequences (Supplementary Fig. 15G).

To test the hypothesis that NS3 forms a continuous conduit for RNA, we repeated the AlphaFold3 prediction of full-length NS3 with the addition of a 50-mer poly(A) ssRNA. Notably, the resulting model positioned the RNA along the central axis of the complex, running uninterrupted through the predicted N-terminal transmembrane channel (Fig. 6F and Supplementary Fig. 16A). This configuration supports the idea that NS3 can accommodate an extended RNA substrate and suggests a continuous RNA path through the hexameric assembly, consistent with a threading or translocation mechanism.

To functionally assess the predicted hexameric NTD architecture of NS3, we designed mutations targeting key structural features and introduced them into an MNV infectious clone (Fig. 6E, F). These included mutations predicted to disrupt interhelical packing within the NTD pore-forming bundle (G15I and F48A), a charge-reversal mutation at a conserved pore-facing residue (K40E), mutation of a conserved proline predicted to support the local transmembrane architecture, and a deletion removing residues 24–61. We also included conservative substitutions predicted to preserve the local structure, including F48Y and K40R. Consistent with the model, mutations predicted to disrupt the NTD architecture or pore electrostatics prevented recovery of infectious virus, whereas substitutions predicted to be tolerated remained viable (Fig. 6G).

To extend these observations to related proteins, we performed similar AlphaFold3 predictions with the full-length 2C protein of Coxsackievirus B3. The resulting model closely resembled the NS3 prediction, also featuring a transmembrane channel aligned with a central RNA conduit that traverses the entire complex (Supplementary Fig. 16B). These findings are consistent with a conserved architectural and potentially mechanistic theme among SF3 ATPases from viruses in the order *Picornavirales*.

**Fig. 7:**
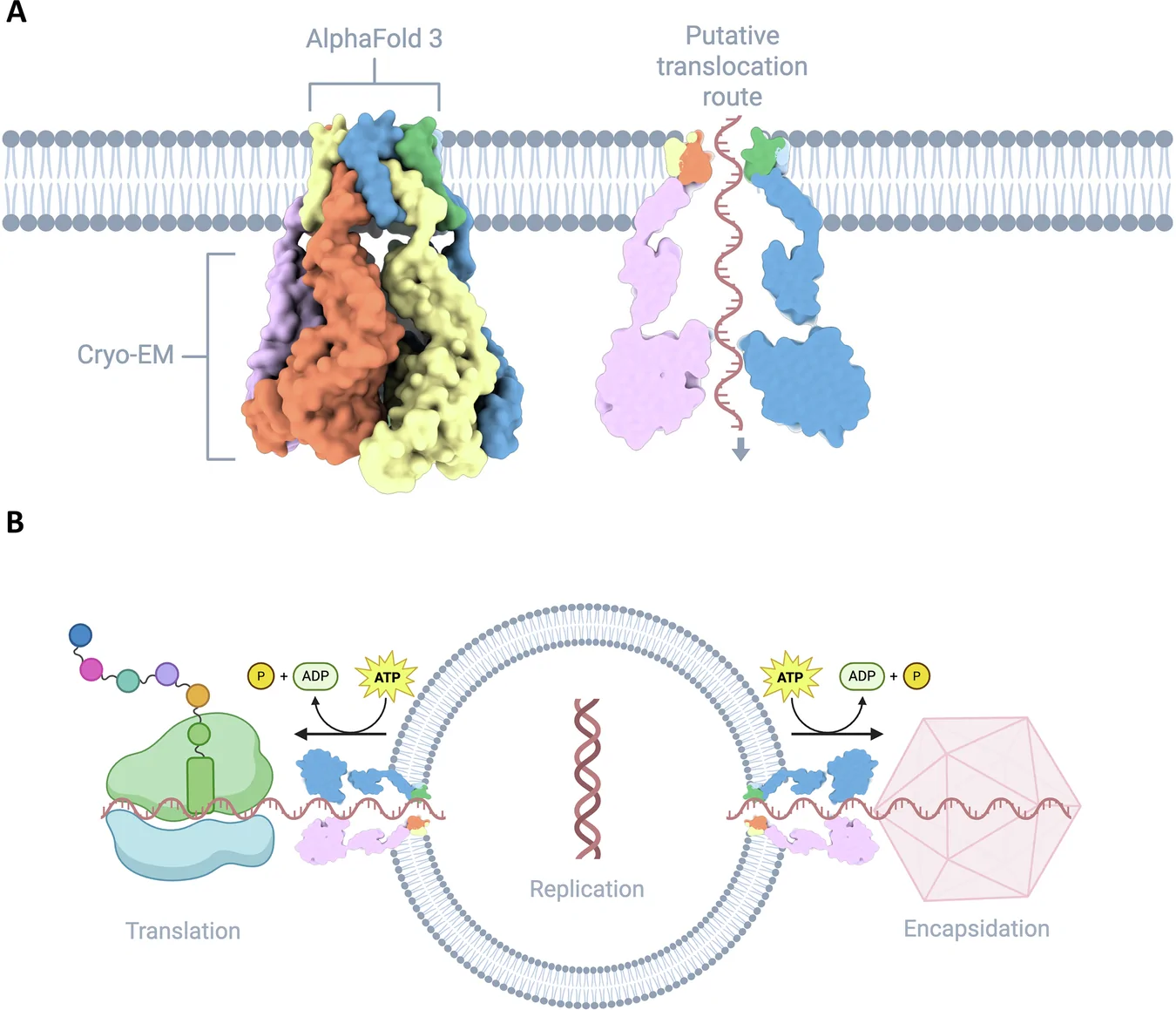
Proposed membrane-associated model for full-length NS3-mediated RNA transport. **A** Surface representation of an integrative model of the full-length NS3 complex embedded in a membrane bilayer (left), and a central cross-section (right) highlighting a putative RNA translocation pathway. Created in BioRender. Hurdiss, D. (2026) https://BioRender.com/6a0zadu. **B** Proposed model for the role of NS3 in exporting ssRNA from single-membrane vesicles for subsequent translation or encapsidation. Created in BioRender. Hurdiss, D. (2026) https://BioRender.com/mvagjct.

To generate a structural model of the full-length, membrane-embedded NS3 hexamer, we integrated our cryo-EM reconstruction of the AAA+ and HBD regions with the AlphaFold3-derived NTD prediction (Fig. 7A). This model suggests that NS3 may associate with replication organelle membranes through its N-terminal transmembrane helices. In this configuration, a coordinated ATPase cycle could support hand-over-hand movement of RNA through the central channel and across the membrane (Supplementary Movie 1), potentially enabling delivery of viral RNA to the cytoplasm for translation and encapsidation (Fig. 7B).

## Discussion

Despite the clinical and economic impact of human noroviruses, many aspects of their replication cycle remain poorly understood^3,36^. The non-structural protein NS3 has been implicated in several key processes during infection, including a putative role as an RNA chaperone or helicase^11,12^. However, until now, the oligomeric structure of NS3, or of any SF3 ATPase from an RNA virus, had not been resolved.

Replication of norovirus RNA requires the resolution of stable secondary structures and double-stranded RNA intermediates, meaning RNA remodeling is essential for efficient genome synthesis^37^. Although NS3 is widely proposed to support this process, its specific role as an ATP-dependent helicase remains unclear. Both NS3 and its picornaviral homolog 2C exhibit ATPase activity and bind RNA^38–40^, yet reports of their RNA unwinding capabilities have been inconsistent. Some studies have demonstrated ATP-dependent helicase activity, including bidirectional activity for NS3 and 3′ to 5′ activity for 2C^11,41^. Other studies failed to detect unwinding activity, reporting only an RNA-stimulated ATPase function or ATP-independent RNA chaperoning activity^12,38^.

Our soluble NS3 construct lacks the native N-terminal domain, which we replaced with a heterologous hexamerization motif to promote oligomerization. The native NTD is predicted to form a positively charged, membrane-embedded pore, and is likely critical for RNA engagement and directional translocation. Its absence may explain the lack of processive RNA unwinding observed in our assays. These findings may help reconcile prior evidence that full-length NS3 and eukaryotic expression systems are required for helicase activity^11,12^. This dependence may stem from a requirement for host-derived lipids to support correct NTD folding. While technically challenging, future efforts to dissect the proposed translocase activity of NS3 and possible helicase activity, should focus on the full-length, membrane-embedded protein.

Consistent with its proposed role in replication, NS3 has also been shown to colocalize with double-stranded RNA and NS7 at replication organelles^13,42^, further reinforcing its proximity to active sites of genome synthesis. Moreover, NS3 has been shown to stimulate the RNA synthesis activity of the viral polymerase NS7 in vitro, suggesting a direct functional interplay between these replication proteins^11^. Our single-molecule magnetic tweezer experiments and biochemical assays support a role for the NS3 cytosolic domains in binding ssRNA, consistent with a possible translocase function.

In addition to its proposed roles in replication, NS3 has been implicated in several other cellular processes, including the induction of apoptosis and the remodeling of host cell membranes^13–15^. Notably, our structural analysis reveals that the HBD of NS3 shares homology with a BAG-family co-chaperone^30^. BAG-domain proteins are known to regulate apoptosis and proteostasis by modulating the activity of HSP70 chaperones^43^. Prior studies have shown that the NS3 HBD is essential for virus-induced apoptosis^15^, raising the possibility that structural mimicry of BAG proteins may contribute to this activity. Supporting a possible role in chaperone modulation, both HSP70 and HSP90 have been implicated as pro-viral in the MNV replication cycle^44^.

With respect to membrane interactions, NS3 has also been shown to associate with various host membranes, including the endoplasmic reticulum, mitochondria and lipid-rich bodies, underscoring its multifunctional role during infection. Overexpression of NS3 induces the formation of cholesterol-rich, vesicle-like structures, suggesting a role in membrane remodeling^13,14,35^. These functions rely on the membrane-binding NTD. Our AlphaFold3-based model of this region, generated in the presence of fatty acids and functionally supported in a viral context, provides a structural framework to explain previous genetic findings. For example, the previously identified infectivity-enhancing K40R substitution lies within the interior of the predicted transmembrane pore^45^, while our charge-reversal K40E mutation abolishes recovery of infectious virus, independently supporting the functional importance of this pore-lining residue. Another recent study proposed that the NS3 NTD adopts an MLKL-like pore-forming fold. However, our attempts to reproduce this architecture across norovirus genogroups I–V using AlphaFold3 yielded low-confidence and inconsistent predictions (Supplementary Fig. 15G). While our high-confidence model strongly suggests an alternative architecture, this is compatible with the authors’ functional observations of lipid binding and membrane permeabilization through the formation of a size-selective pore^35^.

The alignment of the transmembrane domain, central helical bundle, and AAA+ motor domain forms a continuous conduit that may allow RNA to be threaded across replication organelle membranes. The AAA+ pore in our model exhibits a negative electrostatic potential, similar to single- and double-stranded translocation systems observed in other viruses^31–33^, which could facilitate RNA translocation by reducing nonspecific interactions. Our integrative structure of the full-length NS3 complex may have important functional implications. If NS3 indeed facilitates RNA translocation across membranes, it implies that genome synthesis may occur within a protected, membrane-bound compartment from which nascent RNA is exported for either translation or encapsidation (Fig. 7B). While such a luminal replication site has not yet been definitively identified, tomography studies of MNV and enterovirus-infected cells have identified single- and double-membrane compartments to accumulate in infected cells. Notably, the single-membrane structures observed in these studies could, in principle, support such a topological arrangement and have been proposed as the primary site of replication in the case of MNV and poliovirus^42,46–49^.

Based on the conserved polarity of substrate translocation by SF3 helicases, RNA is likely transported through the channel in a 3′ to 5′ direction. Our NS3 complex diverges from classical SF3 architectures by replacing the static oligomerization domain with a symmetry-matched spiral ring coordinated with the ATPase motor^19,50^. In contrast to the planar HBD tier seen in DNA helicases^16^, in NS3, the equivalent function is likely fulfilled by the transmembrane domain, which may act as a mechanical anchor during ATP-driven RNA translocation. A further hypothesis consistent with this architecture is that, if NS3 transports RNA across replication organelle membranes, the transport pathway would need to accommodate VPg, which is covalently linked to the 5′ end of the genome^36^. Consistent with our translocase model, a recent preprint describing a picornaviral 2C:ssRNA holoenzyme reported ssRNA threaded through the central pore and an analogous backbone-carbonyl contact with the RNA 2′-OH, supporting a conserved mode of RNA engagement by 2C-like ATPases^51^.

Our findings not only suggest a distinct model for norovirus replication but would also reconcile the strategies employed by picornaviruses with those of other positive-strand RNA viruses that remodel host membranes to spatially separate replication from downstream steps^52,53^. This raises questions as to how NS3 could coordinate the polarity, timing, and specificity of RNA export, and how this might be integrated with downstream capsid assembly^54^. Notably, genetic and biochemical studies have previously suggested direct interactions between the picornaviral 2C protein and capsid components^55^, supporting the idea that RNA replication and encapsidation are spatially and functionally coupled. Furthermore, in situ cryo-electron tomography of poliovirus-infected cells has revealed viral capsids connected to replication organelles by a proteinaceous complex of approximately 230 kDa^49^. Based on our model, it is reasonable to speculate that this complex may correspond to the 2C hexamer. It would be particularly interesting to perform similar in situ structural studies on MNV infected cells, which could help determine whether NS3 adopts an analogous complex on viral replication organelles.

Finally, our structural framework highlights NS3 as a compelling antiviral target. We and others have previously shown that the related 2C protein in enteroviruses can be inhibited by small molecules, such as S-fluoxetine, which binds to the predicted pore of the AAA+ domain and allosterically impairs its function^24,56^. Given the structural homology between 2C and NS3, including conservation of the central channel architecture, antiviral compounds could be developed to target norovirus NS3. If the export of newly synthesized genomic RNA is indeed essential for efficient replication, then inhibitors that block RNA translocation through this channel would be expected to disrupt genome synthesis and packaging. This could help explain why such compounds have previously emerged as inhibitors of these processes^56–58^. The high-resolution details of the NS3 ATP-binding pocket and central pore provided here offer a strong foundation for structure-guided antiviral discovery.

In summary, our cryo-EM structure of norovirus NS3, supported by an integrative full-length model, positions NS3 as a candidate membrane-spanning RNA translocase, a role distinct from its better characterized DNA virus counterparts^16^. In contrast to the large crown-like replication complexes formed by other positive-strand RNA viruses, such as coronaviruses, alphaviruses, and nodaviruses^53^, the compact hexameric architecture of NS3 suggests a streamlined mechanism for RNA export across replication organelle membranes. These findings raise the possibility that such minimal, membrane-anchored ATPases may represent an alternative strategy for spatially coupling genome synthesis and packaging, one that could be conserved among the order *Picornavirales*. While further studies will be required to confirm the functional implications of this architecture, our model lays a foundation for future mechanistic investigations and may inform the development of targeted antiviral strategies.

## Methods

### Cells and reagents

BHK-21 cells (obtained from ATCC) and BV-2 cells (gifted by Ian Goodfellow, University of Cambridge) for MNV experiments were maintained as described previously^59^. HEK293T cells were obtained from ATCC and maintained in Dulbecco’s Modified Eagle’s Medium (DMEM) supplemented with 10% (V/V) fetal bovine serum (FBS) and 100 µg/mL penicillin/streptomycin (P/S).

Adenosine 5′-O-(3-thiotriphosphate) (ATPγS) (Calbiochem [Sigma-Aldrich]) was purchased as a 100 mM solution in water. The cc-hex peptide GELKAIAQELKAIAKELKAIAWELKAIAQGAG was purchased from Tsingke and dissolved in DMSO to generate a 100 µM stock solution.

### Cloning and construct design

The codon sequence of human norovirus (HuNoV) GI (NC_001959.2) NS3 (amino acids 62–363) optimized for bacterial expression (GenScript) was cloned into the expression vector pET-28b using primer-facilitated ligation. At the N-terminus, a HRV 3C protease-cleavable sequence introduces a Strep-tag II, a His_6_-tag, and MBP-tag to facilitate expression and purification. For the hexameric construct, a 32-residue long codon-optimized cc-hex sequence^29^ followed the 3C protease cleavage sequence at the 5′ end of the NS3 coding sequence (Fig. 1A and Supplementary Fig. 1B). As a control protein, we used the MBP-hex construct without the NS3 sequence. Mutations were introduced via site-directed mutagenesis using Q5® High-Fidelity DNA polymerase (New England Biolabs). All oligonucleotides were purchased from IDT.

The plasmid, pT7-MNV*, containing the infectious clone sequence from the MNV-1 strain CW1P3^60^ under control of the T7 promoter was used for virus recovery. To exchange NS3 amino acids, standard two-step overlapping PCR mutagenesis was used with pT7-MNV* plasmid as template, exchanging an ApaI-to-XhoI fragment. The pcDNA3-based expression plasmids used for coupled in vitro transcription and translation assays expressing MNV ORF1 has been previously described^61^.

The sequences of all plasmids were verified by DNA Sanger sequencing (Macrogen). The sequences of the primers used in this study are provided in the Source Data file.

### Protein expression and purification

NS3 proteins were expressed in *E. coli* Rosetta 2 (DE3) (Sigma-Aldrich). Cells were grown in Terrific Broth (TB) medium containing kanamycin (50 µg/mL) and chloramphenicol (34 µg/mL) at 37 °C shaking at 180 rpm until an OD_600_ between 0.5 and 0.8 was reached. After induction of protein expression with 0.5 mM isopropyl β-D-thiogalactopyranoside (IPTG), cells were cultured at 18 °C overnight, shaking. Cells were harvested by centrifugation at 5000 × *g*, and the cell pellet was stored at −20 °C if the purification was resumed later. The pellet was resuspended in lysis buffer (50 mM Tris pH 8.0; 300 mM NaCl; 10 mM imidazole; 1 mM MgCl_2_; 0.1% Triton X-100; 5% glycerol; 0.25 mg/ml lysozyme; 10 µg/ml DNase I; and 1 tablet cOmplete EDTA-free protease inhibitor cocktail per 10 ml buffer) and the samples were sonicated on ice with 8–10 cycles of 30-s sonication and a 15-s pause (Sonics & Materials Inc.). The soluble phase was separated by centrifugation at 30,000 × *g* for 45 min at 4 °C. The 0.45 µm filtered supernatant was applied for immobilized metal affinity chromatography (IMAC) onto a HisTrap HP column (Cytiva) equilibrated with binding buffer (50 mM Tris pH 8.0, 300 mM NaCl, and 25 mM imidazole) using an Äkta go system (Cytiva) with the UNICORN software version 7.6 (Cytiva) at 4 °C and isocratically eluted with 500 mM imidazole.

To obtain cleaved constructs, the samples were incubated with HRV type 14 3C protease (Sigma-Aldrich) overnight at 4 °C, while dialyzing against the above-mentioned binding buffer containing 1 mM dithiothreitol (DTT). A second IMAC step captured the His-tagged 3C protease, the cleaved Strep-His-MBP-tag, and any uncleaved constructs, while the flow-through containing our protein of interest was further concentrated and applied for size exclusion chromatography, using a Superdex 200 Increase 10/300 GL column (Cytiva). The cleaved hexamers were eluted in cryo-EM buffer (25 mM Tris pH 8.0, 300 mM NaCl, and 1 mM MgCl_2_) and the cleaved monomers in storage buffer (10 mM HEPES pH 7.5, 150 mM NaCl, 10 mM MgCl_2_).

The uncleaved constructs were separated by SEC directly after IMAC, using a Superose 6 Increase 10/300 GL column (Cytiva) for hexameric constructs and a Superdex 200 Increase 10/300 GL column (Cytiva) for constructs lacking the cc-hex domain. The samples were eluted with storage buffer.

The purity of all protein samples was confirmed by SDS-PAGE, using denaturing 4× NuPAGE LDS sample buffer (Thermo Fisher Scientific). All concentrated protein samples were aliquoted, flash-frozen in liquid nitrogen, and stored at −80 °C until further analysis.

### NTPase assay

The release of inorganic phosphate during NS3-mediated hydrolysis of nucleotide triphosphates (NTPs) was measured using the Malachite Green Phosphate Assay Kit (Sigma-Aldrich) for end-point reaction evaluation, as previously reported with minor changes^24^. In brief, 250 nM enzyme in a final volume of 50 µL reactions in ATPase reaction buffer (10 mM HEPES pH 7.5, 25 mM NaCl, 10 mM MgCl_2_, and 1 mM DTT) was first incubated with the depicted ATPγS concentration for 30 min at room temperature (where applicable) before 1 mM NTP (Thermo Fisher Scientific for all except TTP from Sigma-Aldrich) was added to start the reaction. For experiments testing the effect of ssRNA on ATPase activity, a 50-nt poly(A) ssRNA substrate (Eurofins) was added at the indicated final concentrations immediately before initiation of the reaction with ATP. After incubation for 60 min at 37 °C, the reactions were diluted 20-fold with Milli-Q water (950 µL) to avoid precipitation downstream during the malachite green reaction.  Eighty microliters of diluted samples were added to 20 µL of AB reagent in clear flat-bottom 96-well plates (Corning 3599). After color development of 30 min at room temperature, the absorbances were read at 630 nm on a microplate reader (FLUOstar Omega). A standard curve allowed quantification of the samples.

### Mass photometry

Mass photometry measurements were carried out on a OneMP Mass Photometer (Refeyn Ltd). Glass microscope coverslips (24 mm × 50 mm, Paul Marienfeld GmbH) were sonicated for 10 min in isopropanol (HPLC grade), then Milli-Q water, repeating three times, before they were dried using a clean N_2_ stream and a clean CultureWell gasket (Grace Biolabs) was applied. Mass calibration was done using an in-house mix of IgG halfbodies (73 kDa), IgG1 (149 kDa), apoferritin (513 kDa), and IgM (967 kDa). Protein dilutions were carried out immediately before measurements. For the experiments, 12 μL sample buffer was loaded into the well to find focus and 3 μL of purified protein sample was added for measurements. The final concentrations of the samples ranged approximately from 10 to 50 nM. Movies were recorded for 60 s using AcquireMP v2023 R1.1 (Refeyn Ltd) and analysed using DiscoverMP v2023 (Refeyn Ltd). The calibration measurement was used to generate a calibration curve relating ratiometric contrast to molecular mass, which was applied to both the ΔN61-NS3 and hex-ΔN61-NS3 measurements. Figure 1C was generated by combining the two measurements in the DiscoverMP figure builder as a 2D histogram.

### Native mass spectrometry

Native MS experiments were carried out on a Thermo Scientific Exactive Plus EMR mass spectrometer with a static spray set-up, using in-house produced gold-coated electrospray borosilicate capillaries. Purified cleaved NS3 and hex-NS3 constructs were buffer-exchanged six times into 150 mM ammonium acetate solution (pH 7.0) using Amicon Ultra 0.5 mL 10 kDa centrifugal filters (Merck Millipore). The concentration was calculated using the A280 extinction measured on a Nanodrop One system (Thermo Fisher Scientific) and the theoretical extinction coefficient based on the sequence of the construct in the unreduced form^62^. These were 17,335 M^−1^ cm^−1^ for cleaved NS3 and 24,325 M^−1^ cm^−1^ for cleaved hex-NS3. Samples were diluted for native MS using the same ammonium acetate solution. Cleaved NS3 was diluted to 10 μM, and cleaved hex-NS3 was diluted to 25 μM. Spectra were analysed and deconvoluted using UniDec version 6.0.4. For each construct, one buffer-exchanged sample was diluted and measured (*n* = 1); no separate control was applicable. The detected mass range was set from 1000 *m/z* to 12,000 *m/z* with an injection time of 200 ms. Ion transfer optics (injection flatapole, inter-flatapole lens, bent flatapole and transfer multipole) were set to 8, 7, 6, and 7 V, respectively. Additional desolvation was achieved by setting the HCD energy to 80 V. Resolution was set to 8750, equivalent to a transient time of 32 ms. Spectra were inspected in Thermo FreeStyle, and a summed average spectrum over each measurement was exported as an xy list and imported into UniDec v6.0.4 for deconvolution. Detailed instrument metadata are retained in the deposited Thermo RAW files, and detailed UniDec configuration parameters are provided in the deposited native MS dataset.

### Negative-stain electron microscopy

Cleaved hex-NS3 was diluted to 0.03 mg/ml in ATPase reaction buffer containing 1 mM ATPγS, on ice. Subsequently, 3 μl of the sample was applied to carbon-coated copper grids that had been glow-discharged for 30 s using a Cressington 208 instrument. The sample was allowed to absorb for 30 s prior to two washes with Milli-Q water and staining with 2% uranyl acetate solution. Grids were allowed to dry in air for ∼5 min and then imaged on a Thermo Scientific Talos L120C transmission electron microscope (TEM) operated at 120 keV, equipped with a CCD camera.

### Sample preparation for cryo-EM

Cleaved hex-NS3 was diluted to 0.35 mg/ml in ATPase reaction buffer containing 1 mM ATPγS, on ice. Approximately 3 µl of the complex was applied to QUANTIFOIL 200 mesh R1.2/1.3 copper grids overlaid with 2 nm layer of carbon that had been glow-discharged for 30 s on a GloQube (Quorum) at 20 mW power. The sample was applied at 4 °C and 95% relative humidity inside a Vitrobot Mark IV (Thermo Fisher Scientific). The grids were then blotted for 7 s with +2 blot force and plunge-frozen in liquid ethane.

### Cryo-EM data acquisition

A single grid was imaged on a Thermo Scientific Glacios Cryo-TEM equipped with a Falcon 4i direct electron detector and Selectris energy filter using EPU 2 acquisition software. A total of 1010 movies were collected at a nominal magnification of ×130,000 in EER format at 0.9 Å per pixel with a total dose of 50.5 electrons per Å^2^. Defocus targets cycled from −0.75 to −1.5 μm. A summary of all data collection parameters is shown in Supplementary Table 1.

### Single-particle image processing

Patch motion correction and patch CTF estimation were carried out in cryoSPARC^63^. Templates for particle picking were generated from a 20 Å resolution map generated from the NS3 AlphaFold2 coordinates using the UCSF Chimera molmap command^64,65^. Template-based particle picking was performed with a particle diameter of 110 Å. A total of 1,229,229 picked particles were subsequently extracted in a 76-pixel box. During extraction, particles were Fourier binned by a non-integer value, resulting in a final pixel size of 3.32 Å. A single round of two-dimensional (2D) classification was carried out, after which a subset of 2D classes containing 235,173 particles, corresponding to top and side views of the NS3 complex, were selected. Ab initio reconstruction with four classes generated one well-defined reconstruction of the NS3 hexamer and three junk classes. The resulting ab initio maps were then used as initial models for iterative rounds of heterogeneous refinement, where the particles belonging to the NS3 class were used as the input for subsequent rounds. To avoid missing NS3 particles that may have been removed during initial stringent selection of 2D classes, heterogeneous refinement was carried out on the initial particle stack of 1,229,229 particles. Five rounds of heterogeneous refinement yielded a well-defined reconstruction of the NS3 complex containing 123,643 particles. These were then re-extracted unbinned in a 300-pixel box, resulting in a final pixel size of 0.9 Å. Subsequently, non-uniform refinement was carried out on the extracted particles with C1 symmetry imposed, yielding a reconstruction with a global resolution of 2.9 Å. The gold standard Fourier shell correlation (FSC) criterion (FSC = 0.143) was used for calculating all resolution estimates, and 3D-FSC plots were generated in the 3DFSC Processing Server^66^. To facilitate model building, the globally refined map was filtered by local resolution in cryoSPARC. An overview of the data processing pipeline is shown in Supplementary Fig. 5.

### Model building and refinement

An AlphaFold2 model was used as a starting point for modeling the NS3 hexamer^65^. First, the predicted hexameric model (residues 62–363) was fitted into the EM density map using the UCSF Chimera Fit in Map tool^64^. Next, molecular dynamics flexible fitting in Namdinator was employed to account for differences in the quaternary structure of the predicted model and the experimental map^67^. Water molecules, magnesium ions, and ATPγS molecules were added in Coot^68^. Subsequently, iterative rounds of modeling in ISOLDE and refinement in Phenix were carried out to improve non-ideal rotamers, bond angles, and Ramachandran outliers^69,70^. During real-space refinement, secondary structure restraints were imposed. Phenix ReadySet was used to generate restraints for coordinated Mg^2+^ ions. Model validation was carried out using MolProbity^70,71^. All data collection, image processing, and refinement information can be found in Supplementary Table 1.

### Protein structure prediction

AlphaFold2 predictions were performed using the COSMIC² server^65,72^. AlphaFold3 predictions were performed using the AlphaFold Server with default settings. For nucleic-acid-bound models, the ΔN61-NS3 hexamer was predicted in the presence of ATP, Mg²⁺ and either a 50-nt poly(A) ssRNA or a 50-nt poly(dA) ssDNA substrate. The five ranked models from each prediction were inspected for nucleic acid placement within the central pore. Full-length NS3 predictions were performed using representative norovirus genogroup I–V sequences retrieved from NCBI: GI (NC_001959.2), GII (AXQ39939.1), GIII (PV053516.1), GIV (YP_009237903.1), and GV (YP_724457.1). Hexameric assemblies were predicted in the absence or presence of 50 oleic acid molecules. For the genogroup comparison and subunit-number analyses, predictions were performed with poly(A) RNA, ATP, Mg²⁺, and oleic acid molecules included as input components. Additional predictions were performed for assemblies containing one to ten NS3 subunits, and interface confidence was assessed using iPTM. Additional monomeric NS3 truncation constructs were predicted using genogroup-specific domain boundaries corresponding to the previously proposed MLKL-like region, including removal of the N-terminal residues preceding the putative MLKL-like segment and truncation after its C-terminal boundary.

### Analysis and visualization

Protein–protein and protein–ligand interactions were analyzed using PDBePISA and LigPlot+^73,74^. The volume of the NS3 HBD chamber was calculated using CASTpFold^75^. Structural homology analysis was performed in Foldseek and DALI^76,77^, and structural phylogenetic analysis was carried out using FoldTree^78^. Figures were generated using UCSF ChimeraX^79^. Structural biology applications used in this project were compiled and configured by SBGrid^80,81^.

### MNV in vitro transcription and virus recovery

MNV plasmids were linearized with *NotI* and extracted before being used for in vitro transcription using the HiScribe™ T7 ARCA mRNA Kit (New England Biolabs) or mMESSAGE mMACHINE™ T7 Transcription Kit (Thermo Fisher Scientific), following the manufacturer’s instructions^59^. RNA was purified and concentrated using the RNA Clean and Concentrator Kit (Zymo) or the NucleoSpin RNA kit (Macherey-Nagel). RNA transfection was carried out as previously described^59^. For mutational analysis of the NS3 NTD, mutations were introduced into the recently described HiBiT-tagged MNV infectious clone^82^. Briefly, HEK293T cells were seeded in 6-well plates at 3 × 10^5^ cells per well and transfected the following day with 1 µg of in vitro-transcribed RNA per well. At 3 days post-transfection, cells were lysed by three freeze-thaw cycles, and lysates were clarified by centrifugation at 5000 × *g* to generate MNV stocks. Viral titers were determined by TCID_50_ assay.

### TCID_50_ assay

Viral infectivity was determined using a TCID_50_ assay as described previously^83^. Briefly, BV-2 cells were seeded into 96-well plates at 2 × 10^4^ cells/well and left overnight before infection. TCID_50_ values were calculated according to Spearman and Kärber.

### Preparation of RNA duplex substrate

The 3′ overhang dsRNA substrate was generated by annealing two complementary, commercially synthesized RNA oligonucleotides (GENEWIZ, Germany): a 5′ HEX-labeled strand (RNA1: 5′-CAUUAUCGGAUAGUGGAACCUAGCUUCGACUAUCGGAUAAUC-3′) and an unlabeled complementary strand (RNA2: 5′-GCUAGGUUCCACUAUCCGAUAAUG-3′). Oligonucleotides were mixed at equimolar concentrations in a final volume of 20 µL, heated to 95 °C for 5 min, and slowly cooled to room temperature to allow duplex formation.

### RNA duplex unwinding assay

RNA unwinding activity was assessed using a modified version of a previously described helicase assay^84^. Briefly, 20 µM recombinant proteins were incubated with 10 nM fluorescently labelled dsRNA substrate in a buffer containing 25 mM HEPES (pH 8.0), 30 mM NaCl, 5 mM MgCl₂, 2 mM ATP, 2 mM DTT, and 20 U RNasin ribonuclease inhibitor (Promega). DHX9 (BPS Bioscience) was used as a positive control. Reactions were carried out at 37 °C for 1 h and subsequently treated with 1 U Proteinase K to remove protein–RNA interactions. Reactions were terminated by the addition of 2× RNA loading dye (NEB) and analysed by native gel electrophoresis. Samples were resolved on a 15% native polyacrylamide gel and imaged using a Typhoon 9500 imager (GE Healthcare).

### Electrophoretic mobility shift assay

Recombinant proteins at 20 µM were incubated with 0.2 nM 5′-HEX labelled RNA1 (CAUUAUCGGAUAGUGGAACCUAGCUUCGACUAUCGGAUAAUC; GENEWIZ, Germany) in 25 mM HEPES (pH 8.0), 30 mM NaCl, 5 mM MgCl_2_, 2 mM DTT, 20 U RNasin Ribonuclease Inhibitor (Promega) and 5% glycerol. The reaction was incubated at 37 °C for 1 h and terminated by adding 2× RNA loading dye (NEB). Reactions were resolved by 6% native polyacrylamide gel electrophoresis, followed by imaging with a Typhoon 9500 imager (GE Healthcare).

### Statistical analysis

Statistical analysis and data visualization were performed using GraphPad Prism version 10. No inferential statistical tests were performed. Where shown, error bars represent SEM from independent biological replicates, as defined in the figure legends. Error bars were not derived from technical duplicates or from *n* < 3.

### Hairpin fabrication for helicase activity and refolding assay

The helicase hairpin was constructed following a protocol similar to that used for the RNA hairpin described previously^85^. The key difference lies in the orientation of the docking site: for helicase activity, the docking site was positioned on the 5′-end handle, opposite to the 3′-end docking used for polymerase activity, reflecting the opposite translocation polarities of the helicase and polymerase. The helicase docking site was ~20 nucleotides long, compared to 25 nucleotides for the polymerase. The loop oligonucleotide in the helicase hairpin was 4 nucleotides long, versus 20 nucleotides in the polymerase construct. The fabrication of the DNA hairpins was performed as described previously^86^.

### Flow cell assembly and surface functionalization

Flow cells were assembled as described previously^87^. A double layer of Parafilm was sandwiched between two #1 glass coverslips. The top coverslip had two holes for fluid access, while the bottom was coated with 0.1% (w/v) nitrocellulose in amyl acetate. The assembled cell was mounted in a custom holder and flushed with ~1 mL of phosphate-buffered saline (PBS). Polystyrene beads (3 µm diameter; LB30, Sigma-Aldrich), diluted 1:1000 in PBS, were incubated in the chamber for 3 min to allow non-specific adsorption to the surface. After rinsing with PBS, 50 µL of anti-digoxigenin (50 µg/mL in PBS) was introduced and incubated for 30 min. Excess antibody was removed with 1 mL of high-salt TE buffer (10 mM Tris, 1 mM EDTA, pH 8.0, 750 mM NaCl, 2 mM sodium azide), followed by 0.5 mL of standard TE buffer (150 mM NaCl). Finally, the surface was passivated with 10 mg/mL bovine serum albumin (BSA) in PBS with 50% glycerol for 30 min and rinsed with TE buffer.

### Single-molecule helicase unwinding experiments

Streptavidin-coated Dynabeads M-270 (20 µL; Thermo Fisher Scientific) were mixed with ~0.1 ng of RNA hairpin (in 40 µL total volume) and incubated for 5 min. Beads were rinsed with ~2 mL of TE buffer to remove excess RNA and unbound beads. Functional hairpins were identified based on the characteristic extension jump (~0.6 µm at 30 pN) observed during force ramping, indicative of hairpin opening.

The flow cell was flushed with 0.5 mL reaction buffer (20 mM HEPES, pH 7.0, 25 mM NaCl, 0.5 mM MgCl₂, 1 mM TCEP), followed by 100 µL of a reaction mix containing 2.3 µM cleaved hex-NS3 and 2 mM ATP. Hairpins were held closed at a constant force of 18 pN. Prior to measurements, hairpins were opened and closed twice to ensure tether integrity. The magnet was then fixed at a height corresponding to 18 pN for 20 min while helicase activity was monitored. Data were acquired at 58 Hz, with temperature maintained at 25 °C. Mechanical drift correction was performed by subtracting the position of a reference bead from the magnetic bead position, and the focus of the objective was fixed in respect of the flow cell surface^88^.

### Single-molecule hairpin refolding assay

Single-molecule hairpin refolding experiments were performed using the same RNA and DNA hairpin tether preparation procedure described above. Following identification of functional hairpin tethers, the flow cell was flushed with 0.5 mL reaction buffer containing 20 mM HEPES, pH 7.0, 25 mM NaCl, 0.5 mM MgCl₂, and 1 mM TCEP. Subsequently, 100 µL of the reaction mixture was introduced into the flow cell. For RNA hairpin experiments, reaction mixtures contained either 2 mM ATP with or without 5 µM NS3, 2 mM ATP with or without 1 µM hex-NS3, or no ATP with or without 1 µM hex-peptide. For DNA hairpin experiments, reaction mixtures contained no ATP and were supplemented with either 1 µM hex-NS3 or 1 µM hex-peptide, together with their corresponding protein-free control conditions. Hairpin refolding was monitored using dynamic force-ramp cycles in a magnetic tweezers setup. Force was increased from 4 to 35 pN and subsequently decreased back to 4 pN at a magnet speed of 0.1 mm/s. Two consecutive force-ramp cycles were performed for each tether. All measurements were carried out at 25 °C. Data were acquired at 58 Hz. Mechanical drift was corrected by subtracting the position of a reference bead from the magnetic bead position, and the objective focus was maintained at a fixed position relative to the flow-cell surface throughout the experiment.

### Calculation of hairpin refolding rates

DNA and RNA hairpin refolding was measured in the presence of 1 µM hex-NS3 and in the absence of ATP. Refolding events were identified from extension-time traces, and for each event, the refolding rate was calculated as the extension change divided by the event duration.

$$Refolding\,{rate}=\,\frac{\Delta {extension}}{\Delta {time}}$$

Here, extension change is in µm and time is in seconds, giving rates in µm/s. The mean refolding rate for each condition was then calculated from bead-averaged rates, and error bars represent the standard error of the mean (SEM).

### Reporting summary

Further information on research design is available in the Nature Portfolio Reporting Summary linked to this article.

## Supplementary information

Supplementary Information

Description of Additional Supplementary Files

Supplementary Movie 1

Reporting Summary

Transparent Peer Review File

## Source data

Source Data

## Data Availability

Atomic coordinates have been deposited in the Protein Data Bank under accession code 9R34. The corresponding EM density maps, including the final unsharpened, sharpened, local resolution-filtered maps, half maps, and mask, have been deposited in the Electron Microscopy Data Bank under accession code EMD-53547. Unaligned gain-normalized movies, motion-corrected micrographs, and the final refined particle stacks have been deposited in the Electron Microscopy Public Image Archive under accession code EMPIAR-12758. The integrative model of the full-length NS3 hexamer, AlphaFold prediction outputs, and raw and processed native mass spectrometry and mass photometry data used in this study are available through Zenodo entry 15556769. The magnetic tweezers data generated in this study are available through the Yoda data repository [https://doi.org/10.48338/VU01-U0QASY]. Requests for reagents should be directed to the corresponding author, D.L.H. Source data are provided with this paper.
