# Supplementary material for: Integrative structure of norovirus NS3 suggests a role in RNA transport
Source: Nat Commun. 2026 Sep 21;17:10991. doi: 10.1038/s41467-026-77946-7 (PMC13683342; doi:10.1038/s41467-026-77946-7)
Supplement: Supplementary file 1 [file 41467_2026_77946_MOESM1_ESM.pdf]

Supplementary Materials for

Integrative structure of norovirus NS3 suggests a role in RNA transport

Meryl Haas, Tino Hoeksma, Jake T. Mills, Cynthia Kelley, Arnab Das, Kankipati Teja Shyam, Robin Veenstra, Owen Byford, Henrietta Hof, Coco Niemel, Tim Donselaar, Ieva Drulyte, Frank J.M. van Kuppeveld, David Dulin, Joost Snijder, Morgan R. Herod, and Daniel L. Hurdiss

Correspondence to: d.l.hurdiss@uu.nl

This PDF file includes:

Supplementary Figs. 1 to 16

Supplementary Table 1

A

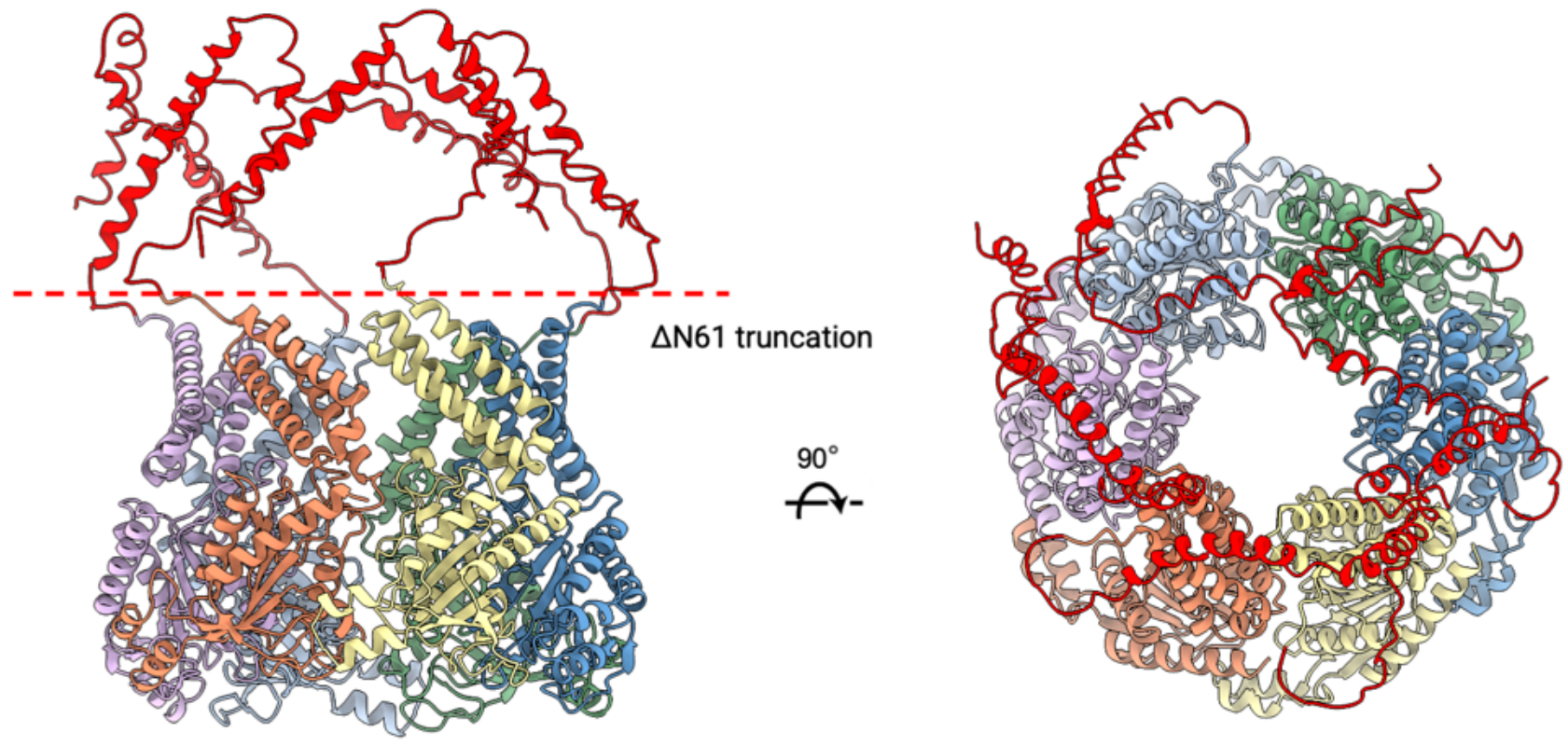

B

Strep-tag II | 6xHis

H_3N^+-MGSSWSHPQFEKSAHHHHHHMKIEEGKLVIWINGDKGYNGLAEVGKKFEKDTGIKVTVEH

Matlose-binding protein

PDKLEEKFPQVAATGDGPDIIFWAHDRFGGYAQSGLLAEITPDKAFQDKLYPFTWDAVRY

NGKLIAYPIAVEALSLIYNKDLLPNPPKTWEEIPALDKELKAKGKSALMFNLQEPYFTWP

LIAADGGYAFKYENGKYDIKDVGVDNAGAKAGLTFLVDLIKNKHMNADTDYSIAEAAFNK

GETAMTINGPWAWSNIDTSKVNYGVTVLPTFKGQPSKPFVGVLSAGINAASPNKELAKEF

LENYLLTDEGLEAVNKDKPLGAVALKSYEEELAKDPRIAATMENAQKGEIMPNIPQMSAF

Linker | HRV-3C | Linker

WYAVRTAVINAASGRQTVDEALKDAQTGGGGSLEVLFQGPGGGGSGGGGSGELKAIAQEL

cc-hex | Shi linker

KAIAKELKAIAWELKAIAQGAGGSGSYFQSNAGGDQTEKTLKDIESAVIDMEVLSSTSVT

huNoV GI NS3 (62-363)

QLVRDKQSARAYMAILDNEEEKARKLSVRNADPHVVSSTNALISRISMARAALAKAQAEM

TSRMRPVVIMMCGPPGIGKTKAAEHLAKRLANEIRPGGKVGLVPREAVDHWDGYHGEEVM

LWDDYGMTKIQEDCNKLQAIADSAPLTLNCDRIENKGMQFVSDAIVITTNAPGPAPVDFV

NLGPVCRRVDFLVYCTAPEVEHTRKVSPGDTTALKDCFKPDFSHLKMELAPQGGFDNQGN

TPFGKGVMKPTTINRLLIQAVALTMERQDEFQLQ-COO^-

Supplementary Fig. 1: Construct design for hex-NS3. (A) Two orthogonal views of the AlphaFold2[1] prediction of the full-length NS3 hexamer with the individual protomers shown in different colors. The ΔN61 truncated N-terminus is depicted in red. (B) Amino acid sequence of the MBP-hex-NS3 construct, including a heterologous hexamerization domain and linker described previously[2–4]. A human Rhinovirus (HRV) type-14 3C cleavage site separates the StrepII-, His-, and MBP-tag N-terminally from the cc-hex and ΔN61 truncated NS3 of HuNoV GI (NCBI Reference Sequence: NP_056820.1)

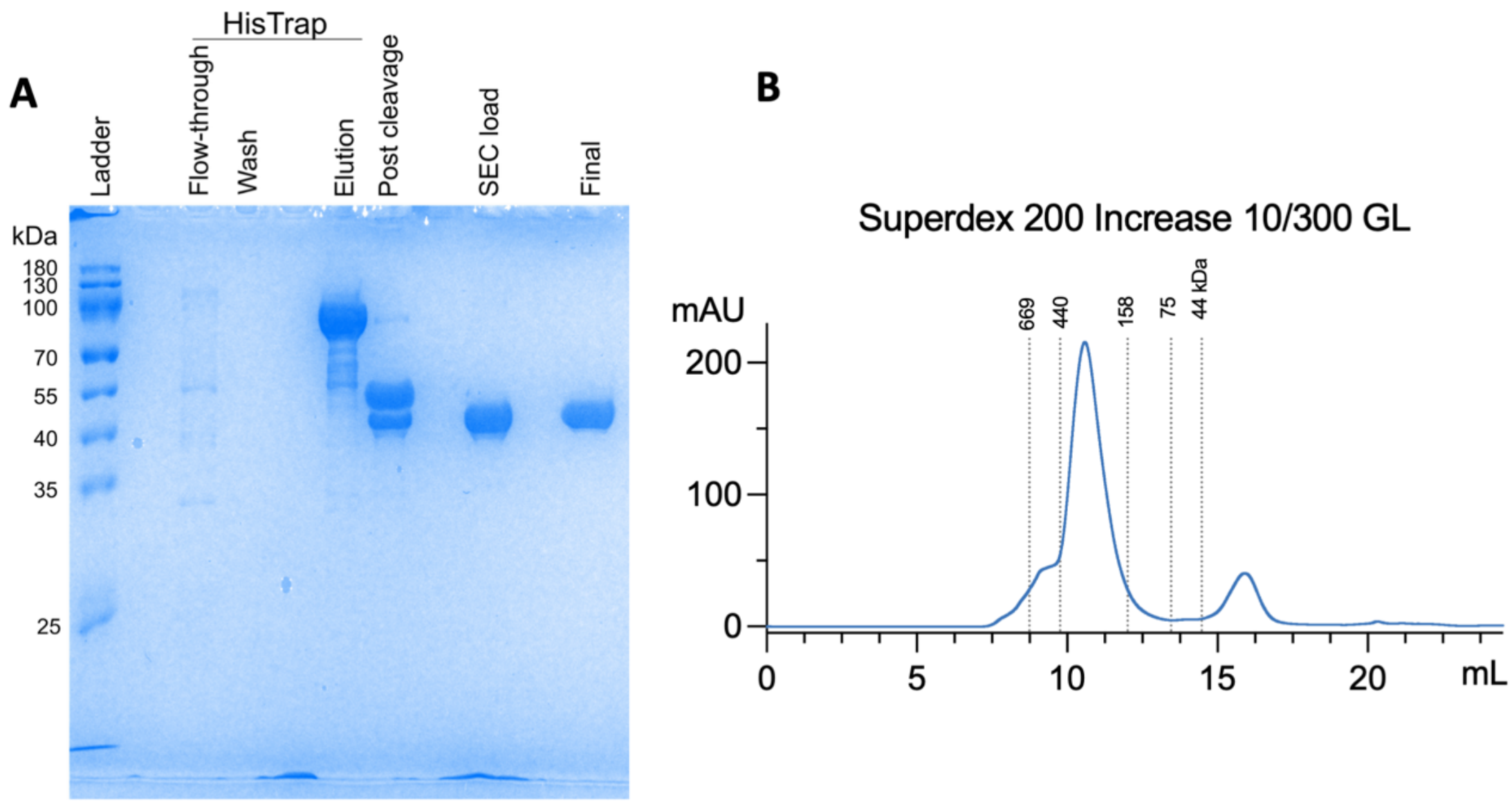

Supplementary Fig. 2: Purification overview for hex-NS3. (A) Samples from the individual purification steps are visualized on an SDS-PAGE gel. (B) Size-exclusion profile of the cleaved hex-NS3 on a Superdex 200 Increase 10/300 GL column (Cytiva). Calibration markers are indicated with dotted lines and corresponding masses. mAU, milliAbsorbance Unit at 280nm.

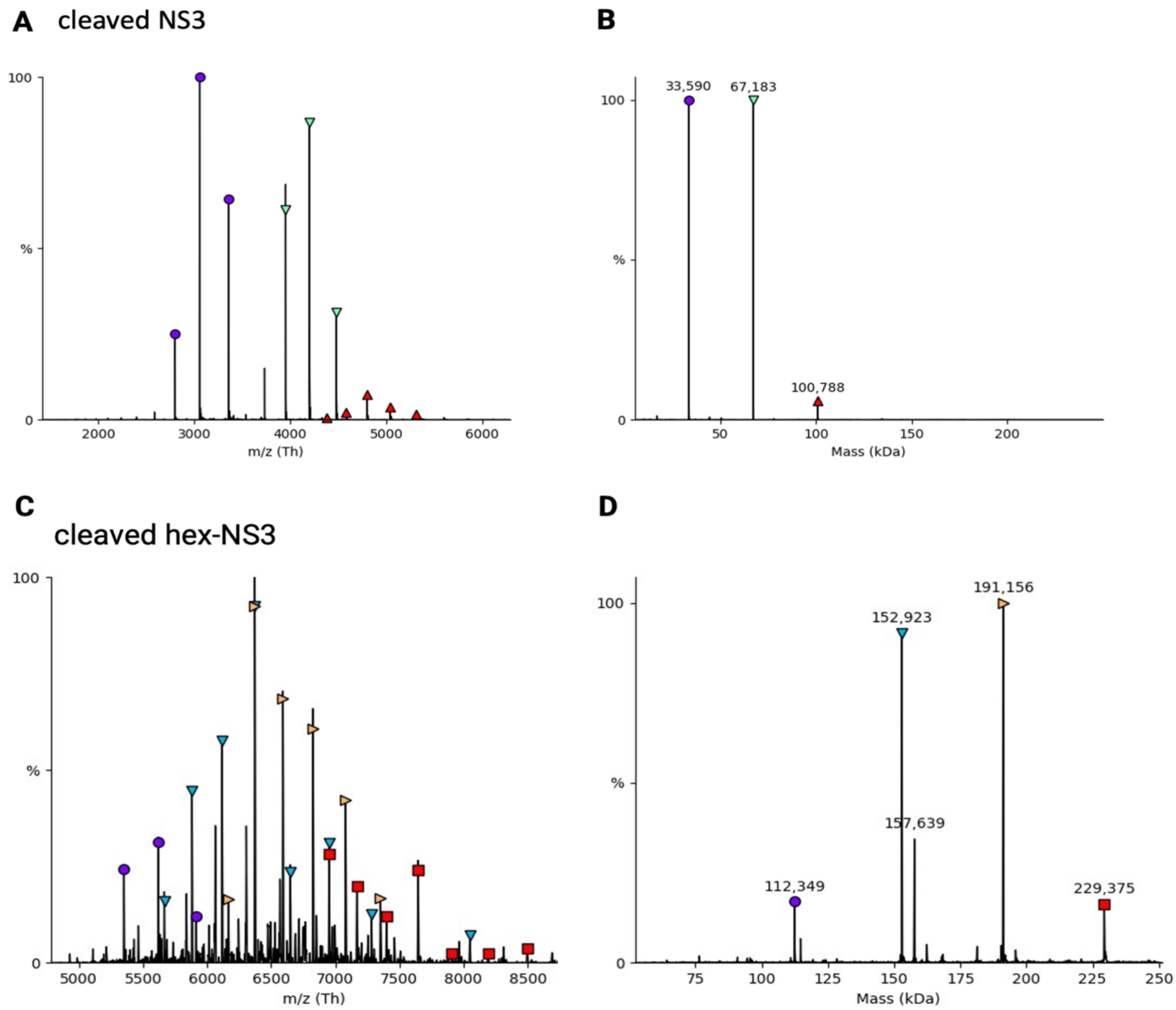

Supplementary Fig. 3: Native mass spectrometry analysis of cleaved NS3 constructs. (A) Native MS spectrum of cleaved NS3 measured at 10 µM and (B) the corresponding deconvoluted spectrum. (C) Native MS spectrum of cleaved hex-NS3 measured at 25 µM, based on the protomer concentration, and (D) the corresponding deconvoluted spectrum. One independent measurement was performed for each construct (n = 1).

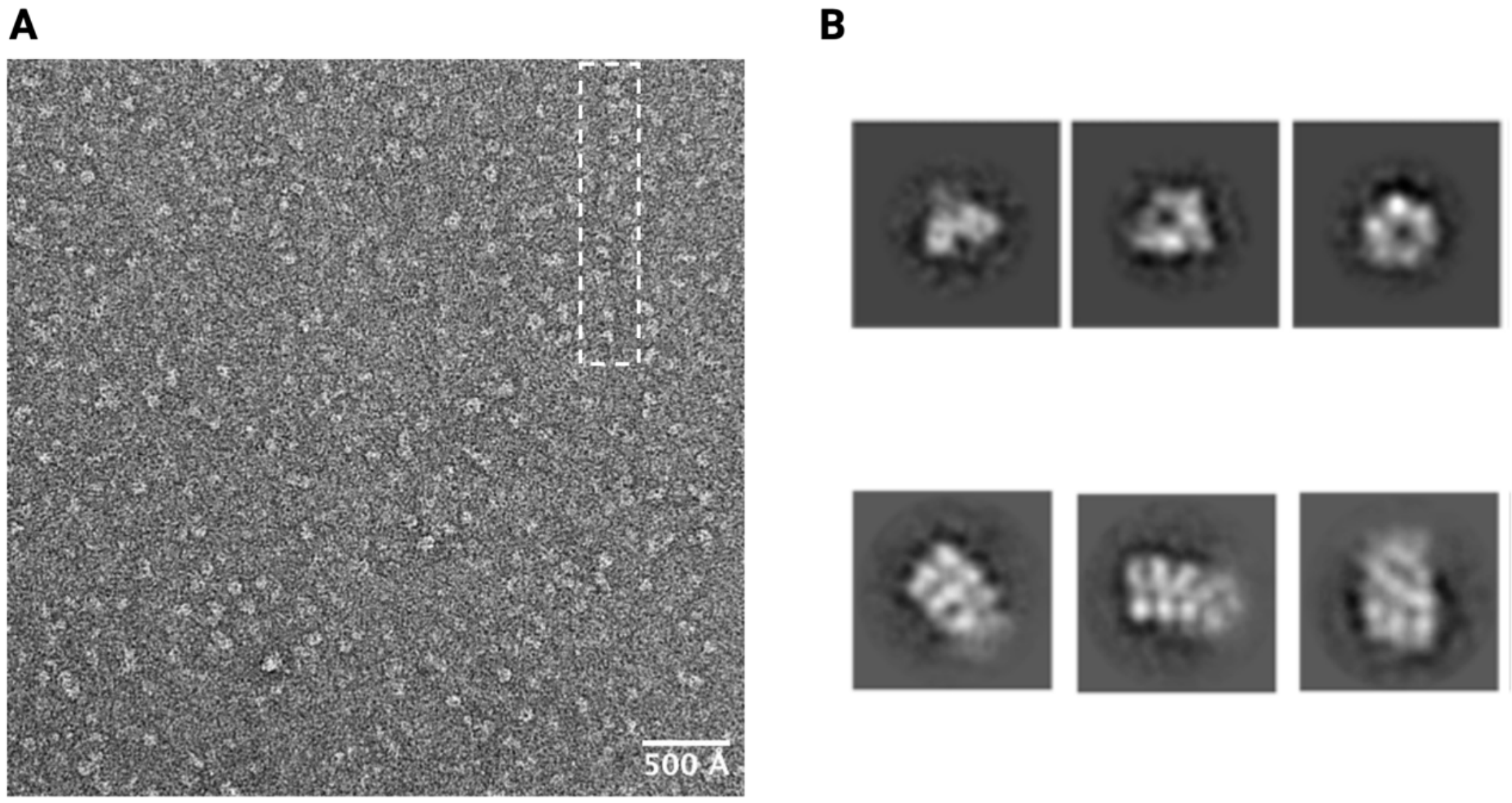

Supplementary Fig. 4: Negative-stain electron microscopy analysis of hex-NS3. (A) A representative micrograph of negative-stained cleaved hex-NS3 incubated with ATPγS. The dashed box indicates an example of observed filamentous structures. (B) Representative 2D class averages of NS3 hexamers and putative NS3 filaments.

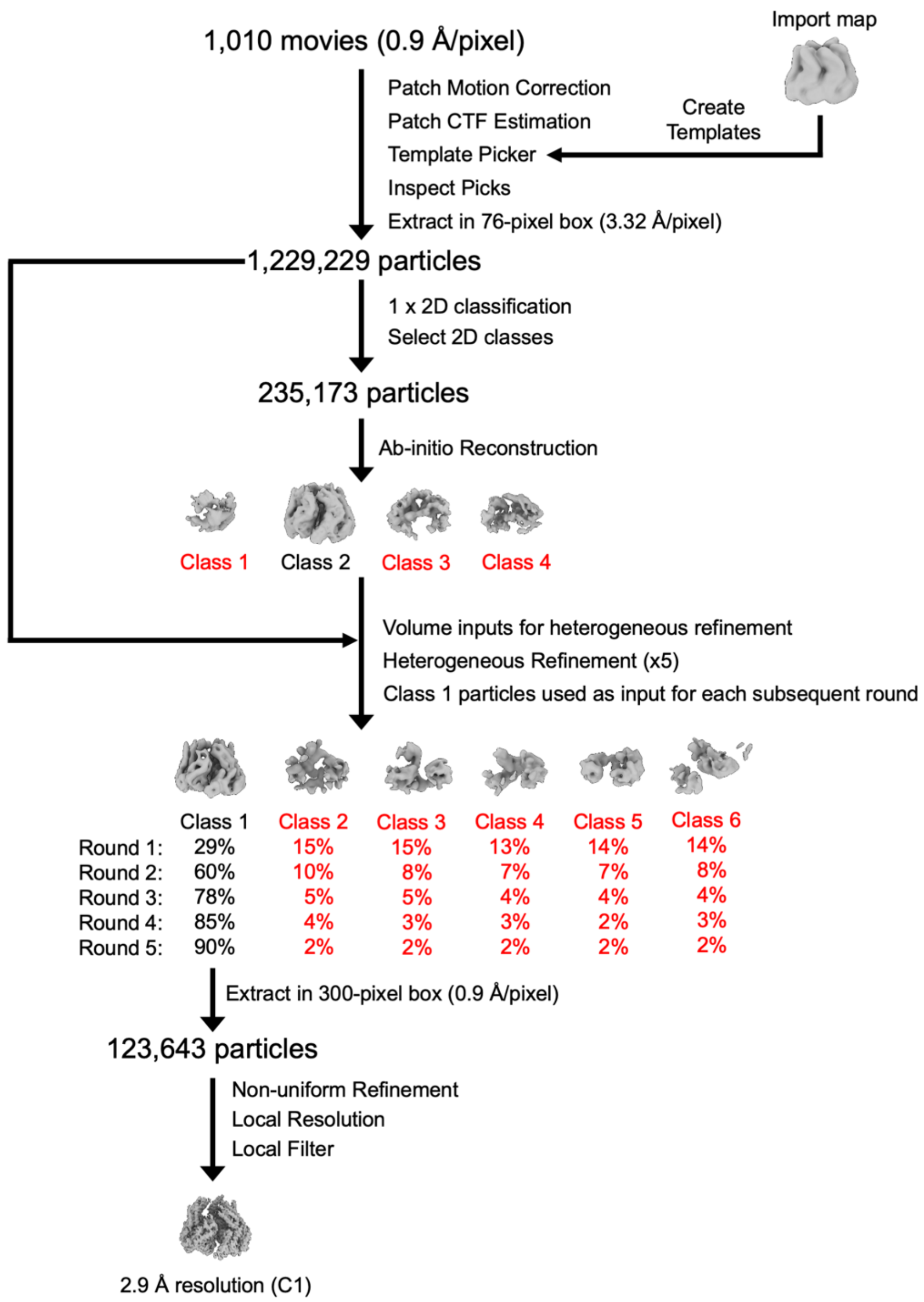

Supplementary Fig. 5: Single-particle cryo-EM data processing pipeline for hex-NS3 in complex with ATPγS. Schematic representation of the single-particle cryo-EM data processing pipeline for the hex-NS3 complex. The final reconstruction yielded a 2.9 Å resolution map. Additional details are described in the single-particle image processing section of the materials and methods. CTF: contrast transfer function.

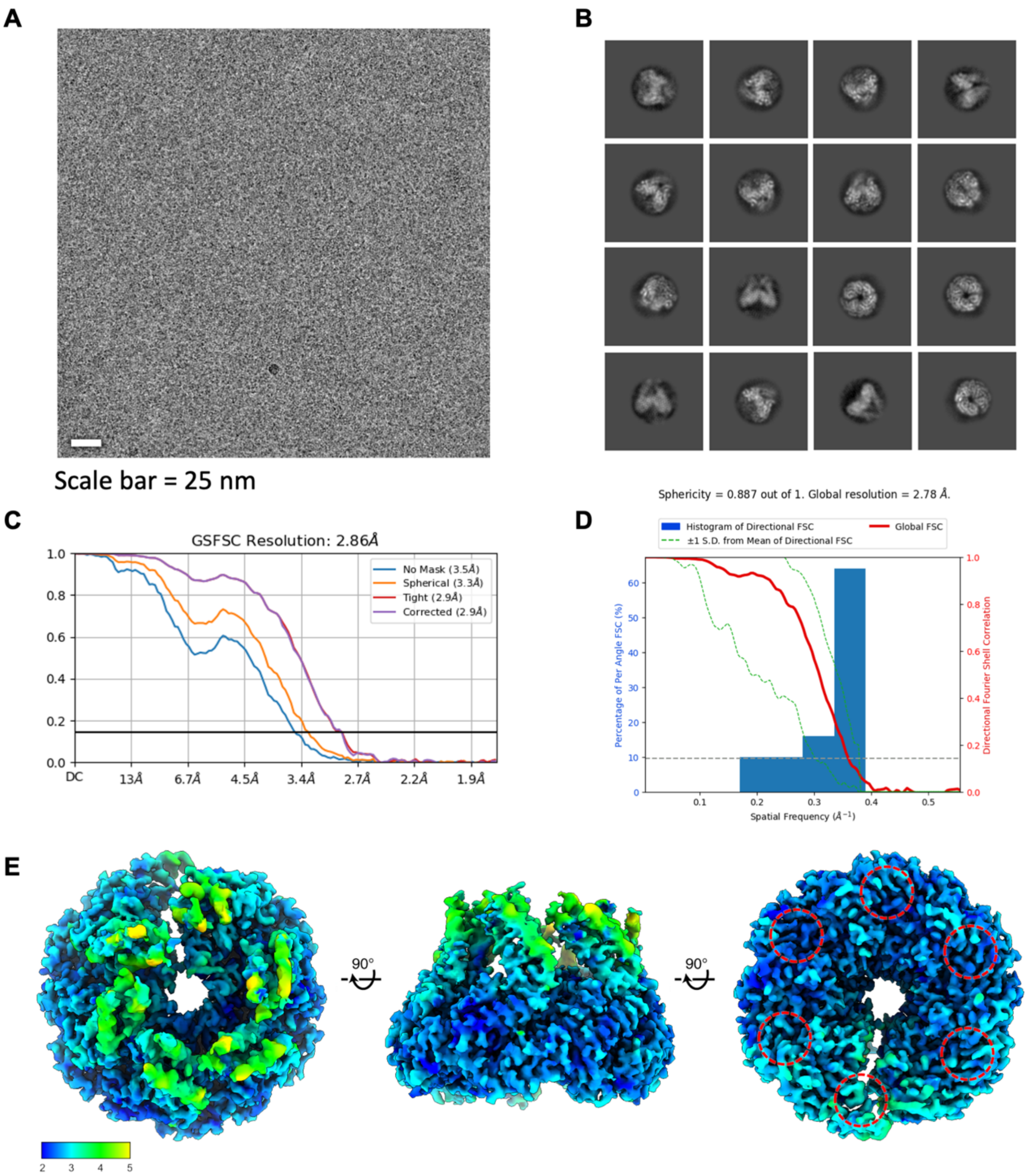

Supplementary Fig. 6: Single-particle cryo-EM data processing for the hex-NS3 in complex with ATPγS. (A) Representative motion-corrected electron micrograph of hex-NS3 in complex with ATPγS embedded in vitreous ice. Scale bar = 25 nm. (B) Representative reference-free 2D class averages. (C) Gold-standard Fourier shell correlation (FSC) curve generated from the independent half maps contributing to the 2.9 Å global resolution density map. (D) 3DFSC plot for the hex-NS3 complex[5]. (E) Three orthogonal views of the local resolution filtered EM density map for the refined hex-NS3 complex colored according to local resolution which was calculated in CryoSPARC[6]. The locations of the ATPγS binding sites are circled.

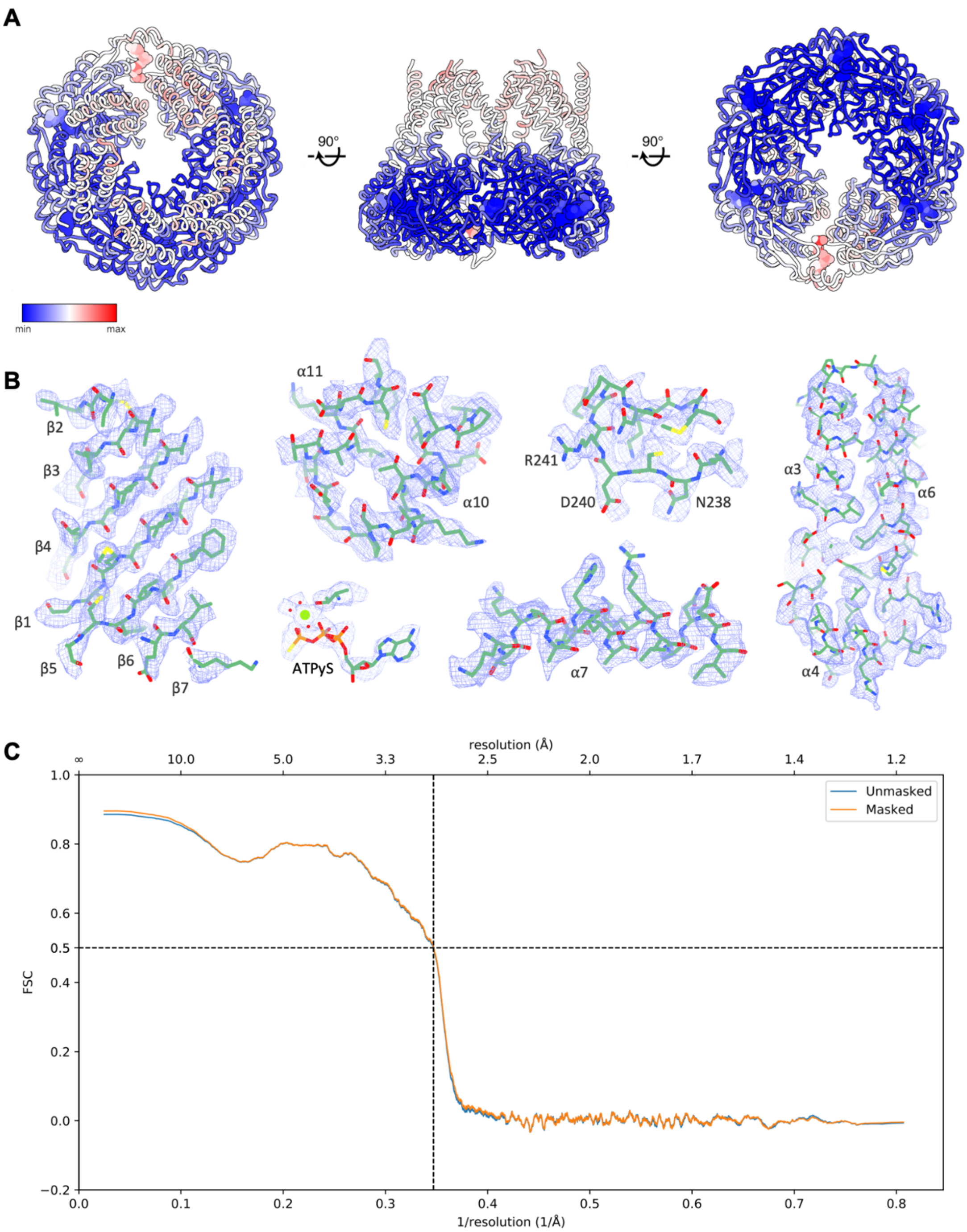

Supplementary Fig. 7: Atomic modelling of hex-NS3 in complex with ATPγS. (A) Atomic model of NS3 complex shown as three orthogonal views, with residues colored according to calculated B-factor. (B) Representative EM density and fitted atomic model for chain C of the hex-NS3 complex. (C) Map versus model FSC curves for the final refined model, generated in Phenix[7].

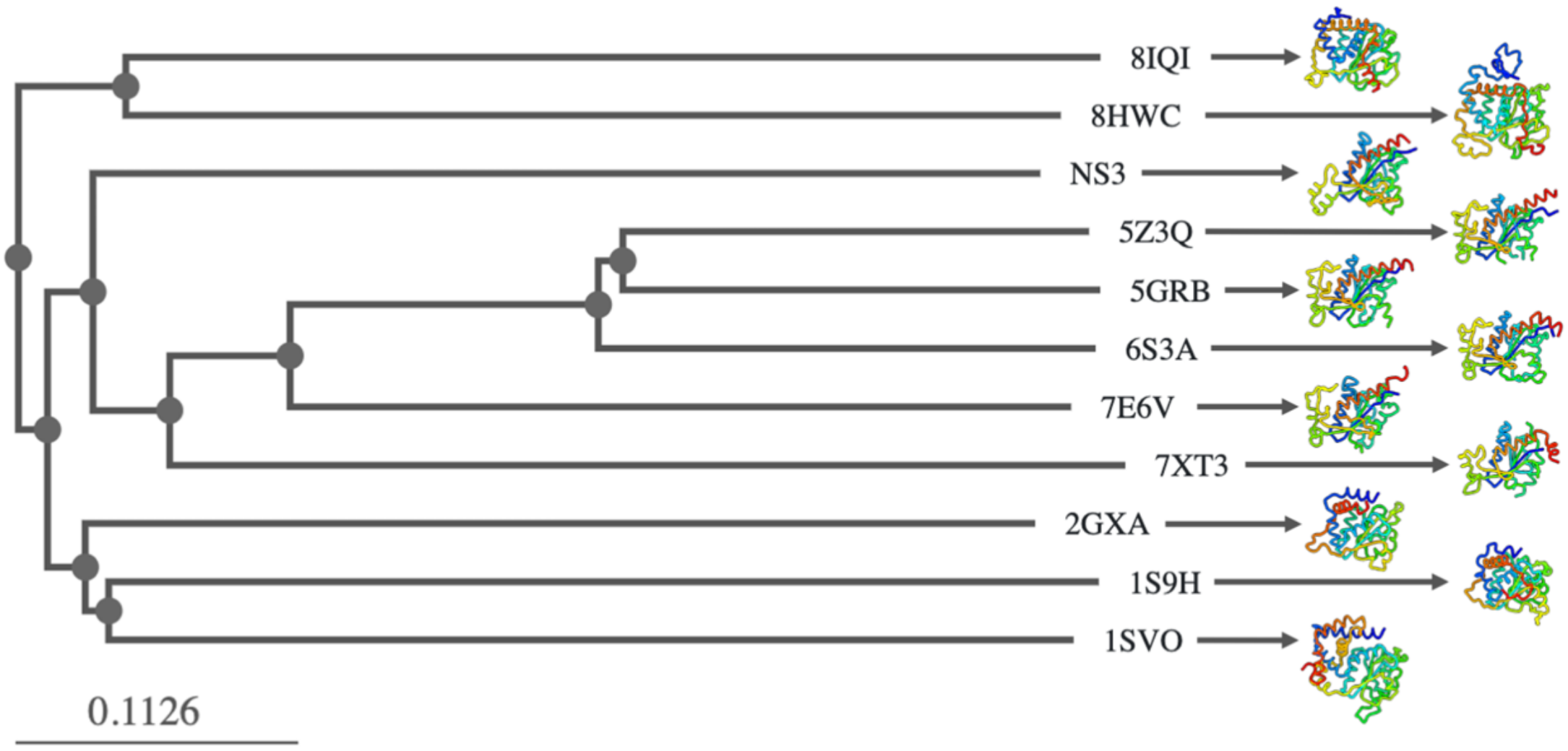

Supplementary Fig. 8: Structure-based phylogenetic classification of the NS3 AAA+ domain. Structure-based phylogenetic classification of experimentally determined AAA+ ATPase domains from DNA and RNA viruses calculated with FoldTree[8].

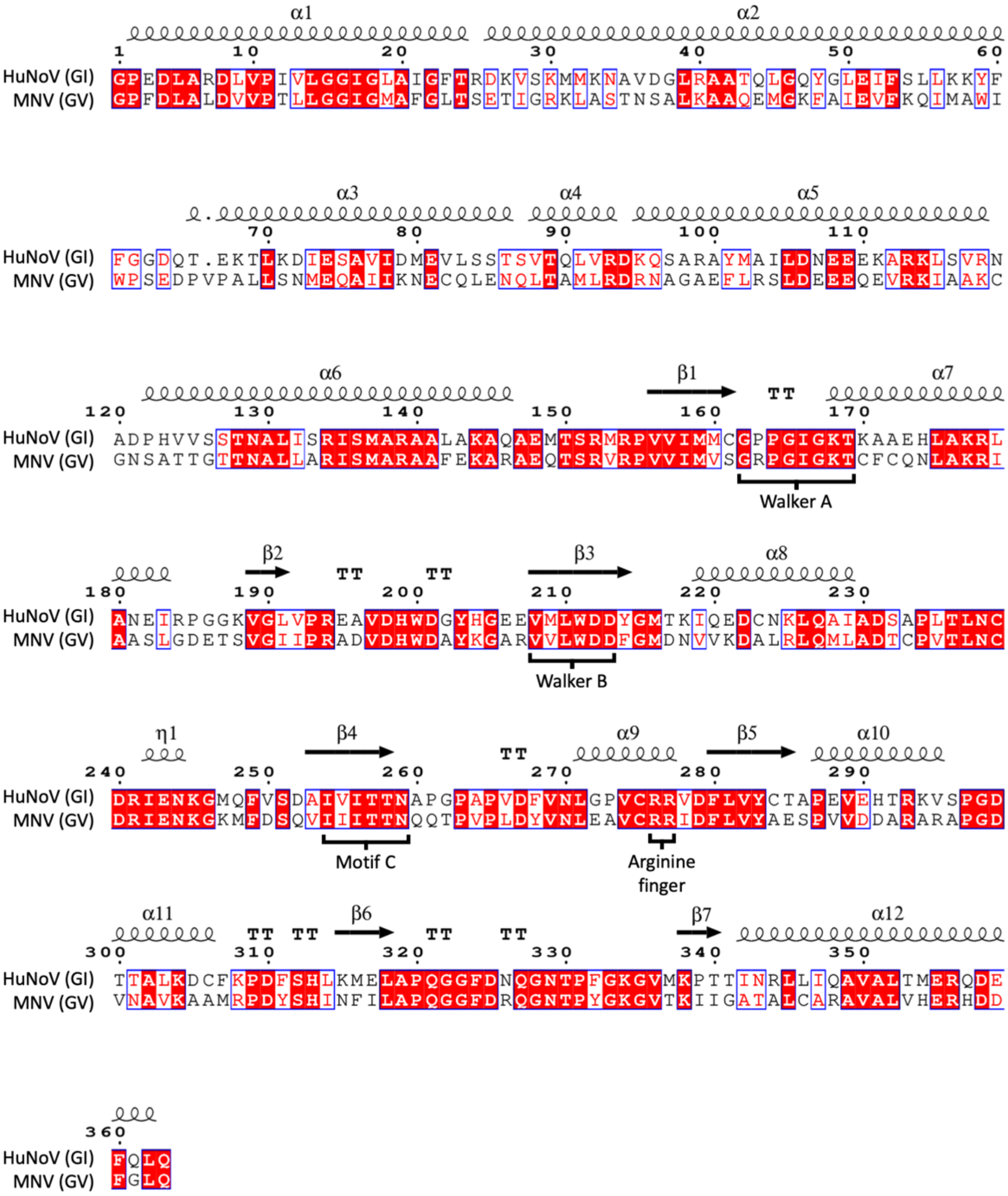

Supplementary Fig. 9: Sequence alignment of HuNoV and MNV NS3 sequences. Sequence alignment of HuNoV GI (NCBI Reference Sequence: NP_056820.1) and MNV GV (NCBI Reference Sequence: YP_724457.1) NS3 amino acid sequences. The sequence alignment was performed using Clustal Omega[9] and the image was generated by ESPript[10]. The secondary structure assignment, based on the HuNoV AlphaFold3 prediction, is shown. Signature SF3 helicase motifs are labeled.

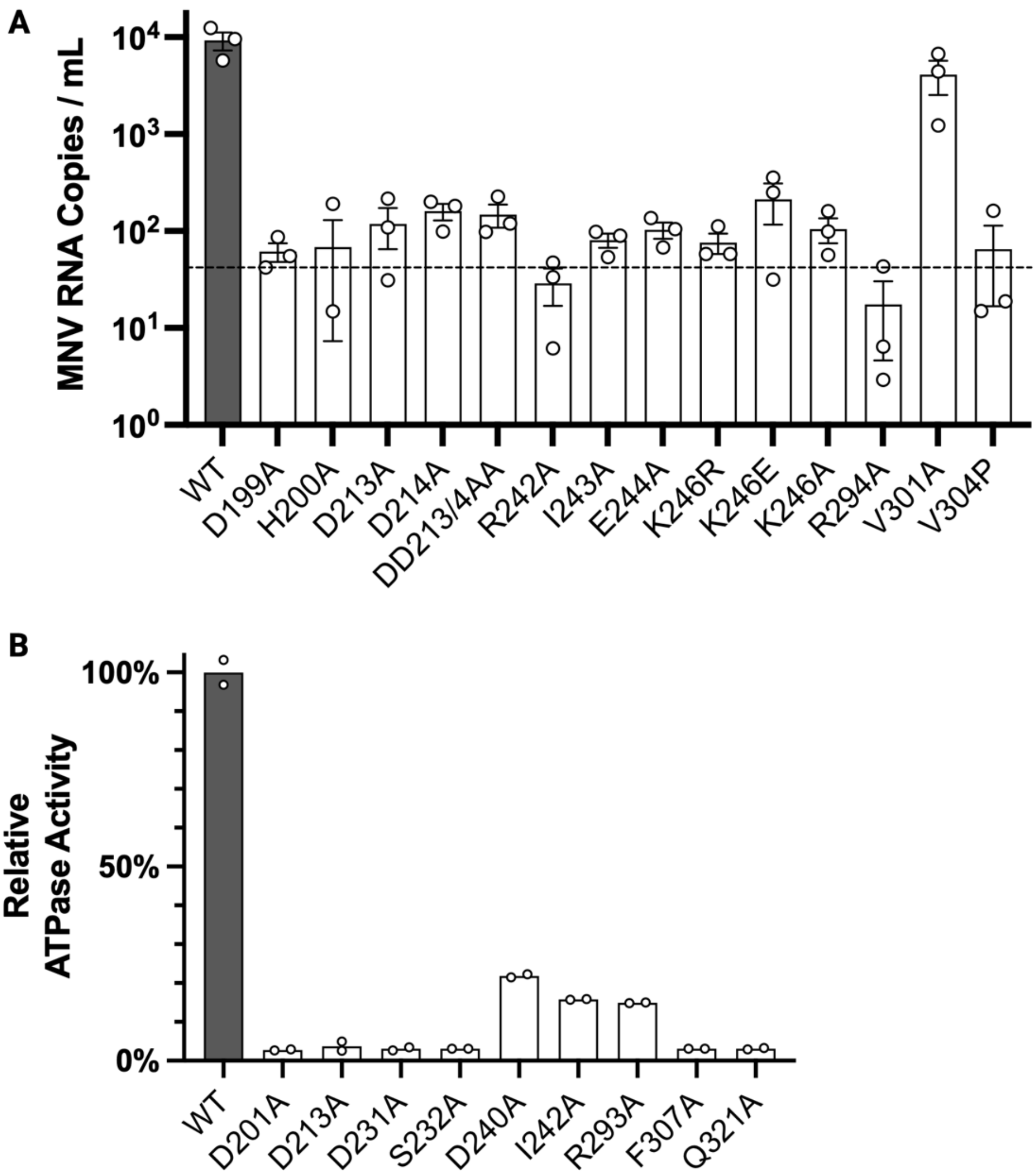

Supplementary Fig. 10. Mutational analysis of conserved NS3 structural elements in viral replication and ATPase activity. (A) Viral RNA accumulation following transfection of MNV genomes carrying the indicated NS3 mutations, measured by qRT-PCR and expressed as MNV RNA copies per mL. Bars show the mean ± SEM, with individual open circles representing independent biological replicates. The dashed line indicates the assay detection threshold. (B) ATPase activity of recombinant HuNoV hex-ΔN61-NS3 carrying the indicated mutations, normalized to wild-type activity. Representative data from one of two independent experiments, each performed in technical duplicate, are shown. Points show technical replicate measurements; bars show the mean. Source data are provided as a Source Data file.

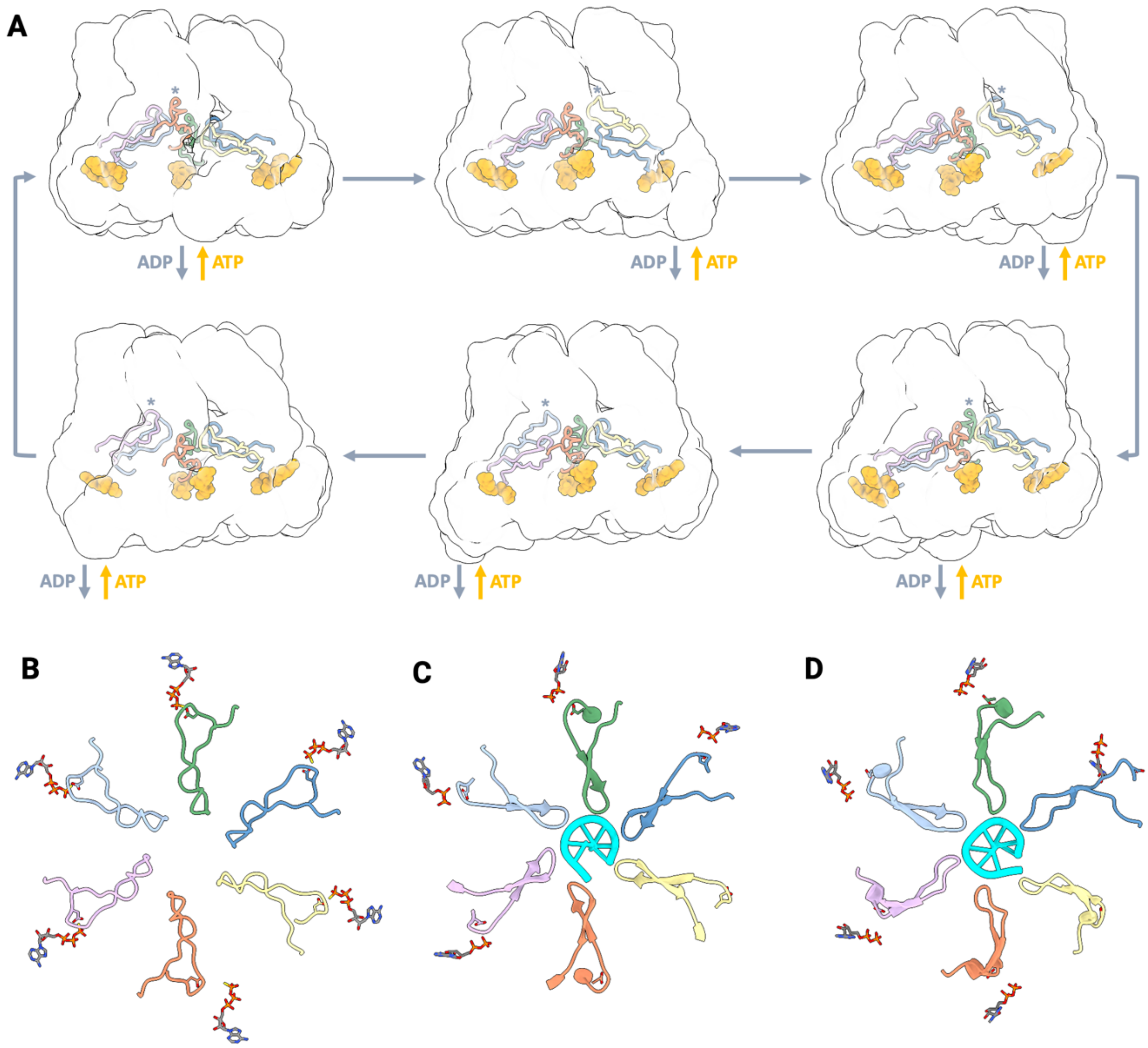

Supplementary Fig. 11: Proposed NS3 ATPase cycle and comparison of substrate-facing elements in SF3 ATPases. (A) The six images depict different steps in the ATP hydrolysis cycle of NS3. The ATP binding site at the seam of the complex, where ADP is released and a new ATP molecule binds, is shown empty. Binding of ATP to the nucleotide binding site of the subunit at position 6 (bottom of staircase) promotes its translocation to position 1 (top of the staircase). Consequently, each subunit moves to the next position in the staircase. For each step in the cycle, the uppermost pore loop 2 is indicated by an asterisk. (B) Comparison of substrate-facing elements in NS3 hexamer, (C) porcine circovirus 2 rep protein bound to single-stranded DNA (PDB ID: 7LAR), and (D) papillomavirus E1 hexameric helicase with ssDNA (PDB ID: 2GXA). In each case, an equivalently positioned aspartate residue lies between the substrate-facing element and the nucleotide-binding site.

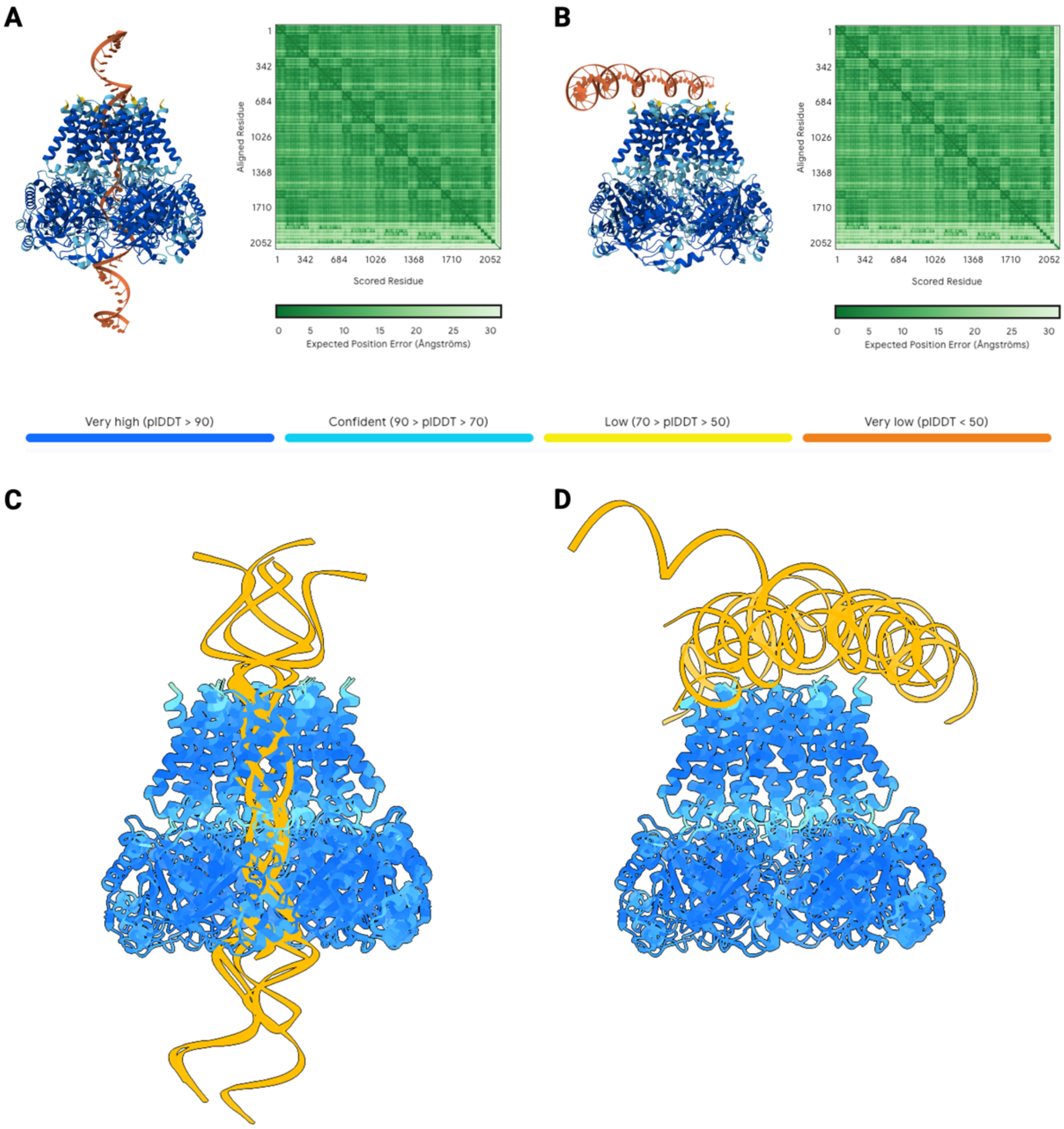

Supplementary Fig. 12. AlphaFold3 predictions of ΔN61-NS3 in complex with ssRNA or ssDNA. (A) AlphaFold3-predicted structure of the ΔN61-NS3 hexamer in the presence of ATP, Mg^{2+}, and a 50-nt poly(A) ssRNA. The protein backbone is shown in cartoon representation and colored by confidence score (predicted local distance difference test, pLDDT), with blue indicating high-confidence regions and orange indicating low-confidence regions. The predicted alignment error (PAE) plot is shown alongside the model to assess the reliability of structural domains and interdomain orientations. (B) As in (A), but predicted in the presence of a 50-nt poly(dA) ssDNA. (C) Superposition of the five ranked AlphaFold3 models from the ssRNA prediction shown in (A), highlighting consistent placement of ssRNA within the central pore. (D) Superposition of the five ranked AlphaFold3 models from the ssDNA prediction shown in (B), showing that ssDNA was not positioned within the central pore in any of the five ranked models.

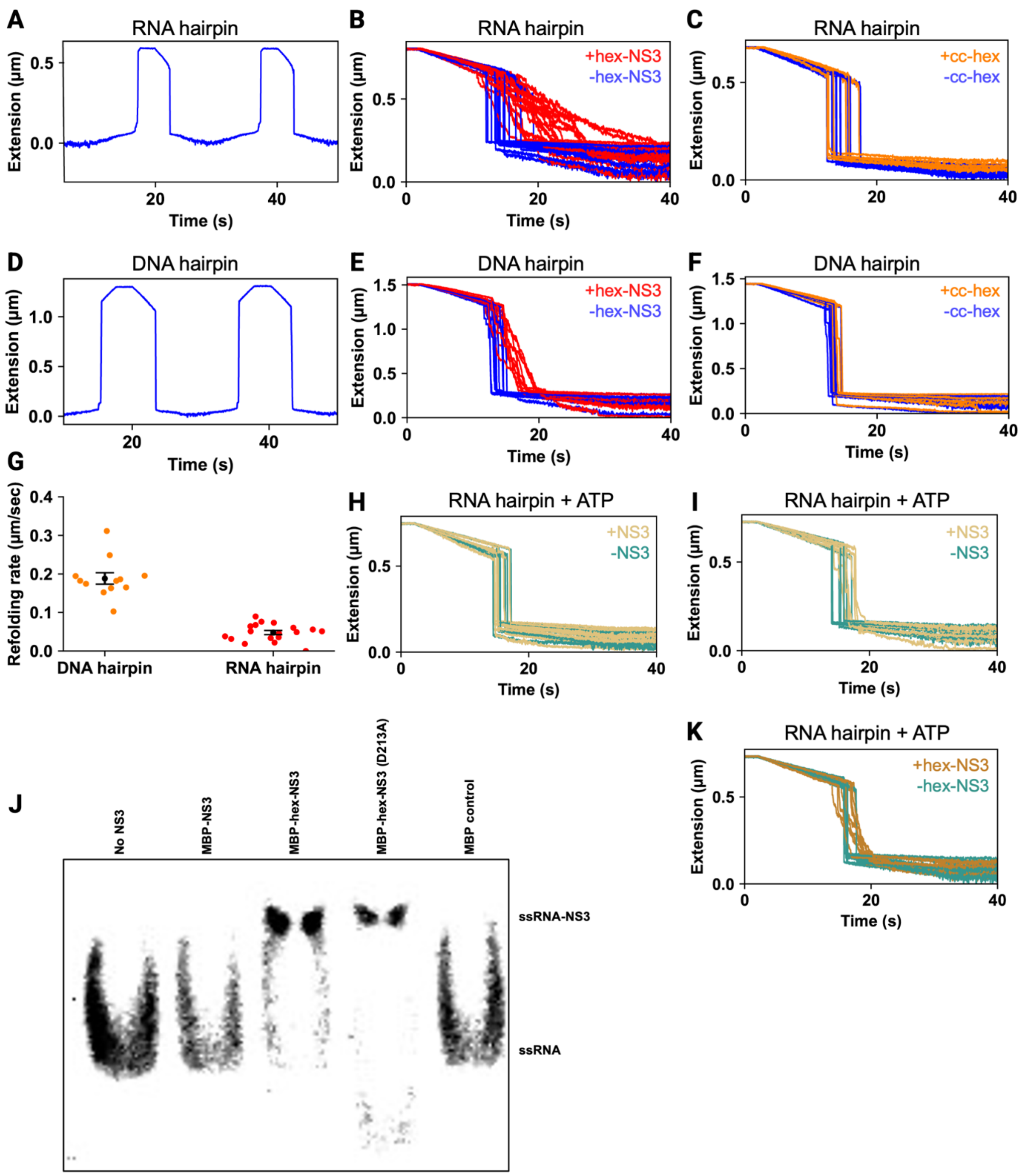

Supplementary Fig. 13. Oligomerization-dependent engagement of ssRNA by NS3 constructs. (A) Representative force-ramp trace from an RNA hairpin, showing hairpin unfolding at increasing force followed by refolding at decreasing force. (B) RNA hairpin refolding traces in the absence of ATP, measured without hex-ΔN61-NS3 (−hex-NS3, blue, n = 17) or with 1 μM hex-ΔN61-NS3 (+hex-NS3, red, n = 17). (C) RNA hairpin refolding traces in the absence of ATP, measured without synthetic cc-hex peptide (−cc-hex, blue, n = 9) or with 1 μM synthetic cc-hex peptide (+cc-hex,

orange, n = 9). (D) Representative force-ramp trace from a DNA hairpin. (E) DNA hairpin refolding traces in the absence of ATP, measured without hex-ΔN61-NS3 (−hex-NS3, blue, n = 12) or with 1 μM hex-ΔN61-NS3 (+hex-NS3, red, n = 12). (F) DNA hairpin refolding traces in the absence of ATP, measured without synthetic cc-hex peptide (−cc-hex, blue, n = 9) or with 1 μM synthetic cc-hex peptide (+cc-hex, orange, n = 9). (G) Refolding rates for DNA hairpins (orange, n = 12) and RNA hairpins (red, n = 18). The mean refolding rate is shown, error bars represent SEM, and individual points represent bead-averaged refolding rates. (H,I) Representative RNA hairpin refolding traces in the presence of 2 mM ATP, measured without ΔN61-NS3 (−NS3, teal, n = 10) or with 5 μM ΔN61-NS3 (+NS3, yellow, n = 10). The two examples illustrate the range of responses observed under the same conditions, from no detectable delay (H) to a slight refolding delay (I). (J) EMSA analysis of ssRNA binding by NS3 constructs using 20 μM protein, including ΔN61-NS3, hex-ΔN61-NS3, hex-ΔN61-NS3 Walker B mutant (D213A), and MBP control. Free ssRNA and shifted ssRNA-NS3 complexes are indicated. (K) RNA hairpin refolding traces in the presence of 2 mM ATP, measured without hex-ΔN61-NS3 (−hex-NS3, teal, n = 9) or with 1 μM hex-ΔN61-NS3 (+hex-NS3, orange, n = 9), showing reduced refolding delay compared with the no-ATP condition.

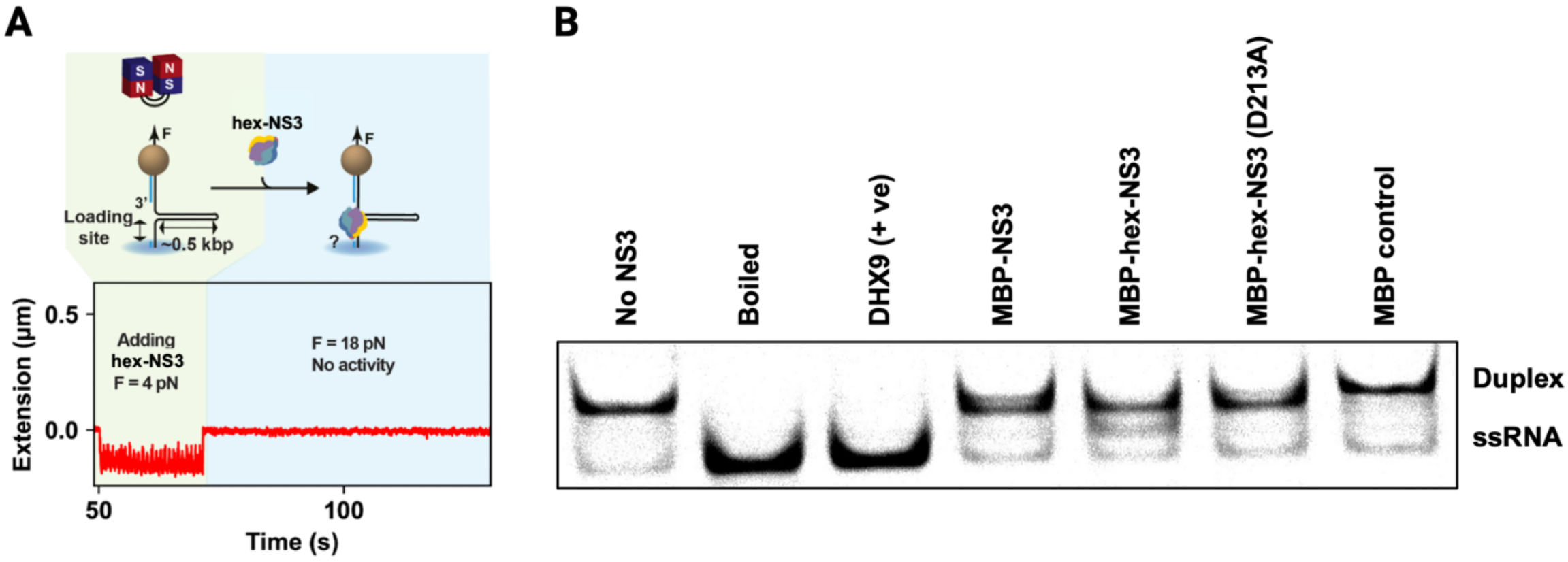

Supplementary Fig. 14. Hex-ΔN61-NS3 does not exhibit detectable RNA duplex unwinding activity under the conditions tested. (A) Schematic of the magnetic tweezers assay used to test RNA hairpin unwinding by hex-ΔN61-NS3. The RNA hairpin substrate contains an ssRNA loading site and is tethered between a magnetic bead and the flow-cell surface. Addition of 2.3 μM hex-ΔN61-NS3 and 2 mM ATP at low force did not result in detectable hairpin unwinding. (B) Gel-based helicase assay using a fluorescently labelled RNA duplex substrate containing a 3′ overhang. Reactions were performed with 20 μM protein and 2 mM ATP. Boiled substrate and DHX9 positive-control reactions indicate the positions of duplex and ssRNA species. No detectable RNA duplex unwinding was observed for ΔN61-NS3, hex-ΔN61-NS3, hex-ΔN61-NS3 Walker B mutant (D213A), or MBP control under these conditions.

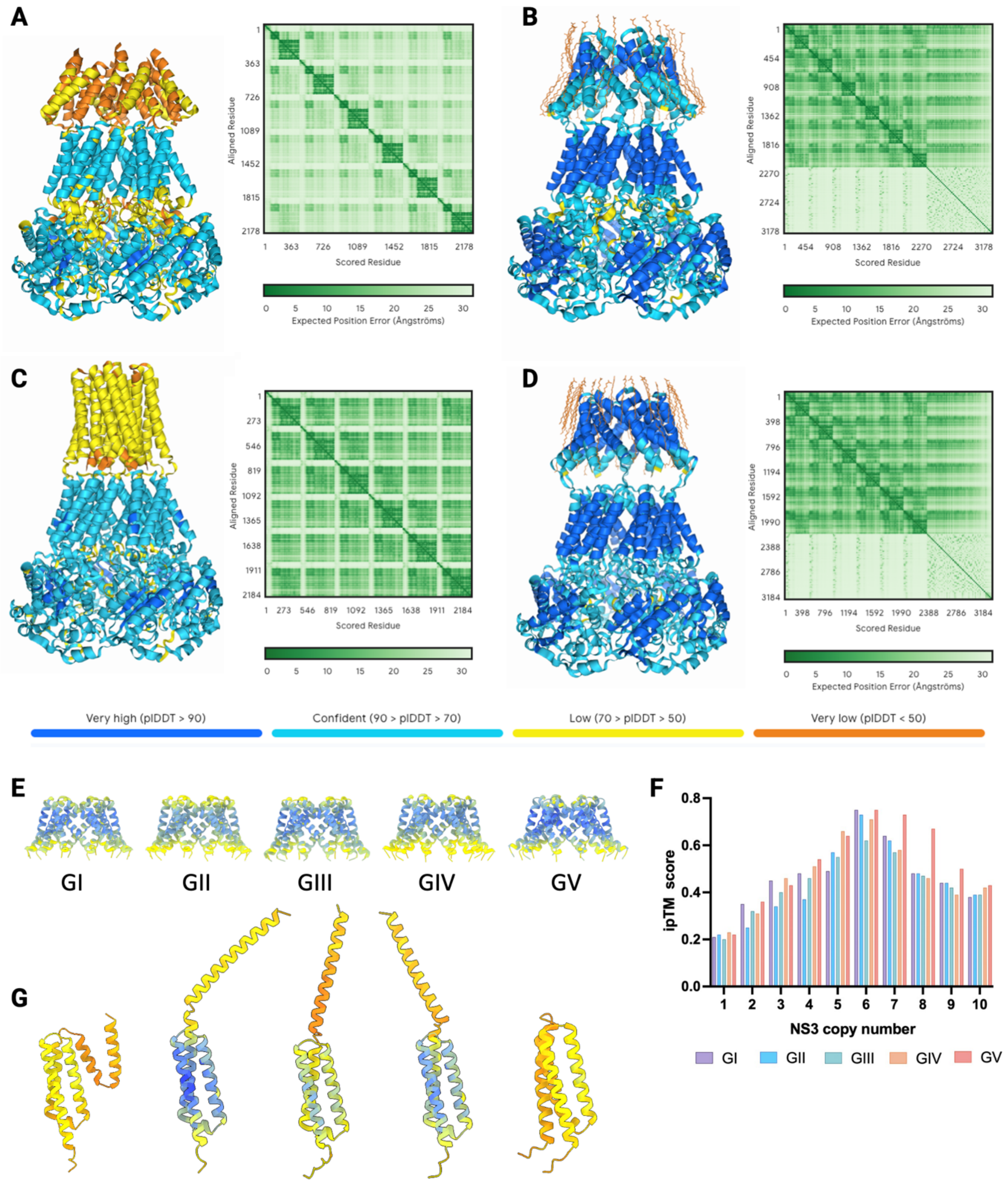

Supplementary Fig. 15. AlphaFold3 predictions support a conserved putative N-terminal transmembrane pore in full-length NS3. (A) AlphaFold3-predicted structure of the GI NS3 hexamer. The protein backbone is shown in cartoon representation and colored by confidence score (predicted local distance difference test, pLDDT), with blue indicating high-confidence regions and orange indicating low-confidence regions. The predicted alignment error (PAE) plot is shown alongside the

model to assess the reliability of structural domains and interdomain orientations. (B) As in (A), but with the GI NS3 hexamer predicted in the presence of 50 molecules of oleic acid to mimic a membrane-like environment. (C) Predicted structure of the GV NS3 hexamer, displayed as in (A). (D) As in (C), but with the GV NS3 hexamer predicted in the presence of 50 molecules of oleic acid. (E) AlphaFold3 predictions of full-length NS3 across norovirus genogroups I–V, performed with poly(A) RNA, ATP, Mg^{2+} and oleic acid molecules included as input components. For clarity, only the N-terminal domain is shown, highlighting the conserved putative transmembrane pore architecture. (F) Interface confidence (iPTM) scores for AlphaFold3 predictions of NS3 assemblies containing 1–10 subunits across norovirus genogroups I–V. Predictions were performed with poly(A) RNA, ATP, Mg^{2+} and oleic acid molecules included as input components. For all five genogroups, iPTM scores increased with copy number and peaked at the hexameric state, supporting a conserved preference for hexameric assembly. (G) AlphaFold3 predictions of monomeric NS3 truncation constructs using genogroup-specific domain boundaries corresponding to the previously proposed MLKL-like region yielded low-confidence and inconsistent models across norovirus genogroups I–V.

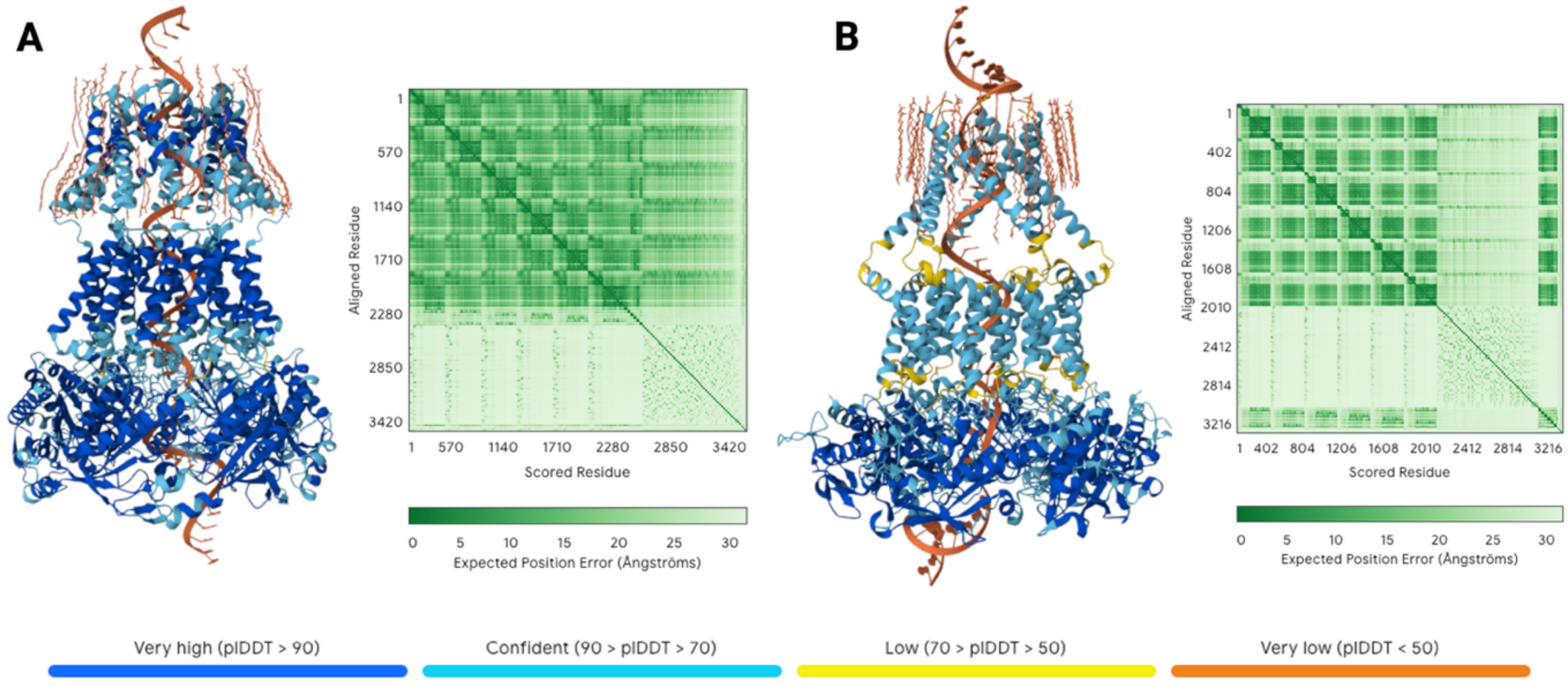

Supplementary Fig. 16. AlphaFold3 predictions of the full-length GI NS3 and Coxsackievirus B3 2C hexameric complexes in the presence of a membrane-mimicking environment, RNA, and co-factors. (A) AlphaFold3[11] predicted structure of the GI NS3 hexamer in complex with 50 molecules of oleic acid (to mimic a membrane environment), a 50-nt poly(A) RNA strand, 6 ATP molecules, and 6 magnesium ions. The protein backbone is shown in cartoon representation, colored by confidence score (predicted local distance difference test, pLDDT), with blue indicating high-confidence regions and red indicating low-confidence regions. The predicted alignment error (PAE) plot is included to evaluate the reliability of domain orientations and structural regions. (B) As in (A), but for the full-length Coxsackievirus B3 2C hexamer under identical conditions (50 oleic acid molecules, 50-nt poly(A) RNA, 6 ATP molecules, and 6 magnesium ions).

Supplementary Table 1: Data collection, image processing and refinement information

Cryo-EM data collection, refinement and validation statistics

	HuNoV NS3 (EMDB-53547) (PDB 9R34) (EMPIAR-12758)
**Data collection and processing**	
Magnification	130,000x
Voltage (kV)	200
Electron exposure (e–/Å^2)	50.5
Defocus range (μm)	0.75-1.5
Pixel size (Å)	0.9
Symmetry imposed	C1
Initial particle images (no.)	1,229,229
Final particle images (no.)	123,643
Map resolution (Å)	2.9
FSC threshold	0.143
Map resolution range (Å)	2-34
**Refinement**	
Initial model used (PDB code)	AlphaFold2
Model resolution (Å)	2.8
FSC threshold	0.5
Model resolution range (Å)	2.6-2.8
Map sharpening *B* factor (Å^2)	64
Model composition	
Non-hydrogen atoms	14225
Protein residues	1800
Ligands	12
B factors (Å^2)	
Protein	41.8
Ligand	23.4
R.m.s. deviations	
Bond lengths (Å)	0.004
Bond angles (°)	0.584
Validation	
MolProbity score	1.59
Clashscore	4.9
Poor rotamers (%)	1.84
Ramachandran plot	
Favored (%)	97.32
Allowed (%)	2.68
Disallowed (%)	0

References

1. Jumper, J. *et al.* Highly accurate protein structure prediction with AlphaFold. *Nature* **596**, (2021).
2. Zaccai, N. R. *et al.* A de novo peptide hexamer with a mutable channel. *Nat Chem Biol* **7**, (2011).
3. Hurdiss, D. L. *et al.* Fluoxetine targets an allosteric site in the enterovirus 2C AAA+ ATPase and stabilizes a ring-shaped hexameric complex. *Sci Adv* **8**, (2022).
4. Shi, H., Rampello, A. J. & Glynn, S. E. Engineered AAA+ proteases reveal principles of proteolysis at the mitochondrial inner membrane. *Nat Commun* **7**, (2016).
5. Tan, Y. Z. *et al.* Addressing preferred specimen orientation in single-particle cryoEM through tilting. *Nat Methods* **14**, 793–796 (2017).
6. Punjani, A., Rubinstein, J. L., Fleet, D. J. & Brubaker, M. A. CryoSPARC: Algorithms for rapid unsupervised cryo-EM structure determination. *Nat Methods* **14**, (2017).
7. Afonine, P. V. *et al.* Real-space refinement in PHENIX for cryo-EM and crystallography. *Acta Crystallogr D Struct Biol* **74**, (2018).
8. Moi, D. *et al.* Structural phylogenetics unravels the evolutionary diversification of communication systems in gram-positive bacteria and their viruses. *bioRxiv* (2023).
9. Sievers, F. *et al.* Fast, scalable generation of high-quality protein multiple sequence alignments using Clustal Omega. *Mol Syst Biol* **7**, (2011).
10. Robert, X. & Gouet, P. Deciphering key features in protein structures with the new ENDscript server. *Nucleic Acids Res* **42**, (2014).
11. Abramson, J. *et al.* Accurate structure prediction of biomolecular interactions with AlphaFold 3. *Nature* **630**, 493–500 (2024).
