# Supplementary material for: Integrative structure of norovirus NS3 suggests a role in RNA transport
Source: Nat Commun. 2026 Sep 21;17:10991. doi: 10.1038/s41467-026-77946-7 (PMC13683342; doi:10.1038/s41467-026-77946-7)
Supplement: Supplementary file 2 [file 41467_2026_77946_MOESM2_ESM.pdf]

Description of Additional Supplementary Files

File name: Supplementary Movie 1
Description: Surface representation of the integrative model of the full-length HuNoV NS3 complex embedded in a membrane bilayer (left), and a central cross-section (right) highlighting a putative RNA translocation pathway. This animation illustrates the sequential ATP-driven conformational changes of the NS3 hexamer, consistent with a hand-over-hand translocation mechanism. Created in BioRender. Hurdiss, D. (2026) https://BioRender.com/6a0zadu.
