# Supplementary material for: Integrative structure of norovirus NS3 suggests a role in RNA transport
Source: Nat Commun. 2026 Sep 21;17:10991. doi: 10.1038/s41467-026-77946-7 (PMC13683342; doi:10.1038/s41467-026-77946-7)
Supplement: Supplementary file 3 [file 41467_2026_77946_MOESM4_ESM.pdf]

Corresponding author(s): Daniel L. Hurdiss

Last updated by author(s): Aug 13, 2026

Reporting Summary

Nature Portfolio wishes to improve the reproducibility of the work that we publish. This form provides structure for consistency and transparency in reporting. For further information on Nature Portfolio policies, see our Editorial Policies and the Editorial Policy Checklist.

Statistics

For all statistical analyses, confirm that the following items are present in the figure legend, table legend, main text, or Methods section.

n/a	Confirmed	
☐	☒	The exact sample size (*n*) for each experimental group/condition, given as a discrete number and unit of measurement
☐	☒	A statement on whether measurements were taken from distinct samples or whether the same sample was measured repeatedly
☒	☐	The statistical test(s) used AND whether they are one- or two-sided<br>*Only common tests should be described solely by name; describe more complex techniques in the Methods section.*
☒	☐	A description of all covariates tested
☒	☐	A description of any assumptions or corrections, such as tests of normality and adjustment for multiple comparisons
☐	☒	A full description of the statistical parameters including central tendency (e.g. means) or other basic estimates (e.g. regression coefficient) AND variation (e.g. standard deviation) or associated estimates of uncertainty (e.g. confidence intervals)
☒	☐	For null hypothesis testing, the test statistic (e.g. *F*, *t*, *r*) with confidence intervals, effect sizes, degrees of freedom and *P* value noted<br>*Give P values as exact values whenever suitable.*
☒	☐	For Bayesian analysis, information on the choice of priors and Markov chain Monte Carlo settings
☒	☐	For hierarchical and complex designs, identification of the appropriate level for tests and full reporting of outcomes
☒	☐	Estimates of effect sizes (e.g. Cohen's *d*, Pearson's *r*), indicating how they were calculated

Our web collection on statistics for biologists contains articles on many of the points above.

Software and code

Policy information about availability of computer code

Data collection	Cryo-EM data were collected using EPU acquisition software version 2.8.1 on a Thermo Scientific Glacios Cryo-TEM equipped with a Falcon 4i direct electron detector and Selectris energy filter. Mass photometry data were collected using AcquireMP v2023 R1.1 (Refeyn Ltd). Magnetic tweezers data were acquired using established custom instrument-control software. No novel custom code or unpublished software central to the conclusions of the study was used for data collection.
Data analysis	Cryo-EM image processing was performed in cryoSPARC version 4.1.0. Model fitting, refinement, validation and visualization were performed using UCSF Chimera version 1.16, Namdinator, Coot version 0.9.8.1, Phenix version 1.19.2-4158, MolProbity version 4.02-528, UCSF ChimeraX version 1.5, and ISOLDE version 1.4. Structural analyses used PDBePISA version 1.52, LigPlot+ version 2.2.8, CASTpFold, Foldseek, DALI and FoldTree. Sequence alignment and visualization used Clustal Omega version 1.2.4 and ESPript version 3.0. AlphaFold2 predictions were performed using the COSMIC² server, and AlphaFold3 predictions were performed using the AlphaFold Server. Mass photometry data were analysed using DiscoverMP v2023. Native mass spectrometry spectra were inspected and exported using Thermo FreeStyle (version not recorded) and analysed using UniDec version 6.0.4. Magnetic tweezers data were analysed using previously established MATLAB and Python analysis scripts. Statistical analysis and data visualization were performed using GraphPad Prism version 10. No new software central to the conclusions of this study was developed. Software versions are reported where recorded and applicable.

For manuscripts utilizing custom algorithms or software that are central to the research but not yet described in published literature, software must be made available to editors and reviewers. We strongly encourage code deposition in a community repository (e.g. GitHub). See the Nature Portfolio guidelines for submitting code & software for further information.

Data

Policy information about availability of data

All manuscripts must include a data availability statement. This statement should provide the following information, where applicable:

- Accession codes, unique identifiers, or web links for publicly available datasets
- A description of any restrictions on data availability
- For clinical datasets or third party data, please ensure that the statement adheres to our policy

Atomic coordinates have been deposited in the Protein Data Bank under accession code 9R34. The corresponding EM density maps, including the final unsharpened, sharpened, local resolution-filtered maps, half maps, and mask, have been deposited in the Electron Microscopy Data Bank under accession code EMD-53547. Unaligned gain-normalized movies, motion-corrected micrographs, and the final refined particle stacks have been deposited in the Electron Microscopy Public Image Archive under accession code EMPIAR-12758. The integrative model of the full-length NS3 hexamer, AlphaFold prediction outputs, and raw and processed native mass spectrometry and mass photometry data used in this study are available through Zenodo entry 15556769 [https://doi.org/10.5281/zenodo.15556769]. The magnetic tweezers data generated in this study are available through the Yoda data repository [https://doi.org/10.48338/VU01-U0QASY]. Source data are provided with this paper. Requests for reagents should be directed to the corresponding author, D.L.H.

Research involving human participants, their data, or biological material

Policy information about studies with human participants or human data. See also policy information about sex, gender (identity/presentation), and sexual orientation and race, ethnicity and racism.

Reporting on sex and gender	N/A
Reporting on race, ethnicity, or other socially relevant groupings	N/A
Population characteristics	N/A
Recruitment	N/A
Ethics oversight	N/A

Note that full information on the approval of the study protocol must also be provided in the manuscript.

Field-specific reporting

Please select the one below that is the best fit for your research. If you are not sure, read the appropriate sections before making your selection.

☒ Life sciences ☐ Behavioural & social sciences ☐ Ecological, evolutionary & environmental sciences

For a reference copy of the document with all sections, see nature.com/documents/nr-reporting-summary-flat.pdf

Life sciences study design

All studies must disclose on these points even when the disclosure is negative.

Sample size	No statistical method was used to predetermine sample size. For cryo-EM, sample size was determined by available microscope time and by the number of particles required to obtain a reconstruction at the reported resolution, as assessed by Fourier shell correlation. One cryo-EM dataset was collected, comprising 1,010 movies and 1,229,229 initially picked particles. After 2D classification, heterogeneous refinement and non-uniform refinement, 123,643 particles contributed to the final 2.9 Å reconstruction.<br><br>Biochemical experiments were independently repeated on at least two separate days, with each experiment performed in technical duplicate or greater. Virus infection experiments were performed in at least three independent biological replicates, each including technical duplicates.
Data exclusions	For cryo-EM image processing, particles assigned to poorly defined or junk classes during 2D classification, ab initio reconstruction, and heterogeneous refinement were excluded according to standard single-particle cryo-EM processing workflows. No data were excluded from biochemical or virus infection analyses.
Replication	The cryo-EM dataset was collected once and processed according to standard single-particle cryo-EM workflows. Two independent half-maps were refined to estimate map resolution using the gold-standard Fourier shell correlation criterion. Biochemical experiments were independently repeated on at least two separate days, with at least technical duplicates. Virus infection experiments were performed in at least three independent biological replicates, each including technical duplicates.
Randomization	No randomization was used. For cryo-EM processing, particles were split into two independent half sets during refinement according to standard single-particle cryo-EM procedures.

Blinding	Blinding was not used. The study did not involve treatment allocation or subjective outcome assessment where blinding would be applicable.

Reporting for specific materials, systems and methods

We require information from authors about some types of materials, experimental systems and methods used in many studies. Here, indicate whether each material, system or method listed is relevant to your study. If you are not sure if a list item applies to your research, read the appropriate section before selecting a response.

Materials & experimental systems

n/a	Involved in the study
☐	☒ Antibodies
☐	☒ Eukaryotic cell lines
☒	☐ Palaeontology and archaeology
☒	☐ Animals and other organisms
☒	☐ Clinical data
☒	☐ Dual use research of concern
☒	☐ Plants

Methods

n/a	Involved in the study
☒	☐ ChIP-seq
☒	☐ Flow cytometry
☒	☐ MRI-based neuroimaging

Antibodies

Antibodies used	anti-digoxigenin (Roche - Cat. No. 11333089001).
Validation	https://www.sigmaaldrich.com/NL/en/product/roche/11333089001#product-documentation

Eukaryotic cell lines

Policy information about cell lines and Sex and Gender in Research

Cell line source(s)	BV-2 (murine microglial cell line) – gifted by Ian Goodfellow, University of Cambridge<br><br>BHK-21 (baby hamster kidney cells) – obtained from ATCC<br><br>HEK293T (human embryonic kidney cells) – obtained from ATCC
Authentication	No specific authentication of cell lines was performed for this study. All lines were obtained from reputable sources or previously published work and were used within low passage numbers.
Mycoplasma contamination	All cell lines were routinely tested and confirmed negative for mycoplasma contamination.
Commonly misidentified lines<br>(See ICLAC register)	None of the cell lines used are listed on the ICLAC register of commonly misidentified cell lines.

Plants

Seed stocks	N/A
Novel plant genotypes	N/A
Authentication	N/A
