# Supplementary material for: Integrative structure of norovirus NS3 suggests a role in RNA transport
Source: Nat Commun. 2026 Sep 21;17:10991. doi: 10.1038/s41467-026-77946-7 (PMC13683342; doi:10.1038/s41467-026-77946-7)
Supplement: Supplementary file 4 [file 41467_2026_77946_MOESM5_ESM.pdf]

Peer Review File

# Integrative structure of norovirus NS3 suggests a role in RNA transport

Corresponding Author: Dr Daniel Hurdiss

**This file contains all reviewer reports in order by version, followed by all author rebuttals in order by version.**

Version 0:

Reviewer comments:

Reviewer #1

(Remarks to the Author)
In this manuscript, Haas and colleagues present a structure based on cryo-EM SPA of the Human Norovirus NS3 protein, a RNA helicase belonging to the helicase superfamily (SF) 3 according to sequence alignments. The structure was determined for a truncated version lacking the N-terminal transmembrane domain.

In brief, the authors generated a DeltaN61-version of the protein carrying an additional hexamerization motif at the N-terminus. In absence of this motif, the authors did not observe hexers using mass photometry. The authors showed hydrolysis activity of nucleotides, preferably ATP and GTP, and inhibited nucleotide activity in presence of ATPgS. Subsequently the authors prepared cryo-EM grids followed by imaging and particle averaging and electron density fitting with an alpha fold 2 guess of the NS3 hexamer structure. The authors revealed potential RNA interacting residues and resolved a lock-washer overall architecture of the helicase domain. The authors present also single-molecule data to show ssRNA and dsRNA binding. With an alpha fold 3 model the authors perform integrative prediction of how NS3 could be arranged on a lipid membrane and pump ssRNA across the membrane for particle packing.

The structure is well described and convincing as well as in line with earlier structures of SF3 helicases also SF3 DNA helicases. The manuscript is well written, though I have a few questions that should be addressed prior to publication:

- The authors report at the beginning of their study that the hexamerization motif cc-hex was required to see hexamerization of their truncated protein using mass photometry. Yet, this is a very artificial motif and already the individual monomers of the AAA+ enzyme should show a propensity for hexamerization. I was wondering why the authors preformed hexamerisation experiments in absence of ssRNA? For helicases, nucleic acid substrates often act as templating structures.

- The authors use in the beginning of their studies Alphafold 2, at the end Alphafold 3. What would happen if for the initial prediction alpha fold 3 would be used?

- I am also puzzled about the hydrolysis activity tests. It seems these assays were performed in absence of ssRNA, suggesting that the reported activity results from futile triphosphate hydrolysis. Did the authors test ssRNA stimulated nucleotide hydrolysis?

- Along the same line the dose dependent reduced ATPase activity by addition of ATPgS suggests a significantly higher affinity to ATP, yet this might be different if ssRNA is present. If the hydrolysis is as tightly regulated one subunit at a time, a single ATPgS bound might already be efficient to inhibit the hydrolysis of subsequent subunits (see e.g. Schlierf ... Ha eLife 2019). This should be tested and can also verify the tightly-regulated ATP hydrolysis model suggested by the authors. (Side note: ATPgS affinity could be also determined with a displacement assay based on mant-ADP).

- The authors probe the unwinding activity using a sophisticated magnetic tweezers assay. Unfortunately, the authors do not observe unwinding, though we remain unclear if the purified enzyme is at all unwinding competent. Does the hexer assemble on the short ssRNA, does it require a partial duplex, does it show translocation activity (could be probed using correlative imaging and force spectroscopy (e.g. C-Trap as developed in one of the co-authors institute or in the hairpin assay with mechanical hairpin opening and closing as reported in earlier studies by Croquette or Bensimon). Therefore I suggest simple unwinding assays based on forked, marked(fluorescent or 32P-) RNA substrates followed by quenching and

gel electrophoresis if any information on unwinding should be reported herewith. The authors further conclude from their single molecule experiments ssRNA binding, which again would be more convincing to test using EMSA.

- An additional question arises, from Figure S11, where the authors state that their single-molecule assays were performed in absence of ATP. How can this then lead to translocation/unwinding?

- Similar, with the authors second set of single-molecule experiments they find indications for dsRNA binding, something which could and should be verified using EMSA. (the supplementary figure 11 was hard to read as the arrangement of panels was in a zig-zag fashion). Is there any biochemical reason for dsRNA binding? Is the binding of a hexamer or is it non-specific charge mediated backbone binding of a monomer? (Could be tested with their non-hex variant?) - though: It also seems that the dsRNA single-molecule data show a distinct long living collapsed state shortly below 0.5 µm. What is this state? How well defined is it? Can one correlate it with a sequence element or an unligated ssRNA exposure due to the sample preparation which is present at a distinct position in the RNA sequence? Even though the data quality is high, it reports no insights, thus at this stage the single-molecule data presented in the main text or supplementary information do not add to the manuscript and findings of the structure in my view, they rather confuse the reader.

Reviewer #2

(Remarks to the Author)
This manuscript reports a near-atomic resolution cryo-EM structure of the oligomeric norovirus NS3 protein in engineered, soluble form and bound to ATPγS, and integrates these data with AlphaFold3 predictions to propose a full-length, membrane-embedded model. The work addresses an enzyme of clear biomedical relevance and situates its activity within the context of viral replication organelles, offering a timely contribution to our understanding of norovirus replication.

Strengths
• The cryo-EM structure is of high quality and establishes the oligomeric state of NS3 at near-atomic resolution.
• The model confirms the canonical AAA+ helicase fold and lock-washer architecture consistent with translocation.
• The integration of cryo-EM data with AlphaFold3 modeling offers a coherent framework for considering how NS3 engages RNA in the context of membrane association.
• The study provides a structural basis for interpreting prior functional reports and contributes a valuable reference for the SF3 helicase family.
• The accompanying mutational analysis supports the structural findings and provides important functional context, strengthening the link between the proposed architecture and NS3 activity.

Limitations, concerns, and suggestions
The experimental cryo-EM structure is confirmatory rather than fundamentally novel: it reproduces the fold and oligomeric state predicted by AlphaFold and already known from related SF3 helicases (e.g., SV40 LTag, papillomavirus E1). The lock-washer arrangement is also expected, as it has been observed in other SF3 helicases solved in the presence of nucleic acids. A notable difference is the helical bundle domain (HBD), which in NS3 appears asymmetric, unlike the planar N-tiers of other SF3 helicases that act as oligomerization domains. In this case, the authors plausibly propose that oligomer stabilization is instead mediated by the N-domain (absent in the cryo-EM reconstruction).

The mechanistic claims (hand-over-hand RNA translocation, dual RNA–protein translocation involving VPg) are inferred rather than demonstrated, as no RNA substrate is present in the structure. These should be presented as hypotheses consistent with the architecture rather than as findings. It would also be helpful to clarify the single-molecule data on RNA binding and provide direct biochemical evidence for RNA engagement (e.g., EMSA). Since AlphaFold predictions are presented with RNA, the authors could comment on predicted interfaces and whether these resemble interactions seen in other SF3 helicases.

One potentially interesting feature in the structure is the apparent direct interaction of the pore loops with nucleotide cofactors, which has not been reported for E1, SV40, or CMG. The authors should discuss how this might influence their proposed translocation mechanism. Is the two-loop architecture analogous to that described for CMG?

Finally, the membrane-binding fold is not experimentally resolved here, and the proposed model diverges from earlier predictions (e.g., the MLKL-like fold reported in Nature 2023). The authors should discuss these differences and provide support for their model.

Experiments that could strengthen the manuscript
1. Since the central mechanistic claims depend on RNA engagement, an RNA-bound cryo-EM reconstruction (even at modest resolution) would strongly substantiate the proposed translocation model and clarify the role of pore loops.

2. The central channel is reported as negatively charged, which would disfavor stable nucleic acid binding. The suggestion of transient interactions is compelling but could be tested explicitly, for example by RNAse protection assays.

Minor comments
1. The reported map resolution may not support unambiguous water placement throughout. Please avoid strong claims of water-mediated interactions unless the density is clear; otherwise, mark such waters as putative.

2. Lines 556–557: Please specify the EM grid material (gold or copper) and the mesh size.
3. Lines 570–572: Please indicate the particle diameter used for template picking.
4. Line 602: Correct “secondary restraints” to “secondary structure restraints.”

Reviewer #3

(Remarks to the Author)
I co-reviewed this manuscript with one of the reviewers who provided the listed reports. This is part of the Nature Communications initiative to facilitate training in peer review and to provide appropriate recognition for Early Career Researchers who co-review manuscripts.

Reviewer #4

(Remarks to the Author)
This manuscript aims to provide a semi-predictive model of the norovirus NTPase/Helicase protein NS3, and to propose a molecular model whereby the function of the protein is to export a ssRNA viral RNA molecule across a cellular membrane via pore. The NS3 protein is known to be multi-functional and the authors here provide the first structural data for how NS3 may form multimers to assemble a functional complex. The cryo-EM is combined with predictive AF3 modelling that overall is convincing in revealing a hexamer of the proteins that assembles on a cellular membrane. Supporting data is provided in defining key residues that support the individual subunit interactions and within associated lipid and nucleotide molecules. Overall and very informative study with some aspects to be addressed to completely support the conclusions made

Major issues to be addressed:
- One of the key aspects of this project is whether the hexameric form of NS3 is the authentic form observed in infected cells. Of course, this is challenging to determine but is critical to the overall conclusions described here.
- The authors should attempt to purify full length NS3 from transfected cells, after mild detergent and affinity purification followed by SEC and native PAGE gel. Alternative would be to use chemical cross-linking in cells and isolate NS3
- So just to confirm, the monomeric or dimeric MBP-NS3 did not have ATPase activity? i.e. was the oligomeric hexameric form strictly required for this activity? Did the other oligomeric forms ie. trimer, tetramer, pentamer etc also have ATPase activity, and to what degree? Does incubation of the MBP-NS3, rather than hex-NS3, with ATP S stabilize any additional oligomeric structures?
- In Fig 3, what was the rationale for choosing the selected mutants? I understand that these were to disrupt cation-interaction and helix-turn-helix motif but the selected amino acids don’t completely correspond with those identified in the structure. So, the rationale seems disconnected from the structure. I would suggest to complement the study by introducing the corresponding amino acids from GI e.g. D292 with E and K/R to confirm that the charge is required.
- Additionally, the impact observed on MNV replication should be extended beyond just TCID50. The authors should also measure genome amplification via qRT-PCR and protein production via western blot to determine if these mutations are affecting early events (as predicted) rather than just virus release.
- How do the mutations introduced into MNV in Fig 3I (and Fig 4) affect the overall predicted structure of the NS3 hexamer? Do all of the mutations in MNV also affect the ATPase activity? This needs to be confirmed to relate structure and function
- In Fig 5B and C do the DNA helicases have an NTD that is also membrane associated? Essentially asking if the lack of interactions between the NS3 HBDs in this model are due to the absence of the membrane?
- For the molecular tweezers experiments was the monomeric/dimeric forms of NS3 also tested? It would be interesting to consider the compartmentalisation of function with the helicase activity more “internal” to the viral replication complex and potentially coordinated via an interaction with Pol or ProPol on the dsRNA template

Version 1:

Reviewer comments:

Reviewer #1

(Remarks to the Author)
In this revised manuscript, Haas and colleagues present a structure of the human norovirus NS3 protein, a SF3 RNA helicase. I appreciate very much how the authors have considered the comments of all reviewers and have further improved the manuscript. With the revision, the authors have added a lot of new biochemical and biophysical data to strengthen their findings. These additional data carefully characterise activity and function. I also appreciate that the authors have clarified some of the confusion arising from some original single-molecule assays and removed confusing and hard to interpret data, but also derived new assays and added those data. Sadly, helicase activity is still not directly or biochemically visible, though I agree with the authors suspicion that a full length protein embedded in a lipid bilayer might then reveal this activity. Having said that I wish the authors all the best for their next adventure on full length protein structure and function studies.

All my comments have been sufficiently addressed.

Reviewer #2

(Remarks to the Author)

The authors have addressed all of the points raised in my initial review, and the revised manuscript is substantially strengthened by the addition of new computational, biochemical, and virological data.

Reviewer #3

(Remarks to the Author)
I co-reviewed this manuscript with one of the reviewers who provided the listed reports. This is part of the Nature Communications initiative to facilitate training in peer review and to provide appropriate recognition for Early Career Researchers who co-review manuscripts.

Reviewer #4

(Remarks to the Author)
I thank the authors for making a concerted effort to address the comments identified from the original submission. Their additional data greatly aids with the conclusions and is of a very high quality. The issue of the hexameric form of NS3 within infected cells still remains unknown, but is technically difficult and challenging at this current time. I have no additional comments with this revised submission.

**Open Access** This Peer Review File is licensed under a Creative Commons Attribution 4.0 International License, which permits use, sharing, adaptation, distribution and reproduction in any medium or format, as long as you give appropriate credit to the original author(s) and the source, provide a link to the Creative Commons license, and indicate if changes were made.
In cases where reviewers are anonymous, credit should be given to 'Anonymous Referee' and the source.
The images or other third party material in this Peer Review File are included in the article's Creative Commons license, unless indicated otherwise in a credit line to the material. If material is not included in the article's Creative Commons license and your intended use is not permitted by statutory regulation or exceeds the permitted use, you will need to obtain permission directly from the copyright holder.
To view a copy of this license, visit https://creativecommons.org/licenses/by/4.0/

*We thank the reviewers for their positive, thoughtful, and constructive assessment of our manuscript. We were pleased that the reviewers recognized the quality of the cryo-EM structure and the potential importance of our integrative model for understanding norovirus NS3 function.*

*In response to the comments, we have substantially revised the manuscript and added new computational, biochemical, single-molecule, and virological data. These new data strengthen the main conclusions of the study and address the reviewers' key points regarding the oligomeric state of NS3, nucleic acid engagement, ATPase activity, and the predicted N-terminal pore architecture.*

*We also wish to draw the reviewers' attention to a very recent preprint describing a picornaviral 2C holoenzyme[1]. While the associated map and atomic model are not yet available for detailed comparison, the reported structure provides independent support for a translocase-like mode of RNA engagement by 2C-like AAA+ ATPases. We have therefore cited this work in the Discussion.*

*Our point-by-point responses are provided below.*

**REVIEWER COMMENTS**

Reviewer #1 (Remarks to the Author):

In this manuscript, Haas and colleagues present a structure based on cryo-EM SPA of the Human Norovirus NS3 protein, a RNA helicase belonging to the helicase superfamily (SF) 3 according to sequence alignments. The structure was determined for a truncated version lacking the N-terminal transmembrane domain.

In brief, the authors generated a DeltaN61-version of the protein carrying an additional hexamerization motif at the N-terminus. In absence of this motif, the authors did not observe hexers using mass photometry. The authors showed hydrolysis activity of nucleotides, preferably ATP and GTP, and inhibited nucleotide activity in presence of ATPgS. Subsequently the authors prepared cryo-EM grids followed by imaging and particle averaging and electron density fitting with an alpha fold 2 guess of the NS3 hexamer structure. The authors revealed potential RNA interacting residues and resolved a lock-washer overall architecture of the helicase domain. The authors present also single-molecule data to show ssRNA and dsRNA binding. With an alpha fold 3 model the authors perform integrative prediction of how NS3 could be arranged on a lipid membrane and pump ssRNA across the membrane for particle packing.

The structure is well described and convincing as well as in line with earlier structures of SF3 helicases also SF3 DNA helicases. The manuscript is well written, though I have a few questions that should be addressed prior to publication:

*We thank the reviewer for their careful assessment and positive comments on the structure and clarity of the manuscript. We have addressed each point below and revised the manuscript accordingly, including new data clarifying nucleic acid engagement and the role of oligomerization.*

- The authors report at the beginning of their study that the hexamerization motif cc-hex was required to see hexamerization of their truncated protein using mass photometry. Yet, this is a very artificial motif and already the individual monomers of the AAA+ enzyme should show a propensity for hexamerization. I was wondering why the authors preformed hexamerisation experiments in absence of ssRNA? For helicases, nucleic acid substrates often act as templating structures.

*We thank the reviewer for raising this important point. Indeed, nucleic acid substrates can stabilize higher-order assemblies in some helicases and translocases. Our initial mass photometry experiments were designed to define the intrinsic oligomeric state of the soluble engineered constructs and to test whether cc-hex was sufficient to generate a cryo-EM-compatible hexamer.*

*Although cc-hex is artificial, similar strategies have been used successfully to stabilize soluble, ATPase-active forms of membrane-associated AAA+ enzymes when native oligomerization determinants are removed[2,3]. We therefore view hex-ΔN61-NS3 as a tractable model of the NS3 motor core, rather than a complete substitute for full-length NS3.*

*To address whether RNA can promote assembly of ΔN61-NS3 in the absence of cc-hex, we added new magnetic tweezers, EMSA, and ATPase experiments. In the presence of ATP, ΔN61-NS3 showed only weak and inconsistent effects on ssRNA hairpin refolding, did not form detectable ssRNA complexes by EMSA, and remained ATPase-inactive even at the highest ssRNA concentration tested (revised Results, lines 292–308; Figure 5J–K; Supplementary Figure S13H–K; revised Methods, lines 574–590 and 737–817). By contrast, hex-ΔN61-NS3 reproducibly engaged ssRNA in magnetic tweezers and EMSA assays (revised Results, lines 285–298; Figure 5H–J; Supplementary Figure S13A–B,J).*

*Thus, under the conditions tested, ssRNA is not sufficient to promote formation of an ATPase-active higher-order ΔN61-NS3 assembly. We also now state more clearly that the soluble hexameric construct does not fully recapitulate native NS3 function, and that direct analysis of processive RNA transport will require full-length, membrane-embedded NS3 (revised Discussion, lines 397–406).*

- The authors use in the beginning of their studies Alphafold 2, at the end Alphafold 3. What would happen if for the initial prediction alpha fold 3 would be used?

*This is an interesting point. AlphaFold3 was not publicly available when the constructs were designed and the experimental work was initiated. In any case, using AlphaFold3 at the outset would not have changed the design of the hex-ΔN61-NS3 construct, as both AlphaFold2 and AlphaFold3 identify residue ~61 as the most logical boundary between the NTD and HBD.*

*However, AlphaFold3 provided improved confidence for the full-length N-terminal membrane-associated region, particularly when fatty acids were included to approximate a membrane environment. Had these predictions been available earlier, they may have prompted us to explore full-length purification and membrane-reconstitution strategies sooner.*

- I am also puzzled about the hydrolysis activity tests. It seems these assays were performed in absence of ssRNA, suggesting that the reported activity results from futile triphosphate hydrolysis. Did the authors test ssRNA stimulated nucleotide hydrolysis?

*We have now directly tested the effect of ssRNA on NS3 ATPase activity using a 50-nt poly(A) ssRNA substrate, chosen because norovirus genomes contain a 3' poly(A) tail. We measured ATP hydrolysis by both hex-ΔN61-NS3 and ΔN61-NS3 across increasing ssRNA concentrations (revised Results, lines 299–308; Figure 5K; revised Methods, lines 574–590).*

*ssRNA did not stimulate ATP hydrolysis by hex-ΔN61-NS3, but instead caused a modest dose-dependent reduction in activity. ΔN61-NS3 showed no detectable ATPase activity in either the absence or presence of ssRNA (Figure 5K). These data indicate that higher-order oligomerization is required for ATPase activity, and that ssRNA alone is not sufficient to stimulate nucleotide hydrolysis in the soluble construct.*

*We also found that ATP reduced the hex-ΔN61-NS3-induced delay in ssRNA hairpin refolding, suggesting that nucleotide binding and/or hydrolysis modulates RNA engagement rather than simply stabilizing an RNA-bound state (revised Results, lines 299–302; Supplementary Figure S13K; revised Methods, lines 790–817). We interpret these findings to mean that the engineered soluble hexamer supports ssRNA engagement and ATP turnover, but does not fully recapitulate the native N-terminal membrane-associated context required for productive coupling between RNA binding and ATP hydrolysis.*

- Along the same line the dose dependent reduced ATPase activity by addition of ATPgS suggests a significantly higher affinity to ATP, yet this might be different if ssRNA is present. If the hydrolysis is as tightly regulated one subunit at a time, a single ATPgS bound might already be efficient to inhibit the hydrolysis of subsequent subunits (see e.g. Schlierf ... Ha eLife 2019). This should be tested and can also verify the tightly-regulated ATP hydrolysis model suggested by the authors. (Side note: ATPgS affinity could be also determined with a displacement assay based on mant-ADP).

*We agree that defining how ATPγS occupancy affects sequential hydrolysis would be valuable, particularly for understanding ATPase coordination across the hexamer. However, our new ATPase data show that ssRNA does not stimulate ATP hydrolysis by hex-ΔN61-NS3 under the conditions tested, but instead modestly reduces activity in a concentration-dependent manner (revised Results, lines 299–308; Figure 5K; revised Methods, lines 574–590). An ATPγS competition experiment in the presence of ssRNA would therefore not provide a straightforward test of RNA-stimulated sequential hydrolysis in this soluble construct, because RNA itself shifts the enzyme towards a less active state. We have therefore not added this experiment. We agree that assays such as mant-ADP displacement will be valuable in future work to dissect nucleotide affinity and subunit coordination, particularly for full-length, membrane-associated NS3.*

- The authors probe the unwinding activity using a sophisticated magnetic tweezers assay. Unfortunately, the authors do not observe unwinding, though we remain unclear if the purified enzyme is at all unwinding competent. Does the hexer assemble on the short ssRNA, does it require a partial duplex, does it show translocation activity (could be probed using correlative imaging and force spectroscopy (e.g. C-Trap as developed in one of the co-authors institute or in the hairpin assay with mechanical hairpin opening and closing as reported in earlier studies by Croquette or Bensimon). Therefore I suggest simple unwinding assays based on forked, marked(fluorescent or 32P-) RNA substrates followed by quenching and gel electrophoresis if any information on unwinding should be reported herewith. The authors further conclude from their single molecule experiments ssRNA binding, which again would be more convincing to test using EMSA.

*We thank the reviewer for this suggestion. We added an independent gel-based RNA duplex unwinding assay using a fluorescently labelled RNA duplex with a 3' overhang, as suggested. No RNA duplex unwinding was detected for any NS3 construct under the conditions tested (revised Results, lines 310–318; Supplementary Figure S14B; revised Methods, lines 726–736). We have revised the text to state explicitly that hex-ΔN61-NS3 engages ssRNA and hydrolyses ATP, but does not show detectable RNA duplex unwinding activity in either the magnetic tweezers or gel-based assays (revised Results, lines 285–318; Figure 5I–K; Supplementary Figure S14).*

*To provide independent evidence for ssRNA binding, we also added EMSA experiments. These show detectable ssRNA complex formation with hex-ΔN61-NS3, but not ΔN61-NS3 (revised Results, lines 292–298; Figure 5J; Supplementary Figure S13J; revised Methods, lines 737–744). We also replaced the original MBP-hex control with an isolated synthetic cc-hex peptide in the magnetic tweezers assays. MBP-hex was a convenient initial control for the hexamerization module, but MBP is not present in the NS3 constructs used in these*

*experiments. We therefore considered that the weak refolding delay observed previously with MBP-hex could reflect nonspecific steric or multivalent effects of the MBP fusion and associated affinity tags, rather than an effect of the cc-hex peptide itself. Consistent with this, the isolated cc-hex peptide alone did not delay ssRNA refolding, whereas hex-ΔN61-NS3 did (revised Results, lines 285–291; Supplementary Figure S13A–C; revised Methods, lines 790–817).*

*Together, these new data support oligomerization-dependent ssRNA engagement by NS3, while distinguishing this binding activity from detectable duplex unwinding. We also now state more clearly that processive RNA transport or helicase activity may require full-length, membrane-associated NS3 and/or additional replication-complex components (revised Discussion, lines 397–406).*

- An additional question arises, from Figure S11, where the authors state that their single-molecule assays were performed in absence of ATP. How can this then lead to translocation/unwinding?

*We thank the reviewer for pointing out this ambiguity. The ATP-free hairpin refolding assay was not intended to demonstrate ATP-driven translocation or unwinding, but to test whether NS3 can engage exposed ssRNA and alter refolding dynamics (revised Results, lines 285–291; Figure 5H–I; Supplementary Figure S13A–C; revised Methods, lines 790–817).*

*By contrast, the magnetic tweezers helicase assay was performed in the presence of ATP and was designed to test directly for ATP-dependent RNA hairpin unwinding. Under these conditions, we did not detect unwinding by hex-ΔN61-NS3, even under assisting force (revised Results, lines 310–315; Supplementary Figure S14A; revised Methods, lines 752–789). We have revised the text to make this distinction explicit.*

*We also added ssRNA refolding experiments in the presence of ATP. ATP reduced the hex-ΔN61-NS3-induced refolding delay, suggesting that nucleotide binding and/or hydrolysis modulates the RNA-bound state in the soluble construct rather than simply promoting stable RNA association (revised Results, lines 299–302; Supplementary Figure S13K).*

- Similar, with the authors second set of single-molecule experiments they find indications for dsRNA binding, something which could and should be verified using EMSA. (the supplementary figure 11 was hard to read as the arrangement of panels was in a zig-zag fashion). Is there any biochemical reason for dsRNA binding? Is the binding of a hexamer or is it non-specific charge mediated backbone binding of a monomer? (Could be tested with their non-hex variant?) - though: It also seems that the dsRNA single-molecule data show a distinct long living collapsed state shortly below 0.5 µm. What is this state? How well defined is it? Can one correlate it with a sequence element or an unligated ssRNA exposure due to the sample preparation which is present at a distinct position in the RNA sequence? Even though the data quality is high, it reports no insights, thus at this stage the single-molecule data presented in the main text or supplementary information do not add to the manuscript and findings of the structure in my view, they rather confuse the reader.

*We agree that the dsRNA magnetic tweezers data were difficult to interpret mechanistically and risked distracting from the central conclusions. We have therefore removed these data from the revised manuscript. Instead, we have shifted the focus to ssRNA engagement (Figure 5), which is more directly aligned with our proposed RNA translocation model. We have also improved the clarity of the supplementary magnetic tweezers data (Supplementary Figure S13).*

Reviewer #2 (Remarks to the Author):

This manuscript reports a near-atomic resolution cryo-EM structure of the oligomeric norovirus NS3 protein in engineered, soluble form and bound to ATPγS, and integrates these data with AlphaFold3 predictions to propose a full-length, membrane-embedded model. The work addresses an enzyme of clear biomedical relevance and situates its activity within the context of viral replication organelles, offering a timely contribution to our understanding of norovirus replication.

Strengths

• The cryo-EM structure is of high quality and establishes the oligomeric state of NS3 at near-atomic resolution.

• The model confirms the canonical AAA+ helicase fold and lock-washer architecture consistent with translocation.

• The integration of cryo-EM data with AlphaFold3 modeling offers a coherent framework for considering how NS3 engages RNA in the context of membrane association.

• The study provides a structural basis for interpreting prior functional reports and contributes a valuable reference for the SF3 helicase family.

• The accompanying mutational analysis supports the structural findings and provides important functional context, strengthening the link between the proposed architecture and NS3 activity.

We thank the reviewer for their positive and thoughtful assessment of our manuscript.

Limitations, concerns, and suggestions

The experimental cryo-EM structure is confirmatory rather than fundamentally novel: it reproduces the fold and oligomeric state predicted by AlphaFold and already known from related SF3 helicases (e.g., SV40 LTag, papillomavirus E1). The lock-washer arrangement is also expected, as it has been observed in other SF3 helicases solved in the presence of nucleic acids. A notable difference is the helical bundle domain (HBD), which in NS3 appears asymmetric, unlike the planar N-tiers of other SF3 helicases that act as oligomerization domains. In this case, the authors plausibly propose that oligomer stabilization is instead mediated by the N-domain (absent in the cryo-EM reconstruction).

*We thank the reviewer for this balanced assessment. We agree that, at the tertiary-structure level, the NS3 AAA+ fold is well predicted by AlphaFold, as is increasingly the case for many structural biology projects. However, the oligomeric state, nucleotide-dependent architecture, asymmetric HBD arrangement, and details of the ATPase sites and pore-loop organization required experimental validation. Moreover, the structure, together with the reproducible workflow to generate soluble hexameric NS3, provides a platform for future studies of interactions with viral and host factors and for structure-guided inhibitor discovery. We therefore believe that these data provide a valuable resource for the field.*

*The mechanistic claims (hand-over-hand RNA translocation, dual RNA–protein translocation involving VPg) are inferred rather than demonstrated, as no RNA substrate is present in the structure. These should be presented as hypotheses consistent with the architecture rather than as findings. It would also be helpful to clarify the single-molecule data on RNA binding and provide direct biochemical evidence for RNA engagement (e.g., EMSA). Since AlphaFold predictions are presented with RNA, the authors could comment on predicted interfaces and whether these resemble interactions seen in other SF3 helicases.*

*We appreciate this suggestion and have revised the manuscript to present the RNA-transport model more cautiously. We now describe hand-over-hand RNA movement and possible VPg-linked RNA transport as hypotheses consistent with the NS3 architecture, rather than demonstrated mechanisms (revised Discussion, lines 397–406 and 456–468).*

*To strengthen and qualify this model, we added AlphaFold3 predictions with ssRNA and ssDNA. In the ssRNA models, RNA was recurrently positioned within the central pore, with I242 near the RNA path and the ribose 2'-OH close to the backbone carbonyl of D240 (revised Results, lines 272–284; Figure 5G; Supplementary Figure S12A,C). By contrast, equivalent ssDNA was not reproducibly positioned in the pore (Supplementary Figure S12B,D). Consistent with this, magnetic tweezers assays showed weaker effects on DNA hairpin refolding than on RNA hairpin refolding, supporting preferential engagement of ssRNA over ssDNA by the oligomeric construct (revised Results, lines 285–291; Figure 5I; Supplementary Figure S13D–G; revised Methods, lines 790–817).*

*We also added EMSA experiments showing that hex-ΔN61-NS3, but not ΔN61-NS3, forms a detectable complex with ssRNA (revised Results, lines 292–298; Figure 5J; Supplementary Figure S13J; revised Methods, lines 737–744). Together, these computational, biochemical, and single-molecule data support oligomerization-dependent ssRNA engagement by NS3, which is more directly aligned with the proposed RNA translocation model, while we now explicitly state that direct RNA translocation remains to be demonstrated (revised Discussion, lines 397–406).*

One potentially interesting feature in the structure is the apparent direct interaction of the pore loops with nucleotide cofactors, which has not been reported for E1, SV40, or CMG. The authors should discuss how this might influence their proposed translocation mechanism. Is the r two-loop architecture analogous to that described for CMG?

*We agree that the solvent-mediated connection between pore loop 2 and the nucleotide-binding site is interesting, as it provides a plausible route by which nucleotide state could influence pore-loop positioning and substrate engagement. The functional importance of this region is further supported by alanine substitutions in pore loop 2, which abolished ATPase activity (Supplementary Figure S10B).*

*We have now compared this region with the corresponding substrate-facing elements in porcine circovirus 2 Rep and papillomavirus E1. Although the canonical PS1βH β-hairpin is replaced by pore loop 2 in NS3, an equivalently positioned aspartate lies near the nucleotide-binding site in all three structures. We include this comparison in a new supplementary figure and comment briefly on the altered pore-loop architecture in the revised Results, without overinterpreting whether these residues have equivalent mechanistic roles (revised Results, lines 268–271; Supplementary Figure S11B–D).*

*We are cautious about drawing a direct mechanistic analogy with CMG, given the major differences in domain organization, accessory subunits, and substrate context. Instead, we now cite the recent FMDV 2C holoenzyme preprint as the more relevant comparison for a 2C-like ATPase engaging RNA through a central pore (revised Discussion, lines 465–468).*

Finally, the membrane-binding fold is not experimentally resolved here, and the proposed model diverges from earlier predictions (e.g., the MLKL-like fold reported in Nature 2023). The authors should discuss these differences and provide support for their model.

*We thank the reviewer for raising this important point. We have addressed it by adding both comparative AlphaFold3 modelling and functional mutagenesis.*

*First, we performed AlphaFold3 predictions for NS3 sequences from norovirus genogroups I–V using two approaches: a truncation-based strategy analogous to that used to propose the MLKL-like fold, and our full-length hexamer strategy, which preserves the complete N-terminal region and includes cofactor context. The truncation-based models were lower confidence and less consistent across genogroups, whereas the full-length models reproducibly predicted an N-terminal membrane-associated pore aligned with the AAA+ core (revised Results, lines 319–350; Figure 6A–F; Supplementary Figures S15–S16; revised Methods, lines 679–692). We now discuss this comparison explicitly in the revised Discussion (lines 423–440).*

*Second, guided by the full-length AF3 model, we introduced mutations into conserved residues within the predicted membrane-spanning region of MNV NS3 and tested their effect during virus recovery. Mutations predicted to disrupt the pore strongly reduced viral replication, whereas mutations predicted to be tolerated produced viable virus (revised Results, lines 356–365; Figure 6G; revised Methods, lines 700–716).*

*Together, these new computational and functional data support the importance of the predicted N-terminal membrane-associated pore, while we now present this model cautiously as a prediction that will require future structural validation in full-length, membrane-associated NS3.*

Experiments that could strengthen the manuscript:

1. Since the central mechanistic claims depend on RNA engagement, an RNA-bound cryo-EM reconstruction (even at modest resolution) would strongly substantiate the proposed translocation model and clarify the role of pore loops.

*We agree that an RNA-bound cryo-EM reconstruction would be informative. However, stabilising a defined RNA-bound state would require substantial additional optimisation and would ideally be pursued with full-length, membrane-associated NS3, which we are now investigating. We have therefore not added an RNA-bound reconstruction in this revision.*

*Instead, we strengthened the RNA-engagement model using new AlphaFold3 RNA/DNA predictions, magnetic tweezers RNA/DNA comparisons, EMSA, RNA-dependent ATPase assays, and RNA duplex unwinding assays (revised Results, lines 272–318; Figure 5G–K; Supplementary Figures S12–S14; revised Methods, lines 574–590, 679–692, and 726–817). Together, these data support oligomerization-dependent ssRNA engagement by NS3, while we now state explicitly that direct RNA translocation remains to be demonstrated (revised Discussion, lines 397–406).*

*We also note that a recent FMDV 2C holoenzyme preprint reported ssRNA threaded through the central pore of a split-ring AAA+ hexamer[1], providing an independent and relevant comparison for our NS3 model (revised Discussion, lines 465–468).*

2. The central channel is reported as negatively charged, which would disfavor stable nucleic acid binding. The suggestion of transient interactions is compelling but could be tested explicitly, for example by RNAse protection assays.

*We thank the reviewer for this suggestion. This is an experiment that we will definitely consider for future studies of the full-length complex, if we are able to establish a robust purification and membrane-reconstitution system. We believe such experiments would be more informative in the context of full-length, membrane-associated NS3, as we do not yet know how fusion of the heterologous hexamerization domain affects RNA binding relative to the authentic membrane-embedded protein.*

Minor comments

1. The reported map resolution may not support unambiguous water placement throughout. Please avoid strong claims of water-mediated interactions unless the density is clear; otherwise, mark such waters as putative.

We have revised the manuscript to describe ordered water molecules as putative where appropriate, unless their assignment is unambiguous, such as waters coordinating $Mg^{2+}$ in the ATP-binding site

2. Lines 556–557: Please specify the EM grid material (gold or copper) and the mesh size.

We have added the missing cryo-EM grid material and mesh size to the revised Methods (lines 625–632).

3. Lines 570–572: Please indicate the particle diameter used for template picking.

We have added the particle diameter used for template picking to the revised Methods (lines 640–645).

4. Line 602: Correct "secondary restraints" to "secondary structure restraints."

*This has now been corrected (line 675).*

Reviewer #3 (Remarks to the Author):

I co-reviewed this manuscript with one of the reviewers who provided the listed reports. This is part of the Nature Communications initiative to facilitate training in peer review and to provide appropriate recognition for Early Career Researchers who co-review manuscripts.

We thank the co-reviewer for their contribution to the peer review of our manuscript.

Reviewer #4 (Remarks to the Author):

This manuscript aims to provide a semi-predictive model of the norovirus NTPase/Helicase protein NS3, and to propose a molecular model whereby the function of the protein is to export a ssRNA viral RNA molecule across a cellular membrane via pore. The NS3 protein is known to be multi-functional and the authors here provide the first structural data for how NS3 may form multimers to assemble a functional complex. The cryo-EM is combined with predictive AF3 modelling that overall is convincing in revealing a hexamer of the proteins that assembles on a cellular membrane. Supporting data is provided in defining key residues that support the individual subunit interactions and within associated lipid and nucleotide molecules. Overall and very informative study with some aspects to be addressed to completely support the conclusions made.

We are grateful to the reviewer for their positive assessment and constructive suggestions, which have helped us improve the manuscript.

Major issues to be addressed:

- One of the key aspects of this project is whether the hexameric form of NS3 is the authentic

form observed in infected cells. Of course, this is challenging to determine but is critical to the overall conclusions described here.

- The authors should attempt to purify full length NS3 from transfected cells, after mild detergent and affinity purification followed by SEC and native PAGE gel. Alternative would be to use chemical cross-linking in cells and isolate NS3

*We agree that establishing the biological relevance of the hexameric assembly is important. Previous studies have reported oligomerization of norovirus NS3 in cells[4], supporting the plausibility of an oligomeric functional state. Direct purification of full-length, membrane-associated NS3 is an ongoing direction in our laboratory, but it requires substantial membrane-protein biochemistry and lies beyond the scope of this revision. In particular, detergent solubilization alone may not preserve the native N-terminal architecture, and reliable analysis of the full-length complex may require reconstitution in nanodiscs or liposomes.*

*To address this point, we added two complementary analyses. First, AlphaFold3 modelling across NS3 sequences from norovirus genogroups I–V showed that interface confidence increased with subunit number and peaked at the hexameric state, supporting a hexamer as the preferred full-length assembly model (revised Results, lines 335–342; Supplementary Figure S15E–G; revised Methods, lines 679–692). Second, we introduced targeted mutations into MNV NS3 based on the predicted hexameric N-terminal pore and assessed viral replicative fitness. Mutations predicted to disrupt key pore features strongly impaired viral replication, whereas mutations predicted to be tolerated yielded viable virus (revised Results, lines 356–365; Figure 6G; revised Methods, lines 700–716).*

*While these data do not directly visualize hexameric NS3 in infected cells, they provide new computational and functional support for the biological relevance of the predicted hexameric assembly.*

- So just to confirm, the monomeric or dimeric MBP-NS3 did not have ATPase activity? i.e. was the oligomeric hexameric form strictly required for this activity? Did the other oligomeric forms ie. trimer, tetramer, pentamer etc also have ATPase activity, and to what degree? Does incubation of the MBP-NS3, rather than hex-NS3, with ATPgS stabilize any additional oligomeric structures?

*We thank the reviewer for asking us to clarify this point. In mass photometry and native mass spectrometry, ΔN61-NS3 lacking cc-hex was observed predominantly as lower-order species, including monomers, dimers, and trimers. However, this construct showed no detectable ATPase activity either without RNA or with ssRNA at the highest concentration tested. By contrast, hex-ΔN61-NS3 contained a strong hexameric component and displayed robust ATPase activity (Figure 1B–D; Supplementary Figure S3; revised Results, lines 299–308; Figure 5K). These data support the conclusion that lower-order ΔN61-NS3 species are not sufficient for ATP hydrolysis, and that higher-order oligomerization is required to generate an ATPase-active NS3 motor.*

*We also note that cc-hex promotes higher-order oligomerization but does not enforce a completely homogeneous hexameric population. Because these oligomeric states appear to exist in dynamic equilibrium, we are not able to isolate stable trimeric, tetrameric, or pentameric species for separate ATPase analysis.*

- In Fig 3, what was the rationale for choosing the selected mutants? I understand that these were to disrupt cation-interaction and helix-turn-helix motif but the selected amino acids don't completely correspond with those identified in the structure. So, the rationale seems disconnected from the structure. I would suggest to complement the study by introducing the

corresponding amino acids from GI e.g. D292 with E and K/R to confirm that the charge is required.

*We apologise that the rationale for these mutations was not sufficiently clear. We have revised the manuscript to clarify the correspondence between the GI NS3 sequence used for cryo-EM/biochemistry and the GV MNV NS3 sequence used for virological assays. In particular, there is a one-residue offset between these sequences, which caused an apparent mismatch between residues highlighted in the structure and those mutated in the MNV infectious clone (Supplementary Figure S9; revised figure legends for Figures 3 and 4).*

*The GV NS3 D292P and V304P mutations were designed to disrupt helices involved in the predicted structural interface. R294A and Y333A correspond to GI residues R293 and F332, respectively, and were designed to disrupt the predicted cation–π interaction, whereas V301A was included as a mutation predicted to be structurally tolerated (revised Results, lines 194–203; Figure 3H–I). We have clarified this rationale in the revised text and figure legends.*

*We also added biochemical analysis of eight additional GI hex-NS3 mutants. Mutations predicted to disrupt key structural features caused complete or near-complete loss of ATPase activity, supporting the link between the structural model, enzymatic activity, and viral fitness (revised Results, lines 194–203 and 220–244; Figures 3I and 4D,H; Supplementary Figure S10B).*

- Additionally, the impact observed on MNV replication should be extended beyond just TCID50. The authors should also measure genome amplification via qRT-PCR and protein production via western blot to determine if these mutations are affecting early events (as predicted) rather than just virus release.

*We thank the reviewer for this suggestion. We have now extended the virological analysis beyond TCID50/CPE-based readouts by adding qRT-PCR analysis for a subset of the NS3 mutants. Mutations that impaired virus recovery also reduced viral RNA accumulation, supporting the conclusion that these defects occur at the level of genome amplification and/or early replication events, rather than simply affecting virus release (revised Results, lines 194–203 and 220–226; Supplementary Figure S10A). Although we did not perform western blot analysis, the qRT-PCR experiments provide an independent measure of viral RNA accumulation and strengthen the link between the NS3 structural model, ATPase activity, and viral replication.*

- How do the mutations introduced into MNV in Fig 3I (and Fig 4) affect the overall predicted structure of the NS3 hexamer? Do all of the mutations in MNV also affect the ATPase activity? This needs to be confirmed to relate structure and function

*As noted above, we have now generated additional GI hex-NS3 mutants corresponding to residues analysed in the MNV system and tested their ATPase activity directly. Mutations predicted to disrupt key structural features caused complete or near-complete loss of ATPase activity, supporting the link between the structural model, viral fitness, and enzymatic function (revised Results, lines 194–203 and 220–244; Supplementary Figure S10B).*

- In Fig 5B and C do the DNA helicases have an NTD that is also membrane associated? Essentially asking if the lack of interactions between the NS3 HBDs in this model are due to the absence of the membrane?

*The membrane-associated N-terminal region is a feature of 2C-like proteins from the order Picornavirales and is not present in the DNA virus SF3 helicases shown in Figure 5B,C. In these soluble DNA helicases, the N-tier/HBD contributes directly to oligomerization. By contrast, our predicted NTD model, supported by mutational analysis, suggests that NS3 uses*

*a distinct architecture in which the N-terminal membrane-associated region provides a major oligomerization interface, while the HBDs do not form the same extensive inter-subunit contacts. We cannot exclude that additional HBD contacts may form in the full-length, membrane-embedded protein or in the presence of RNA. However, the reduced HBD-HBD interface observed in the cryo-EM structure is consistent with a genuine architectural difference between NS3 and the soluble DNA virus SF3 helicases, rather than simply resulting from the absence of a membrane.*

- For the molecular tweezers experiments was the monomeric/dimeric forms of NS3 also tested? It would be interesting to consider the compartmentalisation of function with the helicase activity more “internal” to the viral replication complex and potentially coordinated via an interaction with Pol or ProPol on the dsRNA template

*We have now tested ΔN61-NS3, which lacks the cc-hex oligomerization module and is predominantly lower order by mass photometry and native mass spectrometry. In contrast to hex-ΔN61-NS3, which reproducibly delayed ssRNA hairpin refolding, ΔN61-NS3 showed only weak and inconsistent effects (revised Results, lines 285–298; Figure 5I–J; Supplementary Figure S13H–J; revised Methods, lines 790–817). These data support the conclusion that robust ssRNA engagement in this assay depends on higher-order NS3 assembly.*

*We agree that NS3 function may be spatially organized within the viral replication complex and could involve coordination with Pol or ProPol on structured RNA templates. Direct testing of this possibility would require reconstitution of additional replication-complex components and is beyond the scope of this revision. To place our data in this biological context, we have added a model in Figure 7 proposing that the AAA+ domain lies on the cytosolic face of the membrane, consistent with directional transport of positive-sense ssRNA for translation or encapsidation and with the earlier model of luminal dsRNA within single-membrane vesicles proposed by Hyde et al[5]. (revised Discussion, lines 441–468; Figure 7).*

**References**


1. Pfuetzner, R. A. *et al.* The Structure of the Picornaviral 2C:RNA holoenzyme: Molecular Basis of RNA binding and specificity by a AAA+ protein. *bioRxiv* 2026.06.07.730651 (2026) doi:10.64898/2026.06.07.730651.
2. Shi, H., Rampello, A. J. & Glynn, S. E. Engineered AAA+ proteases reveal principles of proteolysis at the mitochondrial inner membrane. *Nat. Commun.* **7**, (2016).
3. Puchades, C. *et al.* Structure of the mitochondrial inner membrane AAA+ protease YME1 gives insight into substrate processing. *Science (1979).* **358**, (2017).
4. Wang, G., Zhang, D., Orchard, R. C., Hancks, D. C. & Reese, T. A. Norovirus MLKL-like protein initiates cell death to induce viral egress. *Nature* **616**, (2023).
5. Hyde, J. L. *et al.* Mouse Norovirus Replication Is Associated with Virus-Induced Vesicle Clusters Originating from Membranes Derived from the Secretory Pathway. *J. Virol.* **83**, (2009).
